# The APOE-R136S mutation protects against APOE4-driven Tau pathology, neurodegeneration and neuroinflammation

**DOI:** 10.1038/s41593-023-01480-8

**Published:** 2023-11-13

**Authors:** Maxine R. Nelson, Peng Liu, Ayushi Agrawal, Oscar Yip, Jessica Blumenfeld, Michela Traglia, Min Joo Kim, Nicole Koutsodendris, Antara Rao, Brian Grone, Yanxia Hao, Seo Yeon Yoon, Qin Xu, Samuel De Leon, Tenzing Choenyi, Reuben Thomas, Francisco Lopera, Yakeel T. Quiroz, Joseph F. Arboleda-Velasquez, Eric M. Reiman, Robert W. Mahley, Yadong Huang

**Affiliations:** 1 Gladstone Institute of Neurological Disease, Gladstone Institutes, San Francisco, CA USA; 2 Biomedical Sciences Graduate Program, University of California, San Francisco, San Francisco, CA USA; 3 Gladstone Institute of Data Science and Biotechnology, Gladstone Institutes, San Francisco, CA USA; 4 Neuroscience Graduate Program, University of California, San Francisco, San Francisco, CA USA; 5 Developmental and Stem Cell Biology Graduate Program, University of California, San Francisco, San Francisco, CA USA; 6 Gladstone Center for Translational Advancement, Gladstone Institutes, San Francisco, CA USA; 7 Grupo de Neurociencias de Antioquia de la Universidad de Antioquia, Medellin, Colombia; 8 Departments of Neurology and Psychiatry, Massachusetts General Hospital and Harvard Medical School, Boston, MA USA; 9 Schepens Eye Research Institute of Mass Eye and Ear and Department of Ophthalmology, Harvard Medical School, Boston, MA USA; 10 Banner Alzheimer’s Institute, Phoenix, AZ USA; 11 University of Arizona, Tucson, AZ USA; 12 Department of Pathology, University of California, San Francisco, San Francisco, CA USA; 13 Department of Medicine, University of California, San Francisco, San Francisco, CA USA; 14 Department of Neurology, University of California, San Francisco, San Francisco, CA USA

**Keywords:** Alzheimer's disease, Neurodegeneration

## Abstract

Apolipoprotein E4 (*APOE4*) is the strongest genetic risk factor for late-onset Alzheimer’s disease (LOAD), leading to earlier age of clinical onset and exacerbating pathologies. There is a critical need to identify protective targets. Recently, a rare APOE variant, APOE3-R136S (Christchurch), was found to protect against early-onset AD in a PSEN1-E280A carrier. In this study, we sought to determine if the R136S mutation also protects against APOE4-driven effects in LOAD. We generated tauopathy mouse and human iPSC-derived neuron models carrying human APOE4 with the homozygous or heterozygous R136S mutation. We found that the homozygous R136S mutation rescued APOE4-driven Tau pathology, neurodegeneration and neuroinflammation. The heterozygous R136S mutation partially protected against APOE4-driven neurodegeneration and neuroinflammation but not Tau pathology. Single-nucleus RNA sequencing revealed that the APOE4-R136S mutation increased disease-protective and diminished disease-associated cell populations in a gene dose-dependent manner. Thus, the APOE-R136S mutation protects against APOE4-driven AD pathologies, providing a target for therapeutic development against AD.

## Abstract (web summary)

Nelson et al. report that the APOE-R136S mutation protects against APOE4-promoted Alzheimer’s disease pathologies, including phosphorylated Tau accumulation, neuroinflammation and neurodegeneration, in mouse and human neuron models.

## Main

Apolipoprotein E4 (*APOE4*) is the strongest genetic risk factor for late-onset Alzheimer’s disease (LOAD)^1–3^. It exacerbates AD-related pathologies, including amyloid-beta (Aβ) plaques, Tau tangles, neurodegeneration and neuroinflammation^4–16^. As APOE4 carriers make up 55–75% of AD cases^17–20^, there is a critical need to investigate the roles of APOE4 in AD pathogenesis and to identify protective targets to mitigate its detrimental effects.

APOE is involved in lipid metabolism and highly expressed in the brain^21^, where it is primarily produced in astrocytes but also in neurons and microglia in response to stress^22–28^. APOE has two functional domains: an amino-terminal receptor-binding region (residues 136–150) and a carboxyl-terminal lipid-binding region (residues 244–275)^2,29,30^. A single residue change between APOE4(Arg112) and APOE3(Cys112) isoforms alters protein structure and function in both domains^3,30–35^. The receptor-binding region is enriched with positively charged amino acids and binds with negatively charged cell surface receptors, including heparin sulfate proteoglycans (HSPGs)^30,35–37^. The C-terminal domain of APOE weakly binds with HSPGs, and it also indirectly modifies the function of the N-terminal receptor-binding domain^30^. Therefore, mutations in both regions can directly or indirectly alter the binding of APOE to various cell surface receptors^35–37^. Variants of APOE with point mutations near or within these regions, such as APOE2(Cys112, Cys158)^18,38^, APOE3-V236E^39,40^ and APOE4-R251G^40^, are associated with protection against AD.

A recent discovery that a rare APOE variant, APOE3-R136S (APOE3-Christchurch)^36^, strongly protects against early-onset Alzheimer’s disease (EOAD) highlights the importance of studying rare variants of APOE in AD pathogenesis and protection. The reported patient was protected from the clinical effects of PSEN1-E280A, a highly penetrant mutation causing EOAD dementia^41,42^, for 28 years by the homozygous APOE3-R136S mutation^36^. This patient displayed extremely high Aβ pathology but minimal Tau burden, hippocampal atrophy and neuroinflammation^36,43,44^. A major functional effect of the R136S mutation is disruption of its binding to anionic HSPGs on cell surface^36,45^. Intriguingly, over the past decade, HSPGs have been revealed as essential players in the cellular uptake of Tau^46–48^. Tau accumulation and propagation during AD progression correlate with neurodegeneration and clinical effects^49^, which are major targets in the development of disease-modifying therapies for AD^50,51^. The differential contributions of APOE isoforms to Tau pathology, neurodegeneration and neuroinflammation are of great interest, yet the underlying mechanisms remain unclear. The discovery of AD-protective APOE-R136S mutation raises a fundamental question as to whether the R136S mutation can also protect against APOE4-driven pathologies in LOAD.

To address this question, we engineered the R136S mutation into the *APOE4* allele background both in vivo in human APOE4 knock-in (E4-KI) mice^52^ and in vitro in human induced pluripotent stem cells (hiPSCs) derived from a patient with AD with homozygous APOE4 (ref. ^6^). To investigate the effects of the R136S mutation in vivo under a disease-relevant condition, we cross-bred isogenic E4-KI and E4-R136S-KI mice with a widely used tauopathy mouse model expressing Tau-P301S (PS19 line)^53^. Using these PS19-APOE mouse and isogenic hiPSC models, in which any pathological changes are due solely to the introduction of this mutation overcoming APOE4 effects, we show that the R136S mutation robustly protects against APOE4-driven AD pathologies, and we present potential mechanisms underlying these effects.

## Results

### Generating tauopathy mice expressing human APOE4 or APOE4-R136S

**Fig. 1:**
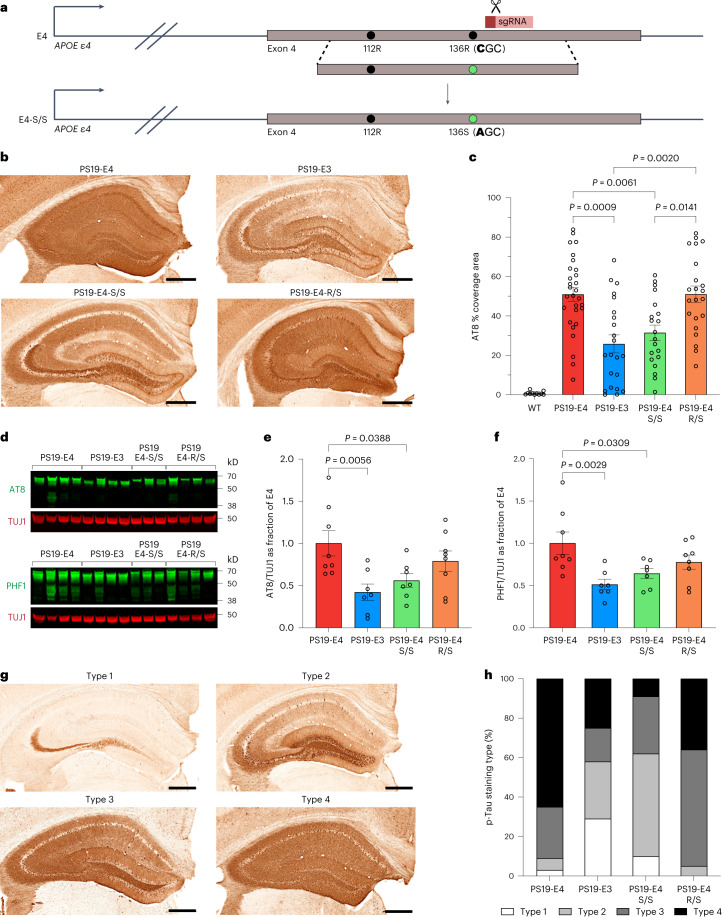
Homozygous R136S mutation rescues APOE4-promoted Tau pathology in tauopathy mice. **a**, Schematic of CRISPR–Cas-9-mediated gene editing strategy to generate human APOE4-R136S knock-in mice. **b**, Representative images of p-Tau immunostaining in hippocampus of 10-month-old PS19-E4, PS19-E3, PS19-E4-S/S and PS19-E4-R/S mice with the AT8 monoclonal antibody. **c**, Quantification of the percent AT8 coverage area in the hippocampus of these mice (PS19-E4, *n* = 29; PS19-E3, *n* = 22; PS19-E4-S/S, *n* = 20; PS19-E4-R/S, *n* = 22) and WT mice (*n* = 11). **d**, Representative western blot images with p-Tau-specific AT8 or PHF1 antibody. TUJ1 was used as a loading control. **e**,**f**, Quantification of AT8^+^ (**e**) and PHF1^+^ (**f**) p-Tau levels in hippocampal lysates of PS19-E4 (*n* = 8), PS19-E3 (*n* = 7), PS19-E4-S/S (*n* = 7) and PS19-E4-R/S (*n* = 8) mice. p-Tau levels were normalized to TUJ1 first and then to those of PS19-E4 mice. **g**, Representative images of four AT8 staining patterns in the hippocampus. **h**, Distribution of four p-Tau staining patterns in the hippocampus of 10-month-old PS19-E4, PS19-E3, PS19-E4-S/S and PS19-E4-R/S mice (PS19-E4, *n* = 29; PS19-E3, *n* = 22; PS19-E4-S/S, *n* = 20; PS19-E4-R/S, *n* = 22). Scale bars in **b** and **g**, 500 µm. Throughout, data are expressed as mean ± s.e.m. Differences between groups were determined by Welch’s ANOVA followed by Dunnett’s T3 multiple comparison test (**c**) or ordinary one-way ANOVA followed by Dunnett’s multiple comparison test (**e**,**f**). Comparisons of *P* ≤ 0.05 are labeled on the graph. Source data

**Extended Data Fig. 1:**
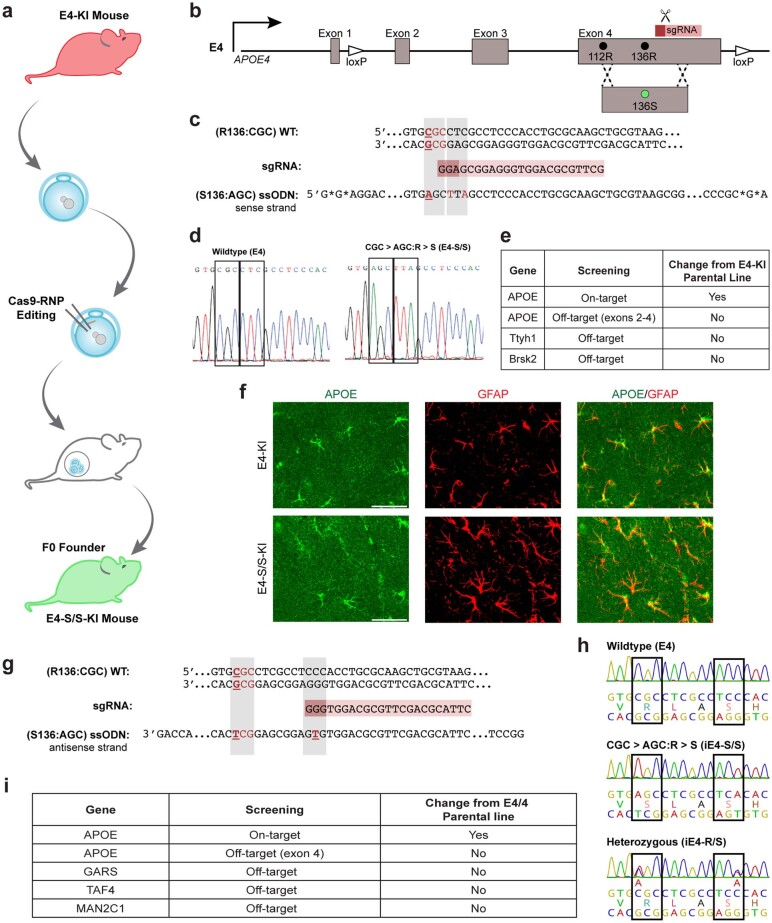
Generation of human APOE4-R136S knock-in mice and APOE4-R136S hiPSC lines by CRISPR/Cas-9-mediated gene editing. **a**, Schematic of generating human APOE4-R136S knock-in (E4-S/S-KI) mice using CRISPR/Cas-9-mediated gene editing. **b**, Schematic of gene editing strategy to generate the R136S mutation in human E4-KI mice (with the loxP sites, unused in current gene-editing strategy) and to generate APOE4-R136S (E4-S/S) hiPSC lines (without the loxP sites). **c**, DNA sequences of WT human *APOE4* loci encoding for R136, designed sgRNA, and single-stranded oligodeoxynucleotides donor repair template encoding for S136 and silent mutation at PAM site for generating E4-S/S-KI mice. **d**, Sanger DNA sequencing of WT *APOE4* and *APOE4-S/S* at and near the site encoding for residue 136 in E4-S/S-KI mice. **e**, Summary of on-target R136S editing in APOE4 and potential off-target mutation screening for knock-in mice. **f**, Representative immunofluorescent images of APOE (green) and GFAP (red) in CA1 hippocampal subfield in E4-KI and E4-S/S-KI mice at 12 months of age (scale bar, 50 μm). **g**, DNA sequences of WT *APOE4* loci encoding for R136, designed sgRNA, and single-stranded oligodeoxynucleotides donor repair template encoding for S136 and silent mutation at PAM site for generating E4-S/S hiPSC lines. **h**, Sanger DNA sequencing of WT E4, E4-S/S, and E4-R/S at and near the site encoding for residue 136 in hiPSC lines. **i**, Summary of on-target R136S editing in APOE4 and potential off-target mutation screening in hiPSC lines. Experiments depicted in representative images in f were performed on n = 3 mice per genotype using 2 brain sections per mouse, with reproducible data. WT, wildtype; sgRNA, single guide RNA; ssODN, single-stranded oligodeoxynucleotide.

To determine if the R136S mutation also protects against the detrimental effects of APOE4 in LOAD, we generated homozygous and heterozygous human APOE4-R136S-KI mice (Fig. 1a and Extended Data Fig. 1a−d), referred to as E4-S/S-KI and E4-R/S-KI mice, respectively. We used CRISPR–Cas-9-mediated gene editing to introduce the R136S mutation into the *APOE4* locus in human E4-KI mice that were previously generated in our laboratory^52^. Genomic DNA (gDNA) sequencing verified on-target gene editing of R136 to S136 in APOE4 (Extended Data Fig. 1d) and found no changes within the top predicted potential off-target sites (Extended Data Fig. 1e). APOE and glial fibrillary acidic protein (GFAP) double immunostaining confirmed high APOE expression in astrocytes of E4-S/S-KI mice (Extended Data Fig. 1f), identical to that seen in astrocytes of the parental E4-KI mice (Extended Data Fig. 1f). For detailed APOE expression characterization, also see single-nucleus RNA sequencing (snRNA-seq) analysis below.

Reduced Tau pathology was a critical feature of the reported human case with resistance to autosomal-dominant EOAD, although amyloid pathology was exceedingly high in this individual^36,43^. Thus, we cross-bred the E4-KI and E4-S/S-KI mice with a tauopathy mouse model, PS19 (ref. ^53^). Four groups of mice with different APOE genotypes—PS19-E4, PS19-E4-S/S, PS19-E4-R/S and PS19-E3 (as control)—were used in this study. All mice were analyzed at an average age of 10 months to allow for the development of key AD pathologies^7^.

### E4-S/S reduces Tau pathology in tauopathy mice

**Extended Data Fig. 2:**
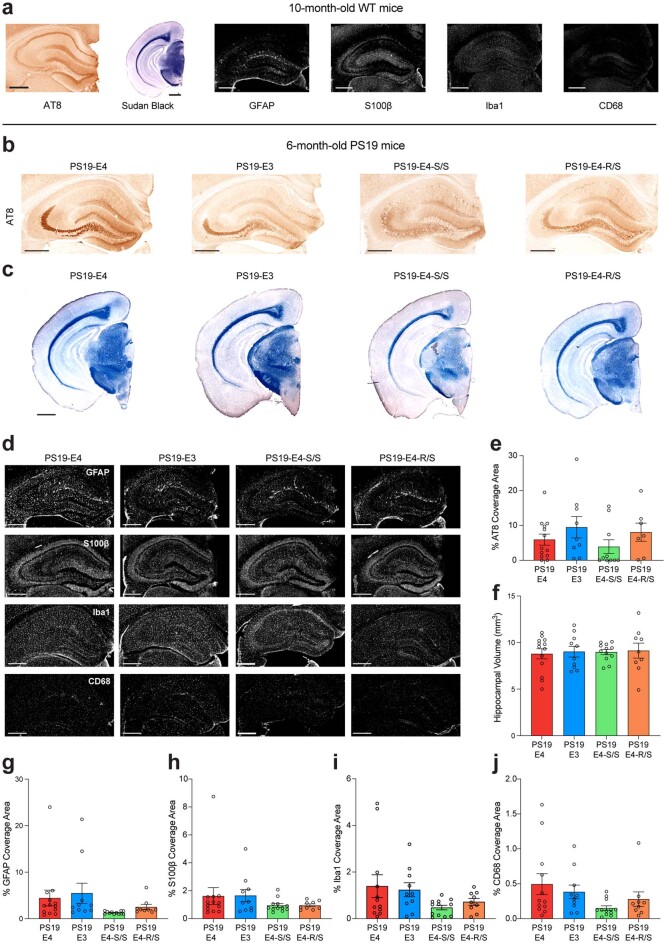
Histopathological analyses of 10-month-old WT and 6-month-old PS19-E3, PS19-E4, PS19-E4-S/S, and PS19-E4-R/S mice. **a**, Representative images of 10-month-old WT mouse (n = 11) brain sections stained with AT8 monoclonal antibody to visualize p-Tau (scale bar, 500 µm), Sudan black to enhance hippocampal visualization (scale bar, 1 mm), GFAP and S100β to measure astrocytosis (scale bar, 500 µm), and Iba1 and CD68 to measure microgliosis (scale bar, 500 µm). **b,c**, Representative images of 6-month-old PS19-E4, PS19-E3, PS19-E4-S/S, and PS19-E4-R/S mouse brain sections stained with AT8 antibody for p-Tau (scale bar, 500 µm) (b) or Sudan black (scale bar, 1 mm) (c). **d**, Representative images of GFAP and S100β immunostaining for astrocytes and reactive astrocytes, respectively, as well as Iba1 and CD68 immunostaining for microglia and reactive microglia, respective, in the hippocampus of 6-month-old PS19-E4, PS19-E3, PS19-E4-S/S, and PS19-E4-R/S mice. Scale bar, 500 µm. **e–j**, Quantification of % AT8 coverage area (e), hippocampal volume (f), % GFAP coverage area (g), % S100β coverage area (h), % Iba1 coverage area (i), and % CD68 coverage area (j). In a–j, PS19-E4, n = 12; PS19-E3, n = 11; PS19-E4-S/S, n = 12; PS19-E4-R/S, n = 9; n=mice. Experiments depicted in representative images in a–d were performed using 2 brain sections per mouse, with reproducible data. Throughout, data are expressed as mean ± s.e.m. Differences between groups were determined by ordinary one-way ANOVA followed with Dunnett’s multiple comparison test (e,f) or Welch’s ANOVA followed with Dunnett T3 multiple comparison test (g-j); comparisons of p ≤ 0.05 were labeled on graph. Source data

With these tauopathy mouse models, we first examined the levels of phosphorylated Tau (p-Tau) accumulation in the hippocampus by immunocytochemistry with an AT8 antibody. We observed heterogeneity in the percent AT8 coverage area within each group. This level of heterogeneity in hippocampal Tau pathology has been reported as a feature of age in the PS19 model^54^ and has also been reported in studies using PS19 mice crossed with human E4-KI or E3-KI mice^7,55^. PS19-E4 mice displayed roughly two-fold higher AT8^+^ p-Tau coverage area than PS19-E3 mice (Fig. 1b,c), as reported previously^7,55^. Remarkably, PS19-E4-S/S mice exhibited markedly reduced p-Tau accumulation, roughly to the levels in PS19-E3 mice (Fig. 1b,c). This reduction was not seen in PS19-E4-R/S mice (Fig. 1b,c), suggesting that the protection of APOE4-driven p-Tau accumulation in the hippocampus requires the homozygous R136S mutation. Unsurprisingly, we observed minimal AT8 immunopositivity in 10-month-old wild-type (WT) control mice (Fig. 1c and Extended Data Fig. 2a). No significant difference was observed in p-Tau pathology across various APOE genotype groups of 6-month-old mice, although PS19-E4-S/S mice had a trend toward lower p-Tau pathology (Extended Data Fig. 2b,e). Hippocampal lysates of PS19-E4 mice had significantly higher p-Tau levels than those of PS19-E3 mice at 10 months of age, as determined by western blotting with AT8 and PHF1 antibodies (Fig. 1d–f). Again, PS19-E4-S/S mice, but not PS19-E4-R/S mice, had significantly reduced p-Tau levels as compared to PS19-E4 mice at 10 months of age (Fig. 1d–f).

Previous studies reported distinct p-Tau staining patterns as functions of progressive tauopathy in PS19-E mice with different APOE genotypes^7,11^. We observed similar categories of p-Tau staining (Fig. 1g). Type 1 displays AT8 positivity in mossy fibers and hilus with occasional sparse CA3 and somatic staining; type 2 has additional dense, tangle-like staining in dentate gyrus (DG) granule cells (GCs), some staining in CA3 pyramidal cells and sparse neurite staining in the CA1 region; type 3 shows further staining in the stratum radiatum of the CA region, primarily neurite staining, with dense staining in only some of the soma; and type 4 has further staining over the entire hippocampus.

The most advanced type 4 p-Tau staining was enriched in PS19-E4 mice, with roughly 90% of sections analyzed showing type 3 or type 4 p-Tau staining (Fig. 1h). The early p-Tau staining types 1 and 2 were enriched in PS19-E3 mice (Fig. 1h), as seen previously^7^. The PS19-E4-S/S mice displayed the lowest fraction of type 4 p-Tau staining (~10%) and showed similar levels of enrichment of type 1 and type 2 staining to PS19-E3 mice (Fig. 1h). Notably, the PS19-E4-R/S mice still showed roughly 90% type 3 or type 4 p-Tau staining (Fig. 1h), with a slight preference for type 3 over type 4 p-Tau staining as compared to PS19-E4 mice, possibly indicating a slight slowing of APOE4-driven progression of p-Tau pathology.

As another measurement of p-Tau pathology, we quantified the number of AT8^+^ soma in the cell layer of the hippocampal CA1 subregion of 10-month-old mice. Both PS19-E4 and PS19-E4-R/S mice had significantly more AT8^+^ soma in CA1 than PS19-E3 and PS19-E4-S/S mice (Supplementary Fig. 1a). Notably, the number of AT8^+^ soma in the CA1 cell layer strongly correlated with the percent AT8 coverage area in the hippocampus (Supplementary Fig. 1b). Together, these data indicate that the R136S homozygosity is required to effectively protect against APOE4-driven p-Tau accumulation and progression of p-Tau staining patterns in this tauopathy mouse model.

### Generating isogenic E4-S/S and E4-R/S hiPSC lines

To determine the mechanisms underlying the protection of the R136S mutation against APOE4-driven Tau pathology, we further modeled it in hiPSC-derived neurons. A parental APOE4/4 hiPSC line was previously generated from a patient with AD homozygous for the *APOE4* allele, which provided a human and disease-relevant cellular model^6^. From this APOE4/4 hiPSC line, we generated isogenic hiPSC lines with either the homozygous (E4-S/S) or the heterozygous (E4-R/S) R136S mutation using CRISPR–Cas-9-mediated gene editing (Extended Data Fig. 1g,h). gDNA sequencing verified the correct editing of R136 to S136 in APOE4 (Extended Data Fig. 1h) and found no mutations in the top predicted potential off-target sites (Extended Data Fig. 1i).

**Extended Data Fig. 3:**
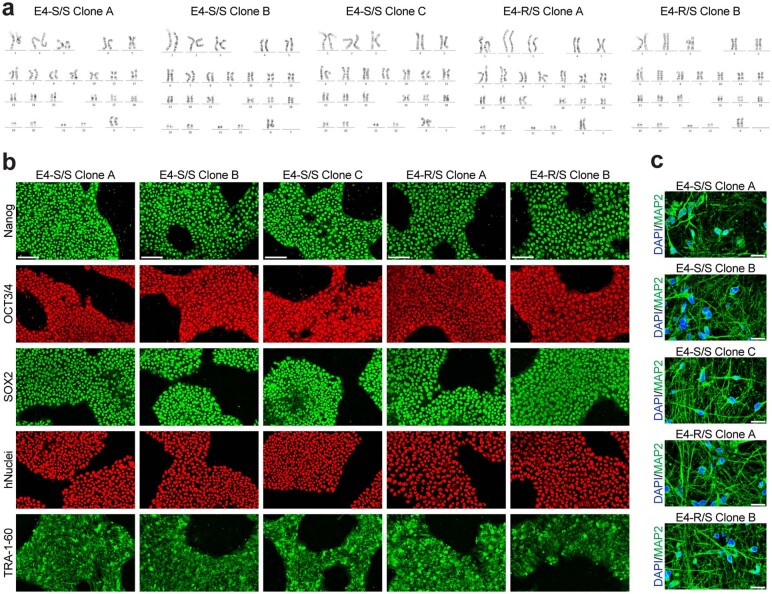
Characterization of E4-S/S and E4-R/S hiPSC lines and neuronal differentiation. **a**, Karyogram of E4-S/S-A, E4-S/S-B, E4-S/S-C, E4-R/S-A, and E4-R/S-B hiPSC lines. For all cell lines, metaphase was examined for n = 20 cells per line. **b**, Representative immunofluorescent images of pluripotent stem cell markers Nanog, OCT3/4, SOX2, hNuclei, and TRA-1-60 from E4-S/S-A, E4-S/S-B, E4-S/S-C, E4-R/S-A, and E4-R/S-B hiPSC lines (scale bar, 100 μm). **c**, Representative immunofluorescent images of mature neuronal marker MAP2 (green) and DAPI (blue) in 3-week-old neurons differentiated from E4-S/S-A, E4-S/S-B, E4-S/S-C, E4-R/S-A, and E4-R/S-B hiPSC lines (scale bar, 20 μm). Representative images were selected from n = 3 fields of view from each cell line.

Five stable, isogenic hiPSC lines (three E4-S/S clones and two E4-R/S clones) were karyotyped to ensure no chromosomal abnormalities (Extended Data Fig. 3a). Additionally, they all expressed stem cell markers Nanog, OCT3/4, SOX2 and TRA-1-60 (Extended Data Fig. 3b). hiPSC lines were then differentiated into neurons expressing mature neuronal marker MAP2 (Extended Data Fig. 3c) via an optimized method using dual-SMAD inhibition^6,56^. Both the E4-S/S and E4-R/S neuronal cultures showed normal morphology, neurite outgrowth pattern and viability (Extended Data Fig. 3c). We then used the following isogenic hiPSC-derived neurons for further analyses: the parental E4 hiPSC line (E4), a previously generated isogenic APOE3 hiPSC line (E3)^6^ and the isogenic E4-S/S and E4-R/S hiPSC lines. Immunocytochemistry analysis confirmed that MAP2^+^ neurons expressed APOE (Supplementary Fig. 2a), as reported previously^6^. The neuronal cultures were also validated negative, via immunocytochemistry, for non-neuronal cell types, such as neural stem cells (SOX2), astrocytes (GFAP) and oligodendrocytes (Olig2) (Supplementary Fig. 2a,b). As reported previously^6^, the E4 neuron culture had a lower fraction of GABA^+^ inhibitory neurons than the E3 neuron culture (Supplementary Fig. 2c,d). The E4-S/S neuron cultures showed a similar proportion of GABA^+^ inhibitory neurons to the E3 neuron cultures (Supplementary Fig. 2c,d), suggesting that the R136S mutation may reduce APOE4’s detrimental effect on GABAergic neurons. The E4-S/S neurons also had a lower ratio of APOE fragment/full-length APOE than E4 neurons at both the individual hiPSC line level and the combined hiPSC line level (Supplementary Fig. 2e,f), suggesting that the R136S mutation results in less APOE4 fragmentation^6^.

### E4-S/S reduces p-Tau accumulation in human neurons

**Fig. 2:**
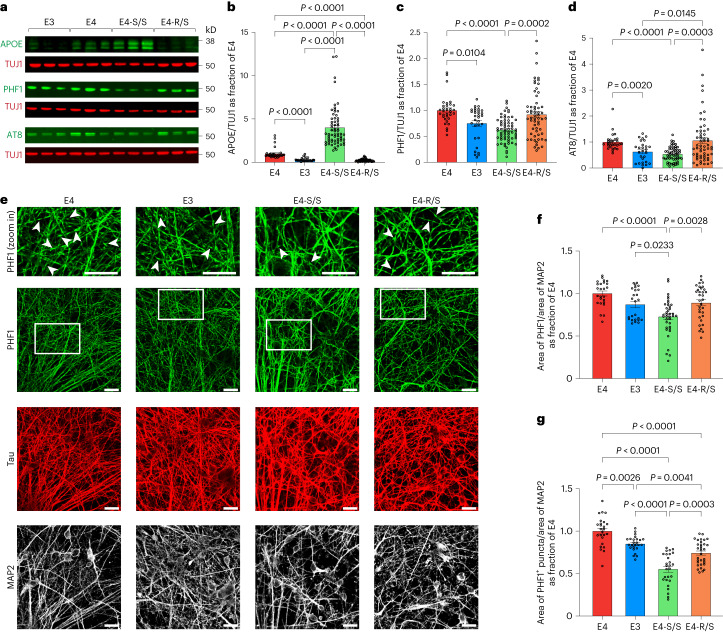
Homozygous R136S mutation protects against APOE4-induced p-Tau accumulation in human neurons. **a**–**d**, Representative western blot images (**a**) and quantification of APOE (**b**), PHF1^+^ p-Tau (**c**) and AT8^+^ p-Tau (**d**) levels in lysates of E4, E3, E4-S/S or E4-R/S neurons. In **b**, APOE levels were normalized to those of E4. TUJ1 was used as loading control (E4, *n* = 32; E3, *n* = 31; E4-S/S, *n* = 64 (*n* = 24 from E4-S/S-A; *n* = 8 from E4-S/S-B; *n* = 32 from E4-S/S-C); E4-R/S, *n* = 59 (*n* = 31 from E4-R/S-A; *n* = 28 from E4-R/S-B)). In **c**, PHF1^+^ p-Tau levels were normalized to those of E4. TUJ1 was used as loading control (E4, *n* = 32; E3, *n* = 31; E4-S/S, *n* = 64 (*n* = 24 from E4-S/S-A; *n* = 8 from E4-S/S-B; *n* = 32 from E4-S/S-C); E4-R/S, *n* = 59 (*n* = 31 from E4-R/S-A; *n* = 28 from E4-R/S-B)). In **d**, AT8^+^ p-Tau levels were normalized to those of E4. TUJ1 was used as loading control (E4, *n* = 28; E3, *n* = 27; E4-S/S, *n* = 55 (*n* = 16 from E4-S/S-A; *n* = 11 from E4-S/S-B; *n* = 28 from E4-S/S-C); E4-R/S, *n* = 59 (*n* = 31 from E4-R/S-A; *n* = 28 from E4-R/S-B)). **e**, Representative images showing immunostaining of p-Tau (PHF1), total Tau and MAP2 in E4, E3, E4-S/S or E4-R/S human neurons, with some PHF1^+^ puncta (white arrowheads in insets). **f**,**g**, Quantification of fraction of the PHF1^+^ area over MAP2^+^ area (**f**) and fraction of PHF1^+^ puncta area over MAP2^+^ area (**g**) (E4, *n* = 25 fields of view; E3, *n* = 25 fields of view; E4-S/S, *n* = 38 fields of view; E4-R/S, *n* = 33 fields of view). The ratio of PHF1^+^ area (**f**) and PHF1^+^ puncta area (**g**) over MAP2 area was normalized to that of E4. Western blot data (**b**–**d**) were made up of at least three independent rounds of differentiation, and all data were combined. Scale bars (**e**), 20 µm. In **b**–**d**, *n* = biological replicates. Throughout, data are expressed as mean ± s.e.m. Differences between groups were determined by Welch’s ANOVA followed by Dunnett’s T3 multiple comparison test (**b**–**d**,**g**) or ordinary one-way ANOVA followed by Tukey’s multiple comparison test (**f**). Comparisons of *P* ≤ 0.05 are labeled on the graph. Source data

The effect of the R136S mutation in human neurons was first assessed by quantification of APOE levels via western blotting. The E4-S/S neurons had a five-fold increase in APOE protein levels compared to E4 neurons (Fig. 2a,b and Supplementary Fig. 3a). Because we did not observe a similar phenotype in PS19-E4-S/S mouse hippocampal lysates (Supplementary Fig. 4), this is likely an across-species difference, which warrants further study. The E4-R/S neurons did not show such an effect on APOE protein levels (Fig. 2a,b and Supplementary Fig. 3a).

Western blotting analysis of cell lysates with PHF1 and AT8 antibodies revealed that the E3 neurons had a significant reduction in p-Tau levels compared to E4 neurons (Fig. 2a,c,d and Supplementary Fig. 3b,c), confirming our previous report^6^. The E4-S/S neurons showed an approximately 40% reduction in p-Tau levels compared to E4 neurons at both the combined hiPSC line level (Fig. 2c,d) and the individual hiPSC line level (Supplementary Fig. 3b,c). Notably, E4-R/S neurons did not show the same reduction of p-Tau levels (Fig. 2a,c,d and Supplementary Fig. 3b,c).

Likewise, immunocytochemical staining with the PHF1 antibody also revealed an approximately 35% reduction in p-Tau levels in E4-S/S neurons versus E4 neurons (Fig. 2e,f), whereas no such effect was seen in E4-R/S neurons (Fig. 2e,f). We also observed some beading patterns in PHF1^+^ neurites, suggesting p-Tau aggregates and/or degenerating neurites, which were the least prevalent in E4-S/S neurons (Fig. 2e, arrowheads, and Fig. 2g). Taken together, these data illustrate that only the homozygous R136S mutation protects against APOE4-induced p-Tau accumulation in human neurons, in line with the in vivo observations in tauopathy mice (Fig. 1).

### E4-S/S reduces HSPG-mediated Tau uptake by human neurons

Recent findings show that cell surface HSPGs mediate a large portion of neuronal uptake of Tau monomers and aggregates^46,57^. Additionally, neuronal uptake of exogenous Tau in culture can be competitively inhibited with the addition of heparin^46,57^. Notably, the APOE-R136S has severely reduced binding affinity to heparin, as the mutation occurs within the receptor-binding region of APOE^36,45,58^. By contrast, APOE4 has increased binding affinity (up to two-fold) for heparin and HSPG over APOE3 (refs. ^32–35^). We hypothesized that the E4-S/S-reduced p-Tau accumulation in human neurons was, in part, due to its defective HSPG binding and, consequently, the reduced Tau uptake promoted by APOE4 via HSPGs.

**Fig. 3:**
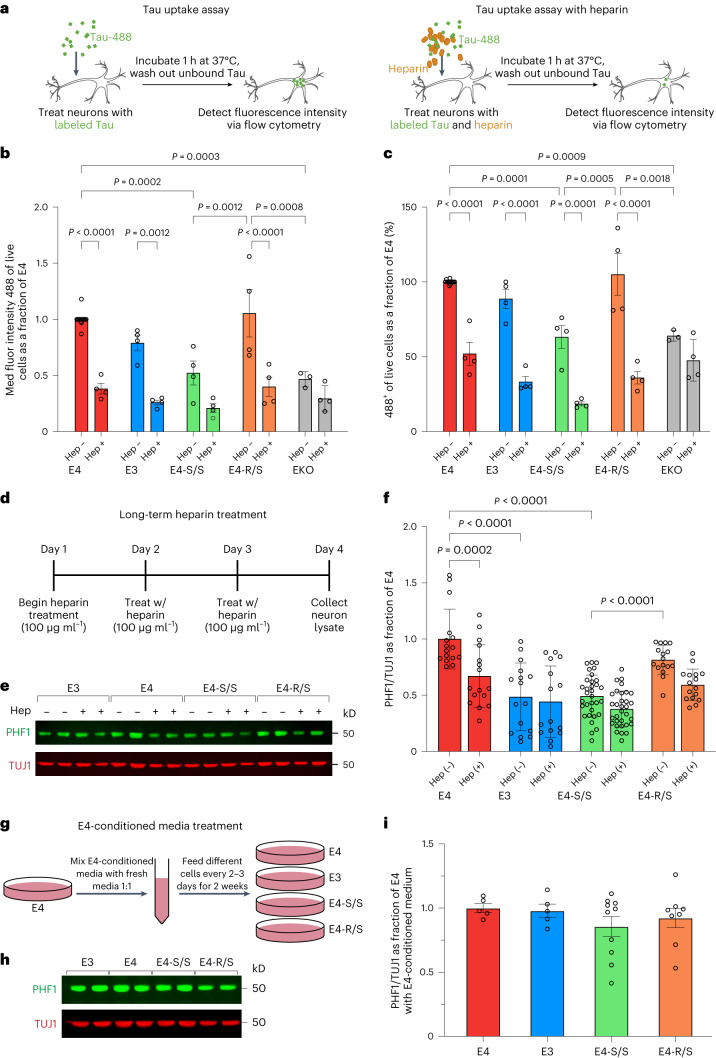
Homozygous R136S mutation protects against APOE4-induced p-Tau accumulation by reducing Tau uptake via the HSPG pathway. **a**, Diagram of Tau-488 uptake assay. Neurons treated with either Tau-488 alone (left) or Tau-488 together with 100 µg ml^−1^ heparin (right) before flow cytometry analysis. **b**, Measurement of individual neuronal Tau-488 uptake (25 nM, 1-h incubation) based on MFI per cell in human neurons. **c**, Measurement of Tau-488 uptake (25 nM, 1-h incubation) based on percent Tau-488^+^ human neurons. In **b**,**c**, *n* = independent experiments and normalized to E4 MFI (**b**) or uptake (%) (**c**). E4, *n* = 13; E4+heparin, *n* = 4; E3, *n* = 4; E3+heparin, *n* = 4; E4-S/S, *n* = 4; E4-S/S+heparin, *n* = 4; E4-R/S, *n* = 4; E4-R/S+heparin, *n* = 4; EKO, *n* = 3; EKO+heparin, *n* = 4. Analysis was performed on a live cell population of estimated 5,000 cells for each sample. **d**, Experimental design for long-term heparin treatment of human neurons. **e**,**f**, Representative western blot images (**e**) and quantification of PHF1^+^ p-Tau levels (**f**) in lysates of E4, E3, E4-S/S or E4-R/S neurons under long-term heparin treatment. In **f**, PHF1^+^ p-Tau levels were normalized to those of E4. TUJ1 was used as loading control (E4, *n* = 16; E4+heparin, *n* = 16; E3, *n* = 16; E3+heparin, *n* = 15; E4-S/S, *n* = 32; E4-S/S+heparin, *n* = 32; E4-R/S, *n* = 16; E4-R/S+heparin, *n* = 16). **g**, Experimental design for E4 neuron-conditioned medium treatment of human neurons with different APOE genotypes. **h**,**i**, Representative western blot images (**h**) and quantification of PHF1^+^ p-Tau levels (**i**) in lysates of E4, E3, E4-S/S or E4-R/S neurons after E4 neuron-conditioned medium treatment. In **i**, PHF1^+^ p-Tau levels were normalized to those of E4. TUJ1 was used as loading control (E4, *n* = 5; E3, *n* = 5; E4-S/S, *n* = 10; E4-R/S, *n* = 10). In **f**,**i**, *n* =biological replicates. Throughout, data are expressed as mean ± s.e.m. Differences between groups were determined by two-way ANOVA followed by Tukey’s multiple comparison test (**b**,**c**,**f**) or ordinary one-way ANOVA followed by Tukey’s multiple comparison test (**i**). Some comparisons of *P* ≤ 0.05 are labeled on the graph. Hep, heparin; fluor, fluorescence. Source data

**Extended Data Fig. 4:**
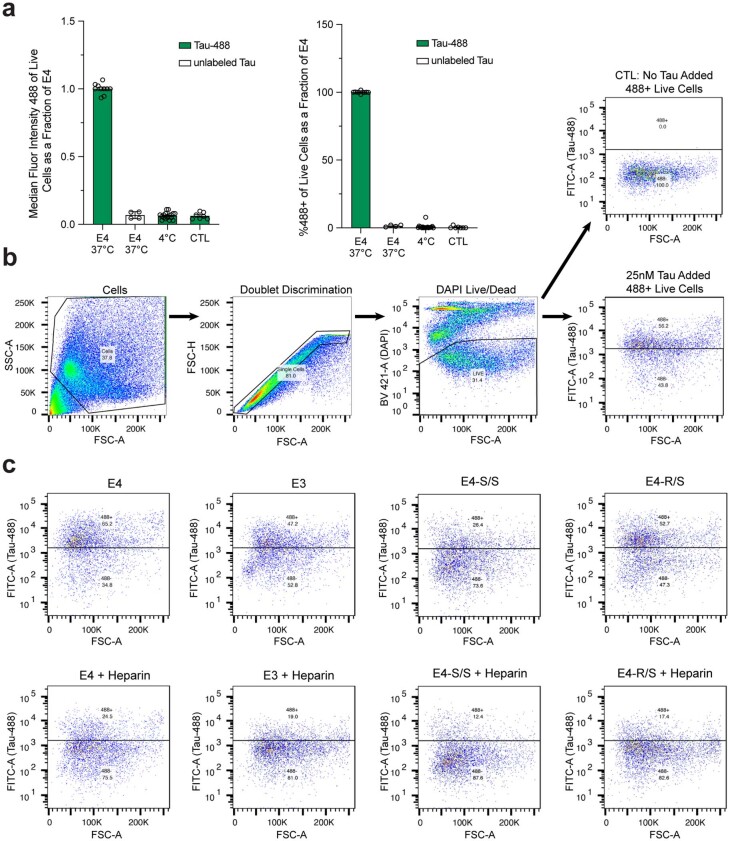
Measurement of neuronal uptake of Tau-488 by flow cytometry. **a**, Internal controls of Tau-488 uptake assay as measured by median fluorescent intensity at 488 nm of live cell population (left) or % 488-positive live cell population (right) via flow cytometry. All values normalized to E4 cells at 37 °C, collected over separate experiments. Samples treated with Tau-488 are colored in green and samples treated with unlabeled tau are colored in white. E4 at 37 °C with Tau-488, n = 10; E4 at 37 °C with unlabeled Tau, n = 4; E4 at 4 °C with Tau-488, n = 18; E4 at 37 °C no Tau CTL, n = 7; n=independent experiment with unique biological samples. **b**, Gating strategy for Tau uptake assay. First, cells were gated on forward scatter/side scatter (FSC/SSC). Cells were then gated on forward scatter height (FSC-H) versus area (FSC-A) to discriminate doublets. Dead cells were removed from the analysis using nuclear stain with DAPI, and positive cells were determined by gating on a control (no Tau-488 added) population. **c**, Scatter plots of flow cytometry analysis of live cell population at 488 nm (FITC-A versus FSC-A) for E4, E3, E4-S/S, or E4-R/S neuronal cultures with or without the treatment with 100 µg/ml heparin. Roughly 1×10^5^ to 5×10^5^ events were recorded in each experiment. The live cell population analyzed was roughly 5000 cells for each sample. In a, data are expressed as mean ± s.e.m. Source data

To test this hypothesis, we assessed the effects of E4-S/S and E4-R/S on Tau uptake using human neurons. We first labeled recombinant Tau protein (2N4R) with Alexa Fluor 488 fluorophore (Tau-488) and then treated neuronal cultures with 25 nM Tau-488 (Fig. 3a), as reported previously^46,47,57^. Flow cytometry was used to determine the differences in the internalization of exogenous Tau-488 by human neurons with different APOE genotypes. Neurons were incubated for 1 h at 37 °C with either Tau-488 alone or Tau-488 together with 100 µg ml^−1^ heparin to inhibit Tau uptake via an HSPG-dependent pathway. We repeated the experiments for both conditions at 4 °C to prevent endocytosis so as not to conflate signal from surface-bound Tau-488 with internalized Tau-488. The detectable signal at 4 °C was negligible at this concentration of Tau-488 and incubation time (Extended Data Fig. 4a), as was previously reported^46^. We also treated E4 neurons with 25 nM unlabeled recombinant Tau and performed the same uptake experiment as a control to ensure that the 488 signal was not due to Tau incubation-induced cellular changes, such as reactive oxygen species (ROS) production, that could lead to autofluorescence (Extended Data Fig. 4a).

We analyzed the median fluorescence intensity (MFI), reflecting the median level of Tau-488 uptake at an individual cell level, in neurons with different APOE genotypes (Fig. 3b and Extended Data Fig. 4b,c). The E4-S/S neurons exhibited an approximately 50% reduction in MFI compared to E4 neurons (Fig. 3b), indicating reduced Tau-488 uptake. The E3 neurons also showed a trend of reduced Tau-488 uptake (~20%) compared to E4 neurons, although not reaching significance. The E4-R/S neurons showed no significant difference in MFI versus E4 neurons (Fig. 3b), suggesting no alteration in Tau-488 uptake. To clarify the role of APOE in Tau uptake by neurons, we also included an APOE knockout (EKO) human iPSC line that we generated previously^6^. The EKO neurons showed a marked decrease in Tau-488 uptake versus E4 neurons, to roughly the level of E4-S/S neurons (Fig. 3b). This suggests that (1) the APOE-independent Tau-488 uptake by EKO neurons occurs at a similar level to E4-S/S neurons and (2) the receptor-binding-defective R136S mutation diminishes the ability of APOE to functionally contribute to Tau-488 uptake in E4-S/S neurons, mimicking EKO neurons.

Heparin treatment significantly reduced MFI in E4, E3 and E4-R/S neurons (Fig. 3b), whereas the treatment led only to a trend toward lower MFI in E4-S/S neurons (Fig. 3b), indicating already decreased HSPG-dependent Tau uptake in E4-S/S neurons. The EKO neurons treated with heparin showed a similarly small decrease (Fig. 3b), which represents the APOE-independent but HSPG-dependent Tau-488 uptake. Notably, E4 neurons treated with heparin exhibited reduction of MFI (~40%) to levels not significantly different from E4-S/S neurons and EKO neurons without heparin treatment. Furthermore, we quantified the percentage of Tau-488^+^ cells, reflecting the proportion of cells with detectable levels of the internalized Tau-488 across the entire culture (Fig. 3c). Only E4-S/S and EKO neurons had a significantly reduced fraction (~30%) of cells with internalized Tau-488, as compared to E4 neurons (Fig. 3c). It is again notable that E4 neurons treated with heparin reduced the fraction of neurons with internalized Tau-488 to levels seen in E4-S/S neurons and EKO neurons without heparin treatment. The effect of heparin treatment on the fraction of neurons with internalized Tau-488 was similar in E4 and E4-R/S neurons (Fig. 3c). Taken together, these data suggest that E4-S/S and EKO neurons, but not E4-R/S neurons, have similarly reduced Tau uptake compared to E4 neurons, which may be due to the defective HSPG binding of E4-S/S and the lack of APOE, respectively.

### Defective HSPG binding of E4-S/S protects against p-Tau accumulation

We next tested if the lowered HSPG-dependent Tau uptake, as seen after 1-h incubation with recombinant Tau in E4-S/S neurons, could contribute to protection against APOE4-induced endogenous p-Tau accumulation in human neurons. We designed an assay to measure the relative long-term effect of heparin treatment on endogenous levels of p-Tau in neuronal cultures (Fig. 3d). hiPSC-derived neurons were treated with 100 µg ml^−1^ heparin daily for three full days and then analyzed for p-Tau (Fig. 3d,e). Remarkably, this treatment lowered endogenous p-Tau levels in E4 neurons by approximately 25%, similar to those seen in untreated E4-S/S neurons (Fig. 3f). Notably, heparin treatment did not significantly alter endogenous p-Tau levels in E4-S/S neurons (Fig. 3f). These data suggest that the defective HSPG binding of the R136S mutation may contribute to the reduced APOE4-driven Tau accumulation in human neurons.

To further test this possibility, we asked whether the presence of E4 neuron-conditioned medium would diminish the APOE-isoform-dependent effects on p-Tau accumulation in various human neurons (Fig. 3g). In fact, treatment with E4 neuron-conditioned medium (1:1 mixed with fresh medium) for 2 weeks increased p-Tau accumulation in both E3 and E4-S/S neurons to the level of E4 neurons (Fig. 3h,i). Thus, the presence of exogenous APOE4, even with much higher endogenous APOE4-S/S levels (Fig. 2a,b), was sufficient to increase p-Tau accumulation in E4-S/S neurons to levels similar to those seen in E4 neurons. Taken together, these data support the hypothesis that homozygous R136S mutation protects against APOE4-induced p-Tau accumulation at least in part due to defective HSPG binding.

### E4-S/S ameliorates neurodegeneration in tauopathy mice

In addition to limited Tau tangle burden, previous reports of a PSEN1 mutation carrier with the APOE3-R136S mutation also described protection from other AD pathologies, including neurodegeneration and neuroinflammation^36,43,44^. These pathologies can also be exacerbated in the presence of APOE4 in LOAD^4,7,59^. Thus, we sought to determine if the R136S mutation protects against APOE4-promoted neurodegeneration and neuroinflammation in PS19-E mice.

**Fig. 4:**
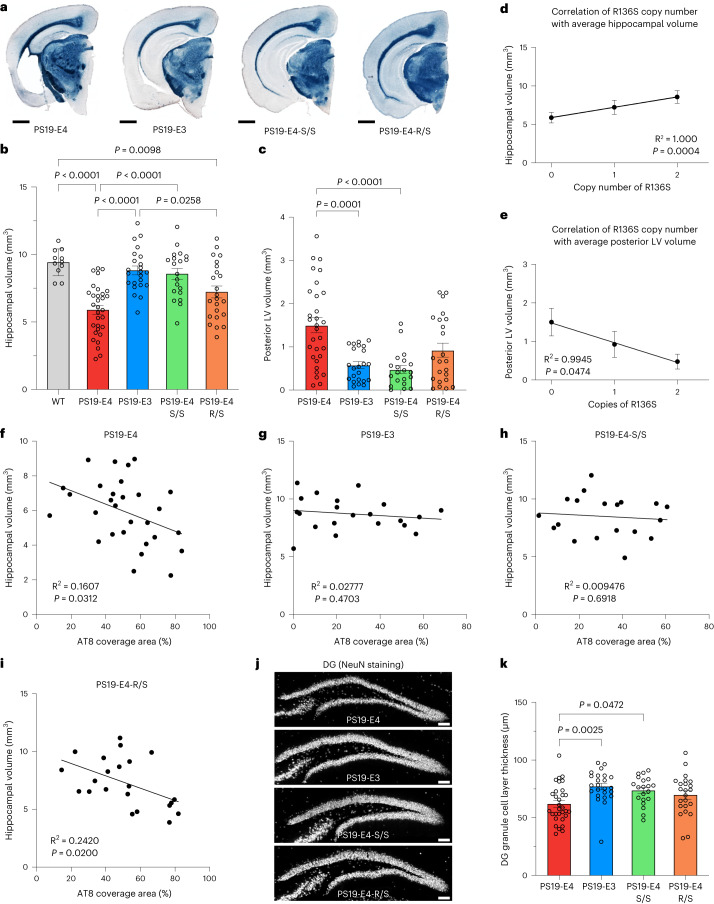
The R136S mutation ameliorates APOE4-driven neurodegeneration in tauopathy mice. **a**, Representative images of 10-month-old PS19-E4, PS19-E3, PS19-E4-S/S and PS19-E4-R/S mouse brain sections stained with Sudan black to enhance hippocampal visualization (scale bar, 1 mm). **b**,**c**, Quantification of hippocampal volume (WT, *n* = 11; PS19-E4, *n* = 31; PS19-E3, *n* = 23; PS19-E4-S/S, *n* = 20; PS19-E4-R/S, *n* = 23; *n* = mice) (**b**) and posterior lateral ventricle volume (PS19-E4, *n* = 30; PS19-E3, *n* = 23; PS19-E4-S/S, *n* = 20; PS19-E4-R/S, *n* = 23; *n* = mice) (**c**). **d**,**e**, Correlation of the APOE4-R136S gene copy number with the average of hippocampal volume (PS19-E4, *n* = 31; PS19-E4-S/S, *n* = 20; PS19-E4-R/S, *n* = 23; *n* = mice) (**d**) or the average of posterior lateral ventricle volume (PS19-E4, *n* = 30; PS19-E4-S/S, *n* = 20; PS19-E4-R/S, *n* = 23; *n* = mice) (**e**) in PS19-E4 mice with 0, 1 or 2 copies of the APOE4-R136S gene mutation. **f**–**i**, Correlations between percent AT8 coverage area and hippocampal volume in 10-month-old PS19-E4 (*n* = 29) (**f**), PS19-E3 (*n* = 21) (**g**), PS19-E4-S/S (*n* = 19) (**h**) and PS19-E4-R/S (*n* = 22) (**i**) mice. **j**, Representative images of the DG stained for NeuN in 10-month-old PS19-E4, PS19-E3, PS19-E4-S/S and PS19-E4-R/S mice (scale bar, 100 μm). **k**, Quantification of DG GC layer thickness in 10-month-old PS19-E4 (*n* = 30), PS19-E3 (*n* = 23), PS19-E4-S/S (*n* = 20) and PS19-E4-R/S (*n* = 23) mice. Throughout, data are expressed as mean ± s.e.m. except for correlation plots. Differences between groups were determined by ordinary one-way ANOVA followed by Tukey’s multiple comparison test (**b**,**k**) or Welch’s ANOVA followed by Dunnett’s T3 multiple comparison test (**c**). Comparisons of *P* ≤ 0.05 are labeled on the graph. Pearson’s correlation analysis (two-sided). LV, lateral ventricle. Source data

We evaluated the extent of neurodegeneration in 10-month-old PS19-E mice with different APOE genotypes and in WT mice. PS19-E4 mice exhibited significantly reduced hippocampal volume compared to WT and PS19-E3 mice (Fig. 4a,b and Extended Data Fig. 2a), as reported previously^7,55^. Strikingly, hippocampal atrophy of PS19-E4-S/S mice was reduced to similar levels of those seen in WT and PS19-E3 mice (Fig. 4a,b). We also observed a partial rescue of hippocampal atrophy in PS19-E4-R/S mice toward PS19-E3 levels (Fig. 4b). No significant difference was observed in hippocampal volume across various APOE genotype groups of 6-month-old mice (Extended Data Fig. 2c,f).

Furthermore, 10-month-old PS19-E4 mice displayed significantly enlarged lateral ventricles compared to PS19-E3 mice, and this was protected in PS19-E4-S/S mice (Fig. 4a,c). Again, the PS19-E4-R/S mice had a partial rescue (Fig. 4c). Analysis of correlation between the APOE4-R136S copy number (0, 1 or 2) and the average of hippocampal volume or the average of posterior lateral ventricle volume revealed a clear gene dose-dependent effect of the R136S mutation on the protection of APOE4-promoted hippocampal degeneration (Fig. 4d,e).

In 10-month-old PS19-E4 mice, the AT8 (p-Tau) percent coverage area was negatively correlated with hippocampal volume (Fig. 4f), as shown previously^7,55^, indicating a deleterious role of p-Tau in hippocampal degeneration. Similarly, the number of AT8^+^ soma in CA1 cell layer was negatively correlated to hippocampal volume in PS19-E4 mice (Supplementary Fig. 1c). These relationships disappeared in PS19-E3 and PS19-E4-S/S mice (Fig. 4g,h and Supplementary Fig. 1d,e), suggesting that milder Tau pathology in these mice might not contribute to hippocampal degeneration. These relationships were still present in PS19-E4-R/S mice (Fig. 4i and Supplementary Fig. 1f), suggesting that the lack of protection against p-Tau pathology in PS19-E4-R/S mice (Fig. 1c and Supplementary Fig. 1a) could be a contributing factor to neurodegeneration in these mice.

We also assessed neurodegeneration by measuring the thickness of the NeuN^+^ GC layer of the DG (Fig. 4j). PS19-E3 and PS19-E4-S/S mice showed a significant increase (~20%) in DG GC layer thickness over PS19-E4 mice (Fig. 4k). Again, PS19-E4-R/S mice showed a non-significant increase (12%) in DG GC layer thickness over PS19-E4 mice. Taken together, these data indicate that the R136S mutation ameliorates APOE4-driven neurodegeneration in the tauopathy mice, with the homozygous mutation being required for a full rescue.

### E4-S/S reduces astrocytosis in tauopathy mice

**Fig. 5:**
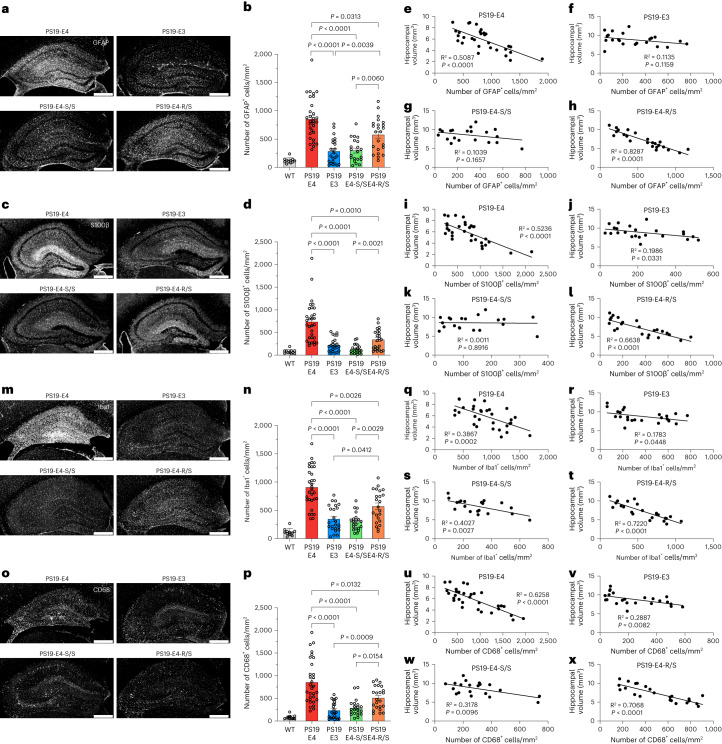
The R136S mutation reduces APOE4-driven gliosis in tauopathy mice. **a**,**b**, Representative images of GFAP immunostaining of astrocytes in the hippocampus of 10-month-old PS19-E4, PS19-E3, PS19-E4-S/S and PS19-E4-R/S mice (**a**) and quantification of the number of GFAP^+^ cells per mm^2^ (**b**) in the hippocampus of these mice and WT mice. **c**,**d**, Representative images of S100β immunostaining of astrocytes in the hippocampus of 10-month-old PS19-E4, PS19-E3, PS19-E4-S/S and PS19-E4-R/S mice (**c**) and quantification of the number of S100β^+^ cells per mm^2^ (**d**) in the hippocampus of these mice and WT mice. **e**–**h**, Correlations between GFAP^+^ cells per mm^2^ and hippocampal volume in PS19-E4 (*n* = 31) (**e**), PS19-E3 (*n* = 23) (**f**), PS19-E4-S/S (*n* = 20) (**g**) and PS19-E4-R/S (*n* = 23) (**h**) mice. **i**–**l**, Correlations between S100β^+^ cells per mm^2^ and hippocampal volume in PS19-E4 (*n* = 31) (**i**), PS19-E3 (*n* = 23) (**j**), PS19-E4-S/S (*n* = 20) (**k**) and PS19-E4-R/S (*n* = 23) (**l**) mice. **m**,**n**, Representative images of Iba1 immunostaining of microglia in the hippocampus of 10-month-old PS19-E4, PS19-E3, PS19-E4-S/S and PS19-E4-R/S mice (**m**) and quantification of the number of Iba1^+^ cells per mm^2^ (**n**) in the hippocampus of these mice and WT mice. **o**,**p**, Representative images of CD68 immunostaining of microglia in the hippocampus of 10-month-old PS19-E4, PS19-E3, PS19-E4-S/S and PS19-E4-R/S mice (**o**) and quantification of the number of CD68^+^ cells per mm^2^ (**p**) in the hippocampus of these mice and WT mice. **q**–**t**, Correlations between Iba1^+^ cells per mm^2^ and hippocampal volume in PS19-E4 (*n* = 31) (**q**), PS19-E3 (*n* = 23) (**r**), PS19-E4-S/S (*n* = 20) (**s**) and PS19-E4-R/S (*n* = 23) (**t**) mice. **u**–**x**, Correlations between CD68^+^ cells per mm^2^ and hippocampal volume in PS19-E4 (*n* = 31) (**u**), PS19-E3 (*n* = 23) (**v**), PS19-E4-S/S (*n* = 20) (**w**) and PS19-E4-R/S (*n* = 23) (**x**) mice. For all quantifications in **b**,**d**,**n**,**p**, WT, *n* = 11; PS19-E4, *n* = 31; PS19-E3, *n* = 24; PS19-E4-S/S, *n* = 21; PS19-E4-R/S, *n* = 23. Scale bars, 500 µm in **a**,**c**,**m**,**o**. Throughout, data are expressed as mean ± s.e.m. In **b**,**d**,**n**,**p**, differences between groups were determined by Welch’s ANOVA followed by Dunnett’s T3 multiple comparison test. Comparisons of *P* ≤ 0.05 are labeled on the graph. Pearson’s correlation analysis (two-sided). Source data

**Extended Data Fig. 5:**
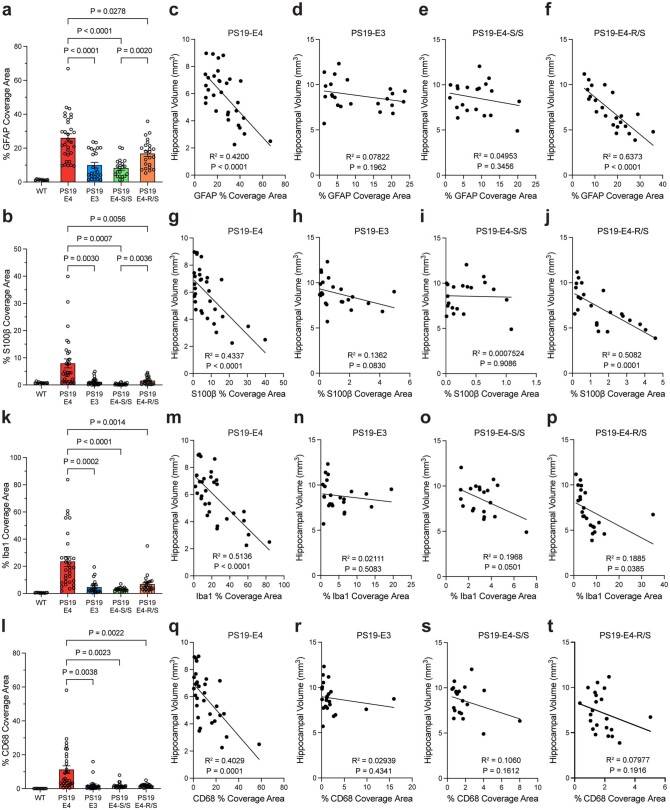
Quantifications and correlations between % coverage area of glial markers and hippocampal volume in tauopathy mice. **a,b**, Quantification of % GFAP (a) or % S100β (b) coverage area in hippocampus of 10-month-old PS19-E4, PS19-E3, PS19-E4-S/S, and PS19-E4-R/S mice. **c–f**, Correlation between % GFAP coverage area and hippocampal volume in PS19-E4 (n = 31) (c), PS19-E3 (n = 23) (d), PS19-E4-S/S (n = 20) (e), and PS19-E4-R/S (n = 23) (f) mice at 10 months of age. **g–j**, Correlation between % S100β coverage area and hippocampal volume in PS19-E4 (n = 31) (g), PS19-E3 (n = 23) (h), PS19-E4-S/S (n = 20) (i), and PS19-E4-R/S (n = 23) (j) mice at 10 months of age. **k,l**, Quantification of % Iba1 (k) or % CD68 (l) coverage area in hippocampus of 10-month-old PS19-E4, PS19-E3, PS19-E4-S/S, and PS19-E4-R/S mice. **m–p**, Correlation between % Iba1 coverage area and hippocampal volume in PS19-E4 (n = 31) (m), PS19-E3 (n = 23) (n), PS19-E4-S/S (n = 20) (o), and PS19-E4-R/S (n = 23) (p) mice at 10 months of age. **q–t**, Correlation between % CD68 coverage area and hippocampal volume in PS19-E4 (n = 31) (q), PS19-E3 (n = 23) (r), PS19-E4-S/S (n = 20) (s), and PS19-E4-R/S (n = 23) (t) mice at 10 months of age. For all quantifications in a,b,k,l, WT, n = 11; PS19-E4, n = 31; PS19-E3, n = 24; PS19-E4-S/S, n = 21; PS19-E4-R/S, n = 23. In a,b,k,l, data are expressed as mean ± s.e.m. and differences between groups were determined by Welch’s ANOVA followed with Dunnett T3 multiple comparison test; comparisons of p ≤ 0.05 were labeled on graph. Pearson’s correlation analysis (two-sided). Source data

We then tested whether the R136S mutation protects against APOE4-driven gliosis in 10-month-old PS19 mice with different APOE genotypes. We quantified astrocytosis with two methods: (1) percent astrocyte coverage area of the hippocampus to account for both glial cell number and morphological size changes and (2) discrete astrocyte number normalized to the hippocampal area to avoid the confounding effect of hippocampal atrophy. PS19-E4 mice displayed the highest hippocampal GFAP coverage area (Extended Data Fig. 5a) and cell numbers (Fig. 5a,b), and both PS19-E3 and PS19-E4-S/S mice had markedly reduced GFAP coverage area (Extended Data Fig. 5a) and cell numbers (Fig. 5a,b) versus PS19-E4 mice. Although PS19-E4-R/S mice showed a significant reduction in GFAP coverage area (Extended Data Fig. 5a) and cell number (Fig. 5a,b) compared to PS19-E4 mice, they still had significantly higher GFAP immunostaining than PS19-E4-S/S mice (Fig. 5a,b and Extended Data Fig. 5a). Similar results were obtained for quantifications of activated astrocyte (S100β^+^) coverage area and cell numbers (Fig. 5c,d and Extended Data Fig. 5b). Notably, a significant correlation was observed between the APOE4-R136S copy number and GFAP^+^ cell number (Supplementary Fig. 5a). Thus, the R136S mutation protects against APOE4-driven astrocytosis in a clear gene dose-dependent manner in this tauopathy model.

PS19 mice at 6 months of age did not show the same differences in astrocytosis across APOE genotype groups, although PS19-E4-S/S mice had a trend toward lower astrocytosis as compared to PS19-E4 mice (Extended Data Fig. 2d,g,h). This suggests that the protection of R136S mutation against APOE4-driven astrocytosis in the context of tauopathy starts at about or after 6 months of age.

Notably, both GFAP and S100β coverage areas (Extended Data Fig. 5c,g) and cell numbers (Fig. 5e,i) inversely correlated with hippocampal volume in 10-month-old PS19-E4 mice, suggesting the contribution of astrocytosis to hippocampal degeneration, as reported previously^7^. This correlation was mostly eliminated in PS19-E3 mice (Fig. 5f,j and Extended Data Fig. 5d,h) and PS19-E4-S/S mice (Fig. 5g,k and Extended Data Fig. 5e,i), suggesting that milder astrocytosis in these mice might not contribute to hippocampal degeneration. However, this relationship was preserved in PS19-E4-R/S mice (Fig. 5h,l and Extended Data Fig. 5f,j), suggesting that moderate astrocytosis is sufficient to contribute to hippocampal degeneration.

Although mild Tau pathology in PS19-E3 and PS19-E4-S/S mice may not contribute to hippocampal degeneration (Fig. 4g,h), it positively correlated with astrocytosis (Supplementary Fig. 6e,f,i,j). This suggests that mild Tau pathology can induce mild astrocytosis, although the latter may not be sufficient to contribute to hippocampal degeneration (Fig. 5f,g,j,k). In PS19-E4-R/S mice, in which severe Tau pathology (Fig. 1c,e,f) and moderate astrocytosis (Fig. 5b,d) occurred, the Tau pathology positively, albeit weakly, correlated with astrocytosis (Supplementary Fig. 6m,n). Thus, in the presence of severe Tau pathology and moderate astrocytosis, both pathologies contribute to hippocampal degeneration (Figs. 4i and 5h,l). In PS19-E4 mice, in which both severe Tau pathology (Fig. 1c,e,f) and severe astrocytosis (Fig. 5b,d) occurred, Tau pathology had a trending correlation with astrocytosis (Supplementary Fig. 6a,b). These findings suggest that the astrocytosis might reach a plateau in PS19-E4 mice. Notably, in the presence of both severe Tau pathology and severe astrocytosis, both pathologies contribute to hippocampal degeneration (Figs. 4f and 5e,i and Extended Data Fig. 5c,g).

### E4-S/S reduces microgliosis in tauopathy mice

We next surveyed hippocampal microgliosis via immunostaining of Iba1 (Fig. 5m), a marker for total microglial population, and CD68 (Fig. 5o), a marker of activated microglia, in 10-month-old PS19 mice with different APOE genotypes. The PS19-E4 mice exhibited significantly higher microglia immunoreactivity (both coverage area and cell number) via measurements of both markers than the PS19-E3, PS19-E4-S/S and PS19-E4-R/S mice (Fig. 5m,n,o,p and Extended Data Fig. 5k,l). The PS19-E4-R/S mice showed significantly higher Iba1^+^ and CD68^+^ cell numbers than PS19-E4-S/S mice (Fig. 5n,p), suggesting an intermediate level of protection against microgliosis. This was confirmed by significant correlation of the APOE4-R136S copy number to Iba1^+^ cell number (Supplementary Fig. 5b). Together, these data indicate that the R136S mutation protects against APOE4-promoted microgliosis in the context of tauopathy in a gene dose-dependent manner.

In the 6-month-old PS19 mice, we did not observe the same differences in microgliosis across APOE genotype groups. The PS19-E4-S/S mice did show a trend toward lower microgliosis as compared to PS19-E4 mice (Extended Data Fig. 2d,i,j), suggesting that the E4-S/S protection against microgliosis may start at about or after 6 months of age.

Similar to findings of astrocytosis, both Iba1 and CD68 coverage areas (Extended Data Fig. 5m,q) and cell numbers (Fig. 5q,u) inversely correlated with hippocampal volume in 10-month-old PS19-E4 and PS19-E4-R/S mice (Fig. 5t,x and Extended Data Fig. 5p,t), suggesting the contribution of microgliosis to hippocampal degeneration, as reported in previous studies^7,55^. Unlike the data in astrocytosis, the relationship between microgliosis and hippocampal degeneration in PS19-E3 and PS19-E4 mice was somewhat preserved (Fig. 5r,s,v,w).

Similar to findings of astrocytosis, mild Tau pathology in PS19-E3 and PS19-E4-S/S mice may not contribute to hippocampal degeneration (Fig. 4g,h) yet positively correlates with microgliosis (Supplementary Fig. 6g,h,k,l). This suggests that mild Tau pathology can induce mild microgliosis. Interestingly, mild microgliosis negatively correlated with hippocampal volume in PS19-E3 and PS19-E4-S/S mice (Fig. 5r,s,v,w), suggesting that mild microgliosis is likely more toxic than mild astrocytosis and sufficient to cause hippocampal degeneration (Fig. 5f,g,k). In PS19-E4-R/S mice, in which severe Tau pathology (Fig. 1c,e,f) and moderate microgliosis (Fig. 5n,p) occurred, Tau pathology positively correlated with microgliosis (Supplementary Fig. 6o,p). Thus, in the presence of severe Tau pathology and moderate microgliosis, both pathologies contribute to hippocampal degeneration (Figs. 4i and 5t,x). Interestingly, in PS19-E4 mice, in which both severe Tau pathology (Fig. 1c,e,f) and severe microgliosis (Fig. 5n,p) occurred, Tau pathology significantly correlated with only CD68^+^ (active) microgliosis (Supplementary Fig. 6d) but not Iba1^+^ (general) microgliosis (Supplementary Fig. 6c). These data indicate that the general microgliosis might reach a plateau in PS19-E4 mice. Notably, in the presence of both severe Tau pathology and severe microgliosis, both pathologies contribute to hippocampal degeneration (Figs. 4f and 5q,u and Extended Data Fig. 5m,q).

In all, these findings indicate that the homozygous and, to a lesser extent, heterozygous R136S mutation protects against APOE4-driven astrocytosis and microgliosis in the context of tauopathy in an age-dependent manner, which can lead to reduced hippocampal neurodegeneration and atrophy.

### E4-S/S rescues transcriptomic changes in neurons and oligodendrocytes

**Fig. 6:**
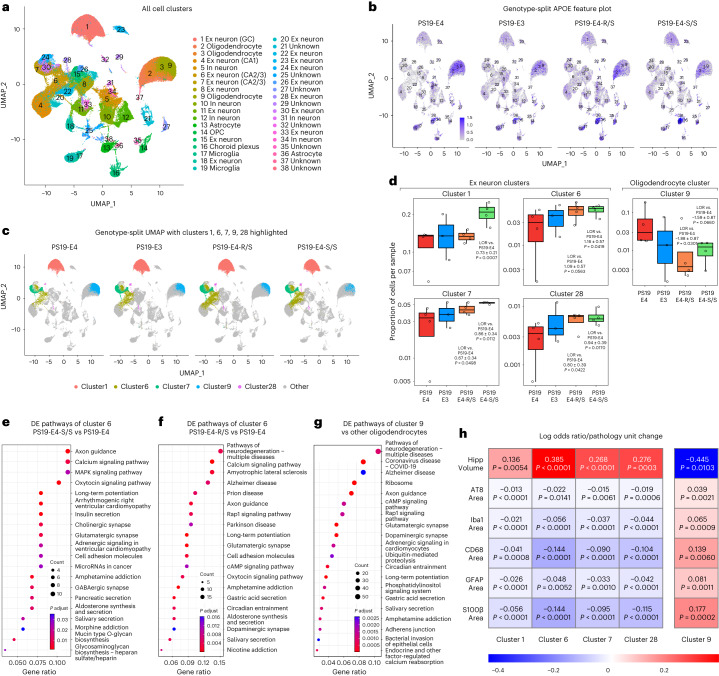
snRNA-seq reveals protective effects of the R136S mutation on APOE4-driven neuronal and oligodendrocytic deficits in mice. **a**, UMAP projection of 38 distinct cell clusters in hippocampi of 10-month-old PS19-E4 (*n* = 4), PS19-E3 (*n* = 3), PS19-E4-S/S (*n* = 4) and PS19-E4-R/S (*n* = 4) mice. **b**, Feature plot showing relative levels of normalized human APOE gene expression across all 38 hippocampal cell clusters by APOE genotype (PS19-E4, *n* = 4; PS19-E3, *n* = 3; PS19-E4-S/S, *n* = 4; PS19-E4-R/S, *n* = 4; *n* = mice). **c**, UMAP projection highlighting hippocampal cell clusters 1, 6, 7, 9 and 28 for each genotype group. **d**, Box plot of the proportion of cells from each sample in clusters 1, 6, 7, 9 and 28 in PS19-E4 (*n* = 4), PS19-E3 (*n* = 3), PS19-E4-R/S (*n* = 4), and PS19-E4-S/S (*n* = 4) mice. The lower, middle and upper hinges of the box plots correspond to the 25th, 50th and 75th percentiles, respectively. The upper whisker of the box plot extends from the upper hinge to the largest value no further than 1.5 Å~ IQR from the upper hinge. IQR, interquartile range, or distance between the 25th and 75th percentiles. The lower whisker extends from the lower hinge to the smallest value at most 1.5 Å~ IQR from the lower hinge. The LORs are the mean ± s.e.m. estimates of LOR for these clusters, which represents the change in the log odds of cells per sample from PS19-E3, PS19-E4-R/S or PS19-E4-S/S mice belonging to the respective clusters compared to the log odds of cells per sample from PS19-E4 mice. **e**,**f**, KEGG pathway enrichment dot plot of top 20 pathways significantly enriched for DE genes of neuronal cluster 6 in PS19-E4-S/S (**e**) or PS19-E4-R/S (**f**) versus PS19-E4 mice. *P* values are based on a two-sided hypergeometric test and are adjusted for multiple testing using the Benjamini–Hochberg method. Gene ratio represents the proportion of genes in the respective gene set that are deemed to be DE using the two-sided Wilcoxon rank-sum test as implemented in the FindMarkers function in Seurat. **g**, KEGG pathway enrichment dot plot of top 20 pathways significantly enriched for DE genes of oligodendrocyte cluster 9 versus oligodendrocyte cluster 2. **h**, Heat map plot of LOR per unit change in each pathological measurement for clusters 1, 6, 7, 9 and 28. The LOR represents the mean estimate of the change in the log odds of cells per sample from a given animal model, corresponding to a unit change in a given histopathological parameter. Associations with pathologies are colored (negative associations, blue; positive associations, red). *P* values in **d** are from fits to a GLMM_AM, and *P* values in **h** are from fits to a GLMM_histopathology; the associated tests are two-sided. All error bars represent s.e.m. Ex neuron, excitatory neuron; In neuron, inhibitory neuron.

**Extended Data Fig. 6:**
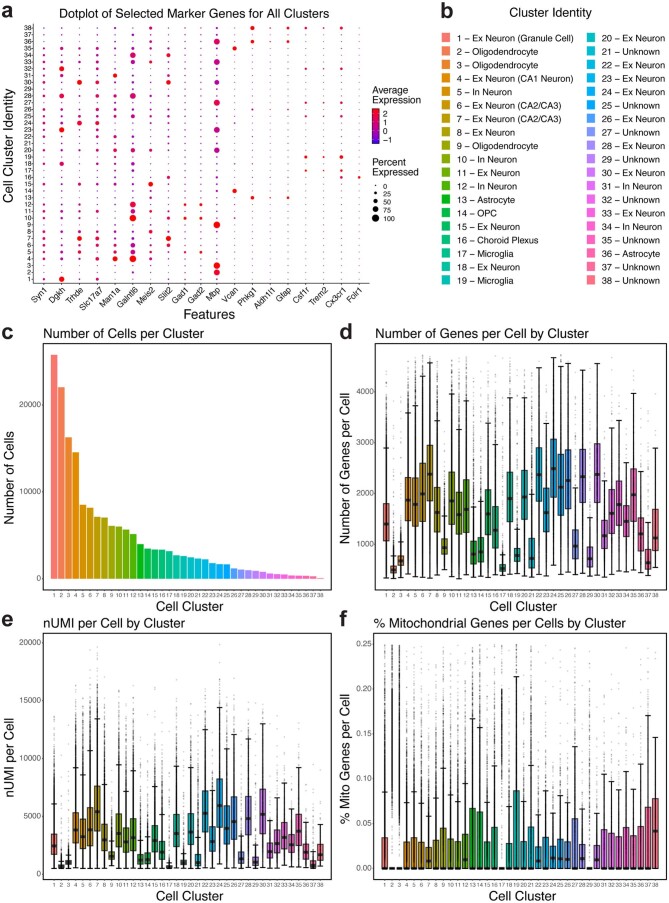
Quality control measures in snRNA-seq analysis of tauopathy mice with different APOE genotypes. **a**, Dot-plot depicting normalized average expression of selected cell identity marker genes for all 38 distinct hippocampal cell clusters from mice at 10 months of age. **b**, Cluster identity of 38 distinct cell types. **c**, The number of cells per cluster. **d**, The number of genes per cell in each cluster (± s.e.m). **e**, The number of nUMI (unique molecular identified) per cell in each cluster (± s.e.m). **f**, The % mitochondrial genes per cell in each cluster (± s.e.m). Number of cells (n) for each cell cluster can be found in c. For details of the analyses, see Methods.

We next evaluated whether the R136S mutation affects the transcriptomic signature of the hippocampus at a cell-type-specific level. We performed snRNA-seq on isolated hippocampi from 10-month-old PS19-E4, PS19-E3, PS19-E4-S/S and PS19-E4-R/S mice. The dataset contained 175,080 nuclei covering 26,422 genes after normalization and filtering (Extended Data Fig. 6). Unsupervised clustering by the Louvain algorithm^60^ and visualization by uniform manifold approximation and projection (UMAP) produced 38 distinct cell clusters (Fig. 6a). Based on canonical marker gene expression, we identified 16 excitatory neuron clusters (1, 4, 6–8, 11, 15, 18, 20, 22–24, 26, 28, 30 and 33), five inhibitory neuron clusters (5, 10, 12, 31 and 34) and 17 non-neuronal clusters, including three oligodendrocyte clusters (2, 3 and 9), two astrocyte clusters (13 and 36), two microglia clusters (17 and 19) and one oligodendrocyte progenitor cell (OPC) cluster (14) (Fig. 6a, Extended Data Fig. 6a,b and Supplementary Table 1). As expected, APOE was highly expressed in astrocytes (13 and 36) and, to a lesser extent, in microglia (17 and 19) in all groups of mice (Fig. 6b and Extended Data Fig. 8a,e). Some neurons and oligodendrocytes also express APOE (Fig. 6b), as we previously reported^22,55^. No significant difference was observed in APOE expression in each cell cluster across various APOE genotypes (Supplementary Fig. 7).

To further examine cell-type-specific alterations in PS19-E3, PS19-E4-S/S and PS19-E4-R/S mice versus PS19-E4 mice, we used log odds ratio (LOR) estimates from a generalized linear mixed-effects model to assess association with animal models (GLMM_AM). We discovered clusters in which there was a significant change in the likelihood that the cluster contained more or fewer cells from PS19-E4-S/S or PS19-E4-R/S mice than from PS19-E4 mice. For example, excitatory neuron cluster 1 (GC) and cluster 6 (CA2/3) had significantly higher odds of containing cells from PS19-E4-S/S mice than from PS19-E4 mice (Fig. 6c,d and Supplementary Table 1), suggesting that E4-S/S promotes the survival of, or protects against the loss of, these neuronal subpopulations. Excitatory neuron clusters 7 (CA2/3) and 28 had significantly higher odds of containing cells from either PS19-E4-S/S or PS19-E4-R/S mice than from PS19-E4 mice (Fig. 6c,d and Supplementary Table 1), suggesting that both E4-S/S and E4-R/S promote the survival of, or protect against the loss of, these neuronal subpopulations.

**Extended Data Fig. 7:**
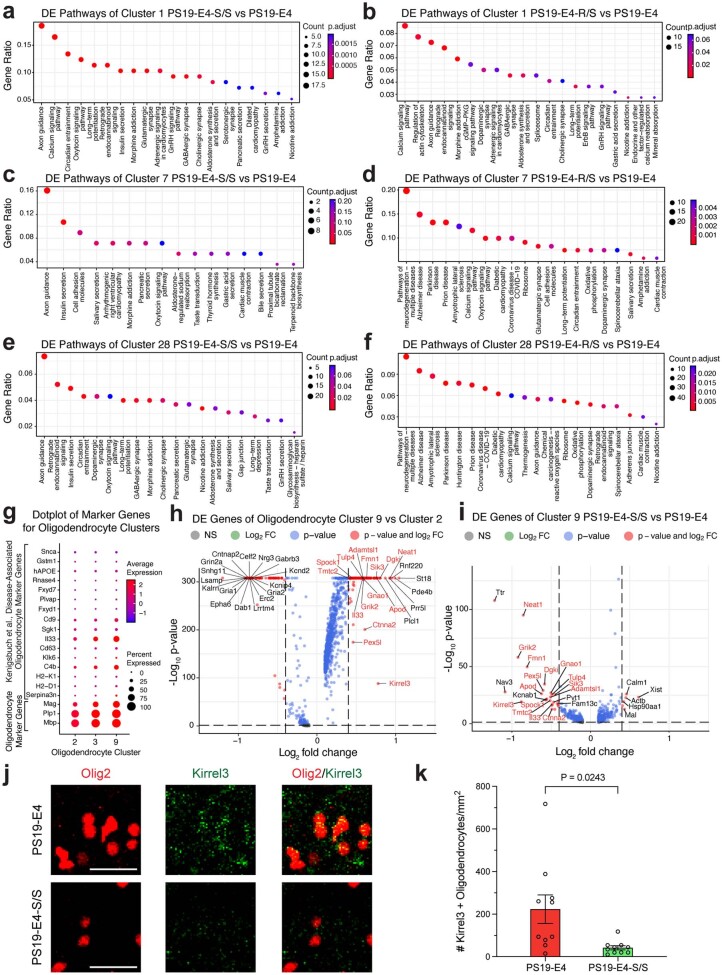
snRNA-seq analysis of disease-protective neuronal clusters and disease-associated oligodendrocyte cluster. **a,b**, KEGG pathway enrichment dot-plot of top 20 pathways significantly enriched for DE genes of neuronal cluster 1 in PS19-E4-S/S (a) or PS19-E4-R/S (b) versus PS19-E4 mice at 10 months of age. **c,d**, KEGG pathway enrichment dot-plot of top 20 pathways significantly enriched for DE genes of neuronal cluster 7 in PS19-E4-S/S (c) or PS19-E4-R/S (d) versus PS19-E4 mice at 10 months of age. **e,f**, KEGG pathway enrichment dot-plot of top 20 pathways significantly enriched for DE genes of neuronal cluster 28 in PS19-E4-S/S (e) or PS19-E4-R/S (f) versus PS19-E4 mice at 10 months of age. **g**, Dot-plot normalized average expression of selected homeostatic and disease-associated oligodendrocyte (DAO) marker genes for oligodendrocyte clusters 2, 3, and 9. **h**, Volcano plot for top DE genes of oligodendrocyte cluster 9 versus cluster 2. **i**, Volcano plot for top DE genes of oligodendrocyte cluster 9 in PS19-E4-S/S versus PS19-E4 mice at 10 months of age. **j**, Representative images of Kirrel3^+^ Olig2^+^ oligodendrocytes in the hippocampus of 10-month-old PS19-E4 and PS19-E4-S/S mice at 10 months of age. Scale bars are 25 µm. **k**, Quantification of the number of Kirrel3^+^ Olig2^+^ oligodendrocytes (per mm^2^) within the dentate gyrus of hippocampus in 10-month-old PS19-E4 (n = 10) and PS19-E4-S/S (n = 10) mice at 10 months of age. In a–f, P-values are based on a two-sided hypergeometric test and are adjusted for multiple testing using the Benjamini-Hochberg method. Gene ratio represents the proportion of genes in the respective gene set that are deemed to be differentially expressed using the two-sided Wilcoxon Rank-Sum test as implemented in the FindMarkers function in Seurat. In h,i, horizonal dashed line indicates p-value = 0.05 and vertical dashed lines indicate log_2_ fold change = 0.4. All error bars represent s.e.m. Difference between groups in k was determined by unpaired, two-sided Welch’s t-test. DE, differentially expressed; NS, nonsignificant; FC, fold change. For details of the analyses, see Methods. Source data

Analyses of differentially expressed (DE) genes and their enriched Kyoto Encyclopedia of Genes and Genomes (KEGG) pathways in PS19-E4-S/S or PS19-E4-R/S mice versus PS19-E4 mice revealed that the top enriched DE pathways in these clusters were indicative of neuronal health and function, including axon guidance, synaptic integrity and long-term potentiation (Fig. 6e,f for cluster 6; Extended Data Fig. 7a–f for clusters 1, 7 and 28; and Supplementary Table 1 for clusters 1, 6, 7 and 28). These suggest that even heterozygous R136S mutation provides protection against APOE4 effects in these clusters at the transcriptomic level. Notably, some DE pathways did differ based on the R136S gene dose. For example, some neurodegenerative disease-related pathways appeared only in the comparison of PS19-E4-R/S versus PS19-E4 mice, and heparin sulfate biosynthesis pathways appeared only in the comparison of PS19-E4-S/S versus PS19-E4 mice (Fig. 6e,f, Extended Data Fig. 7c–f and Supplementary Table 1). This suggests gene dose-related differential effects of APOE4-R136S mutation on these pathways in some neuronal clusters.

The oligodendrocyte cluster 9 had lower odds of containing cells from PS19-E4-S/S and PS19-E4-R/S mice than from PS19-E4 mice (Fig. 6c,d, and Supplementary Table 1). Further analyses comparing oligodendrocyte cluster 9 versus cluster 2 revealed enrichment of DE genes and KEGG pathways related to neurodegeneration, including AD (Fig. 6g and Supplementary Table 1), suggesting that cells in cluster 9 are disease-associated oligodendrocytes (DAOs). Some DE genes recently identified as markers of DAOs in AD mouse models^61^ were also upregulated in cluster 9 versus cluster 2, such as those mediating the inflammatory process (*H2-D1*, *Il33* and *C4b*) (Extended Data Fig. 7g). Additionally, the DAO cluster 9 had many significantly upregulated genes uniquely identified in the current study, including *Kirrel3*, *Neat1*, *Apod*, *Dgki*, *Fmn1*, *Pex5l*, *Sik3* and *Grik2* (Extended Data Fig. 7h). Notably, many of these highly upregulated DAO genes unique to cluster 9 were markedly downregulated in PS19-E4-S/S mice versus PS19-E4 mice (Extended Data Fig. 7h,i). These data indicate not only that the DAO cluster 9 was diminished by the APOE4-R136S mutation but also that the R136S mutation markedly reverses the distinct transcriptomic profile of the DAOs. Using Kirrel3 as a marker, immunofluorescent staining confirmed a significant decrease in the DAO cluster 9 in the hippocampus of PS19-E4-S/S mice versus PS19-E4 mice (Extended Data Fig. 7j,k).

LOR estimates from another GLMM to assess associations with histopathology (GLMM_histopathology) uncovered that the proportion of cells in excitatory neuron clusters 1, 6, 7 and 28 exhibited significant positive associations with hippocampal volume and negative associations with the coverage areas of p-Tau and gliosis (Fig. 6h and Supplementary Table 2). Conversely, the proportion of DAOs in oligodendrocyte cluster 9 had significant negative association with hippocampal volume and positive association with the coverage areas of p-Tau and gliosis (Fig. 6h and Supplementary Table 2). Thus, the APOE4-R136S mutation promoted the survival of excitatory neuronal subpopulations (clusters 1, 6, 7 and 28) and the elimination of DAO subpopulation (cluster 9).

### E4-S/S increases disease-protective and decreases disease-associated astrocytes

**Fig. 7:**
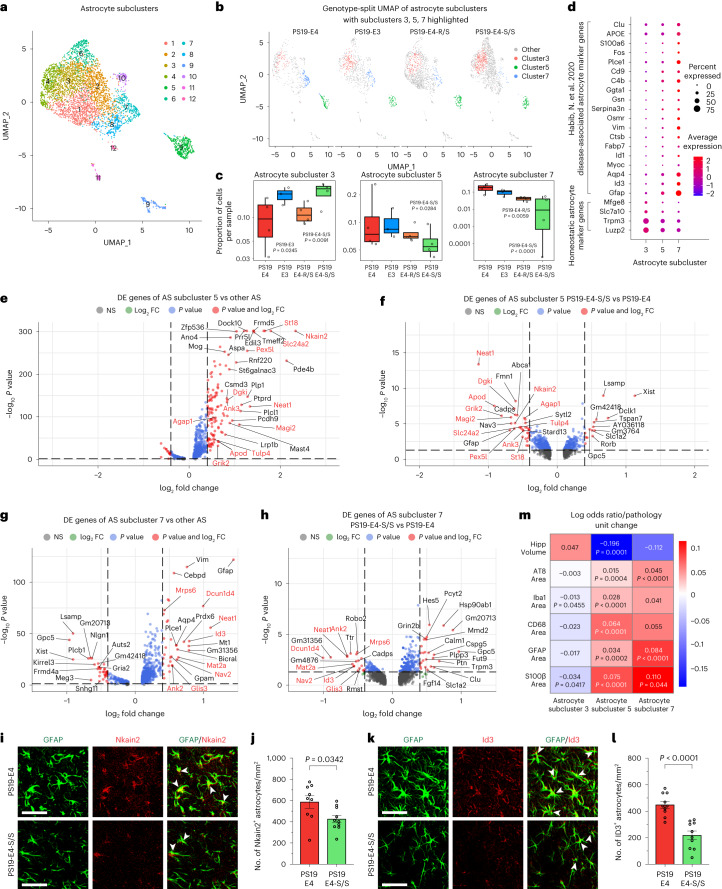
The APOE4-R136S mutation increases disease-protective and decreases disease-associated astrocyte subpopulations. **a**, UMAP projection of 12 astrocyte subclusters after subclustering hippocampal cell clusters 13 and 36 (Fig. 6a) from 10-month-old mice with different APOE genotypes. **b**, UMAP projection highlighting astrocyte subclusters 3, 5 and 7 for each genotype group (PS19-E4, *n* = 4; PS19-E3, *n* = 3; PS19-E4-S/S, *n* = 4; PS19-E4-R/S, *n* = 4; *n* = mice). **c**, Box plot of the proportion of cells from each sample in astrocyte subclusters 3, 5 and 7 in PS19-E4 (*n* = 4), PS19-E3 (*n* = 3), PS19-E4-R/S (*n* = 4), and PS19-E4-S/S (*n* = 4) mice. The lower, middle and upper hinges of the box plots correspond to the 25th, 50th and 75th percentiles, respectively (see Fig. 6d for details). The LORs are the mean ± s.e.m. estimates of LOR for these clusters, which represents the change in the log odds of cells per sample from PS19-E3, PS19-E4-R/S or PS19-E4-S/S mice belonging to the respective clusters compared to the log odds of cells per sample from PS19-E4 mice. LOR versus PS19-E4 for subcluster 3: PS19-E3, 0.67 ± 0.30; PS19-E4-S/S, 0.71 ± 0.27; subcluster 5: PS19-E4-S/S, −0.74 ± 0.34; subcluster 7: PS19-E4-R/S, −1.57 ± 0.57; PS19-E4-S/S, −2.75 ± 0.62. **d**, Dot plot of normalized average expression of selected homeostatic and DAA marker genes for astrocyte subclusters 3, 5 and 7. **e**, Volcano plot for top 30 DE genes of astrocyte subcluster 5 versus other astrocyte subclusters. **f**, Volcano plot for top 30 DE genes of astrocyte subcluster 5 in PS19-E4-S/S versus PS19-E4 mice. **g**, Volcano plot for top 30 DE genes of astrocyte subcluster 7 versus other astrocyte subclusters. **h**, Volcano plot for top 30 DE genes of astrocyte subcluster 7 in PS19-E4-S/S versus PS19-E4 mice. **i**, Representative images of Nkain2^+^GFAP^+^ astrocytes in the hippocampus of 10-month-old PS19-E4 (*n* = 9) and PS19-E4-S/S (*n* = 10) mice. **j**, Quantification of the number of Nkain2^+^GFAP^+^ cells (per mm^2^) within the molecular layer of hippocampus. **k**, Representative images of Id3^+^GFAP^+^ astrocytes in the hippocampus of 10-month-old PS19-E4 (*n* = 10) and PS19-E4-S/S (*n* = 10) mice. **l**, Quantification of the number of Id3^+^GFAP^+^ cells (per mm^2^) within the molecular layer of hippocampus. **m**, Heat map plot of LOR per unit change in each pathological measurement for astrocyte subclusters 3, 5 and 7. *P* values in **c** are from fits to a GLMM_AM, and *P* values in **m** are from fits to a GLMM_histopathology; the associated tests are two-sided. In **e**–**h**, horizonal dashed line indicates *P* = 0.05, and vertical dashed lines indicate log_2_ fold change = 0.4. The unadjusted *P* values and log_2_ fold change values used were generated from the gene set enrichment analysis using the two-sided Wilcoxon rank-sum test as implemented in the FindMarkers function of the Seurat package. Gene names highlighted in red text indicate that they are selected marker genes for DAAs. Scale bars in **i** and **k**, 50 µm. All error bars represent s.e.m. Differences between groups in **j** and **l** were determined by unpaired, two-sided Welch’s *t*-test. AS, astrocyte; NS, not significant; FC, fold change. Source data

**Extended Data Fig. 8:**
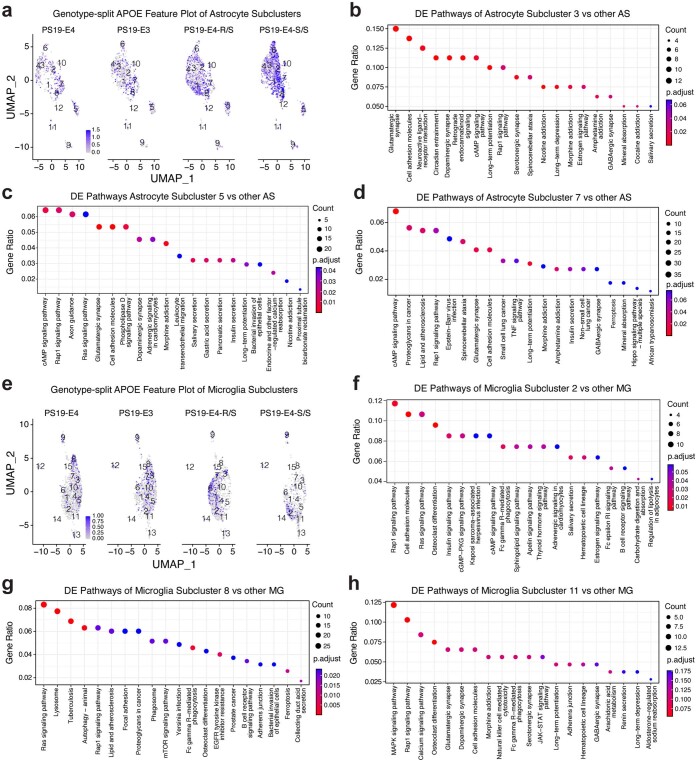
snRNA-seq analysis of astrocyte and microglia subclusters. **a**, Feature plot showing relative levels of normalized human APOE gene expression across all 12 astrocyte subclusters by APOE genotype from mice at 10 months of age. **b–d**, KEGG pathway enrichment dot-plot of top 20 pathways significantly enriched for DE genes of astrocyte subclusters 3 (b), 5 (c), and 7 (d) versus other astrocyte subclusters. **e**, Feature plot showing relative levels of normalized human APOE gene expression across all 15 microglia subclusters by APOE genotype from mice at 10 months of age. **f–h**, KEGG pathway enrichment dot-plot of top 20 pathways significantly enriched for DE genes of microglia subclusters 2 (f), 8 (g), and 11 (h) versus other microglia subclusters. In b–d and f–h, P-values are based on a two-sided hypergeometric test and are adjusted for multiple testing using the Benjamini-Hochberg method. Gene Ratio represents the proportion of genes in the respective gene set that are deemed to be differentially expressed using the two-sided Wilcoxon Rank-Sum test as implemented in the FindMarkers function in Seurat.

Based on our observation that even E4-R/S significantly reduces gliosis (Fig. 5 and Extended Data Fig. 5), we further dissected the effects of the APOE4-R136S mutation on subtypes of astrocytes and microglia. Subclustering of astrocytes (clusters 13 and 36 in Fig. 6a) produced 12 astrocyte subpopulations (Fig. 7a). LOR estimates from a GLMM_AM showed that astrocyte subcluster 3 was enriched in PS19-E3 and PS19-E4-S/S mice, but not in PS19-E4-R/S mice, compared to PS19-E4 mice (Fig. 7b,c and Supplementary Table 3). LOR estimates also revealed that astrocyte subclusters 5 and 7 had lower odds of containing cells from PS19-E4-S/S mice than from PS19-E4 mice (Fig. 7b,c and Supplementary Table 3). In fact, subcluster 7 was almost completely eliminated in PS19-E4-S/S mice (Fig. 7b). PS19-E4-R/S mice also had a significant decrease in LOR of astrocyte subcluster 7, although to a lesser extent than PS19-E4-S/S mice (Fig. 7b,c). All of these three astrocyte subclusters (3, 5 and 7) expressed high levels of APOE (Extended Data Fig. 8a). DE gene analyses comparing each of these subclusters to all other astrocyte subclusters identified astrocyte subcluster 3 as homeostatic astrocytes and astrocyte subclusters 5 and 7 as disease-associated astrocytes (DAAs), with upregulation of *Gfap*, *Aqp4*, *Ctsb*, *Vim*, *Serpina3a*, *C4b* and *Cd9* expression (Fig. 7d and Supplementary Table 3), similar to those reported previously^62^. Additionally, astrocyte subclusters 5 and 7 also had many highly upregulated DE genes uniquely identified in the current study for subcluster 5 (*Neat1*, *Pex5l1*, *Nkain2*, *Dgki*, *Apod* and *Ank3*) and subcluster 7 (*Neat1*, *Ank3*, *Mat2a*, *Nav2*, *Glis3* and *Mrps6)* (Fig. 7e,g and Supplementary Table 3), as compared to other astrocyte subclusters. Many of these highly upregulated DE genes in DAA subclusters 5 and 7 were markedly downregulated in PS19-E4-S/S mice versus PS19-E4 mice (Fig. 7f,h and Supplementary Table 3). Using Nkain2 as a marker for DAA subcluster 5 and Id3 as a marker for DAA subcluster 7, immunofluorescent staining confirmed a significant decrease in both DAA clusters in the hippocampus of PS19-E4-S/S mice versus PS19-E4 mice (Fig. 7i–l).

**Extended Data Fig. 9:**
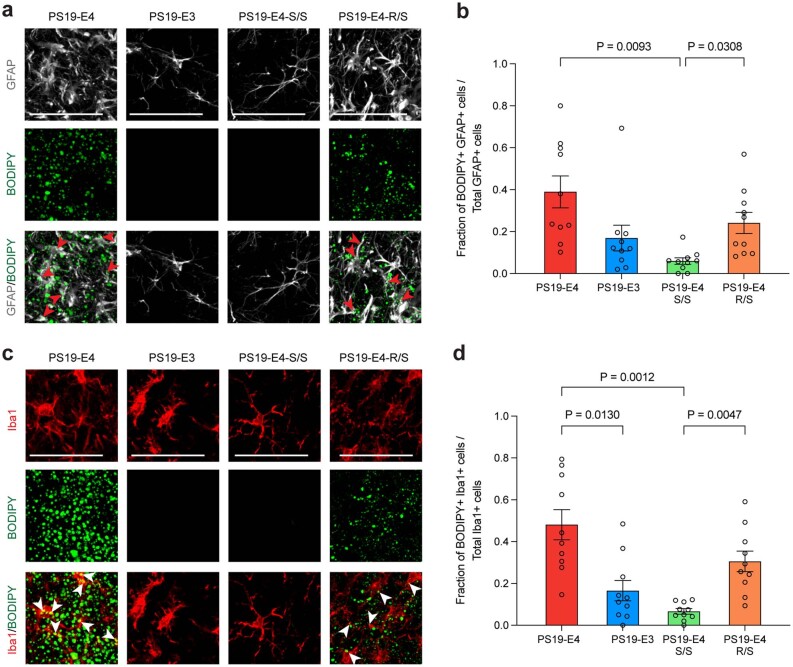
The R136S mutation reduces APOE4-induced neutral lipid accumulation in astrocytes and microglia in tauopathy mice. **a**, Representative images of neutral lipid visualized with BODIPY staining in GFAP^+^ astrocytes in the dentate gyrus of hippocampus in PS19-E4, PS19-E3, PS19-E4-S/S, and PS19-E4-R/S mice at 10 months of age (example overlap shown in red arrows). **b**, Quantification of the fraction of BODIPY^+^ GFAP^+^ cells per total GFAP^+^ cells in the dentate gyrus of hippocampus in PS19-E4, PS19-E3, PS19-E4-S/S, and PS19-E4-R/S mice at 10 months of age. **c**, Representative images of neutral lipid visualized with BODIPY staining in Iba1^+^ microglia in the dentate gyrus of hippocampus in PS19-E4, PS19-E3, PS19-E4-S/S, and PS19-E4-R/S mice at 10 months of age (example overlap shown in white arrows). **d**, Quantification of the fraction of BODIPY^+^ Iba1^+^ cells per total Iba1^+^ cells in the dentate gyrus of hippocampus in PS19-E4, PS19-E3, PS19-E4-S/S, and PS19-E4-R/S mice at 10 months of age. In a and c, scale bars are 50 µm. In b and d, PS19-E4, n = 10; PS19-E3, n = 10; PS19-E4-S/S, n = 10; PS19-E4-R/S, n = 10; n=mice. In b,d, data are expressed as mean ± s.e.m. and differences between groups were determined by Welch’s ANOVA followed with Dunnett T3 multiple comparison test; comparisons of p ≤ 0.05 were labeled on graph. DE, differentially expressed; AS, astrocyte; MG, microglia. For details of the analyses, see Methods. Source data

DE pathway analyses supported the notion that subcluster 3 comprised homeostatic astrocytes and subclusters 5 and 7 comprised DAAs (Extended Data Fig. 8b–d and Supplementary Table 3). Interestingly, DAA subcluster 7 had enrichment of lipid and atherosclerosis pathway genes (Extended Data Fig. 8d and Supplementary Table 3), and GFAP/BODIPY double staining revealed neutral lipid accumulation in astrocytes in PS19-E4 mice (Extended Data Fig. 9a,b). Strikingly, the R136S mutation eliminated neutral lipid accumulation in astrocytes in PS19-E4-S/S mice (Extended Data Fig. 9a,b), suggesting that the R136S mutation protects against APOE4-induced dysregulation of this process in astrocytes. Additionally, astrocytes in PS19-E4 mice had enlarged Lamp1^+^ lysosomes as compared to those in PS19-E3 mice, and the APOE4-R136S mutation reduced lysosome size to levels similar to that of the PS19-E3 mice (Supplementary Fig. 8a,b).

The enrichment of cells in the homeostatic astrocyte subcluster 3 was positively associated with hippocampal volume and negatively associated with the coverage areas of p-Tau and gliosis (Fig. 7m and Supplementary Table 4), as determined by LOR estimates from GLMM_histopathology. Likewise, the LOR estimates also revealed that enrichments of DAA subclusters 5 and 7 were negatively associated with hippocampal volume and positively associated with the coverage areas of p-Tau and gliosis (Fig. 7m and Supplementary Table 4). Together, these findings illustrate that E4-S/S increases disease-protective homeostatic astrocytes (subcluster 3) and both E4-S/S and E4-R/S eliminate DAAs (subclusters 5 and 7) in the hippocampus of the tauopathy mice, with E4-S/S having a greater effect than E4-R/S.

**Extended Data Fig. 10:**
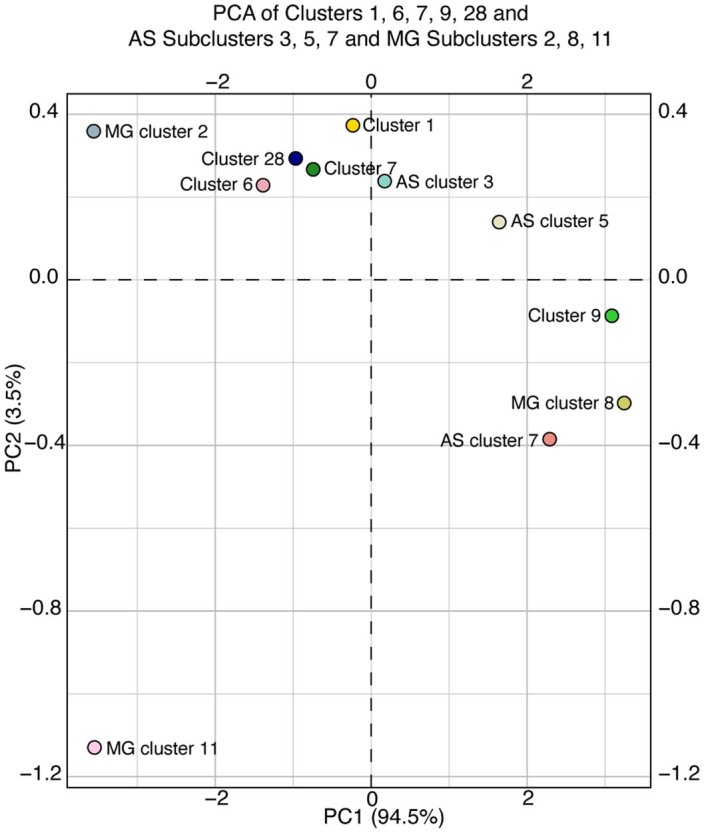
PCA clustering of selected hippocampal cell clusters as well as astrocyte and microglia subclusters against all measured pathologies. Principal component analysis plot for hippocampal clusters 1, 6, 7, 9, 28, astrocyte subclusters 3, 5, 7, and microglia subclusters 2, 8, 11 against all measured pathologies (hippocampal volume and coverage areas of AT8, Iba1, CD68, GFAP, and S100β). PC1 and PC2 showed the overall relationship between clusters based on similarity of the estimated log odds ratio per unit change in six pathologies per cluster/subcluster. AS, astrocyte; MG, microglia. For details of the analyses, see Methods.

To further assess the relationship between the disease-protective and disease-associated astrocyte subpopulations, we applied principal component analysis (PCA) clustering of neuronal clusters 1, 6, 7 and 28 and oligodendrocyte cluster 9 together with astrocyte subclusters 3, 5 and 7 against all measured pathologies. Such PCA analysis revealed that the disease-protective astrocyte subcluster 3 had a similar contribution to the measured pathologies as the four disease-protective neuronal clusters (1, 6, 7 and 28) (Extended Data Fig. 10). Likewise, DAA subcluster 7 had a similar contribution to the measured pathologies as the DAOs (cluster 9) (Extended Data Fig. 10). These findings support the notion that APOE4-R136S leads to a significant increase in the disease-protective homeostatic astrocyte subpopulation, likely contributing to the enrichment of neuronal clusters 1, 6, 7 and 28, as well as to a significant reduction in the DAA subpopulation, likely contributing to the elimination of the DAOs.

### E4-S/S increases disease-protective and decreases disease-associated microglia

**Fig. 8:**
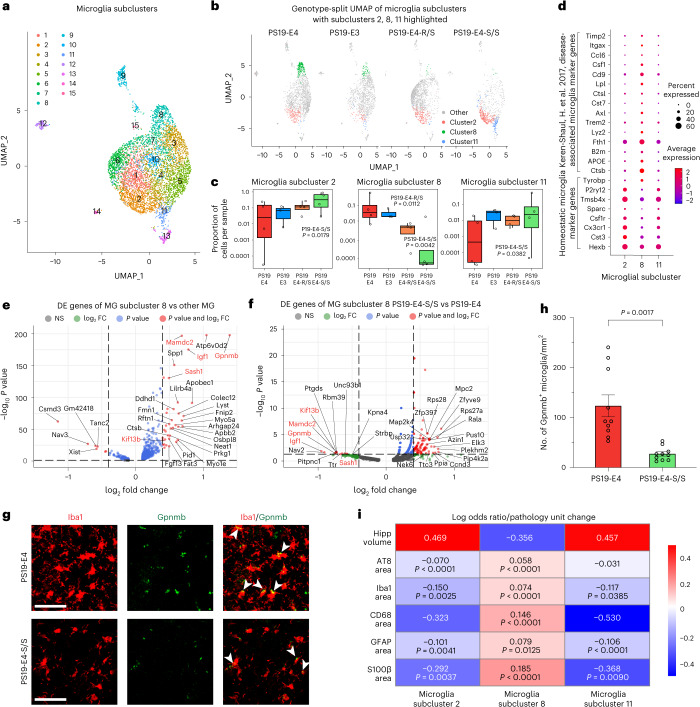
The APOE4-R136S mutation increases disease-protective and decreases disease-associated microglial subpopulations. **a**, UMAP projection of 15 microglia subclusters after subclustering hippocampal cell clusters 17 and 19 (Fig. 6a) from 10-month-old mice with different APOE genotypes. **b**, UMAP projection highlighting microglia subclusters 2, 8 and 11 for each mouse genotype group (PS19-E4, *n* = 4; PS19-E3, *n* = 3; PS19-E4-S/S, *n* = 4; PS19-E4-R/S, *n* = 4; *n* = mice). **c**, Box plot of the proportion of cells from each sample in microglia subclusters 2, 8, and 11 in PS19-E4 (*n* = 4), PS19-E3 (*n* = 3), PS19-E4-R/S (*n* = 4), and PS19-E4-S/S (*n* = 4) mice. The lower, middle and upper hinges of the box plots correspond to the 25th, 50th and 75th percentiles, respectively (see Fig. 6d for details). The LORs are the mean ± s.e.m. estimates of LOR for these clusters, which represents the change in the log odds of cells per sample from PS19-E3, PS19-E4-R/S or PS19-E4-S/S mice belonging to the respective clusters compared to the log odds of cells per sample from PS19-E4 mice. LOR versus PS19-E4 for subcluster 2: PS19-E4-S/S, 3.02 ± 1.28; subcluster 8: PS19-E4-R/S, −2.58 ± 1.02; PS19-E4-S/S, −3.36 ± 1.17; subcluster 11: 2.69 ± 1.33. **d**, Dot plot of normalized average expression of selected homeostatic and DAM marker genes for microglia subclusters 2, 8 and 11. **e**, Volcano plot for top 30 DE genes of microglia subcluster 8 versus other microglia subclusters. **f**, Volcano plot for top 30 DE genes of microglia subcluster 8 in PS19-E4-S/S versus PS19-E4 mice. **g**, Representative images of Gpnmb^+^Iba1^+^ microglia in the hippocampus of 10-month-old PS19-E4 (*n* = 10) and PS19-E4-S/S (*n* = 10) mice. Scale bars, 50 µm. **h**, Quantification of the number of Gpnmb^+^Iba1^+^ cells (per mm^2^) within the DG of hippocampus. Difference between groups in **h** was determined by unpaired, two-sided Welch’s *t*-test. **i**, Heat map plot of LOR per unit change in each pathological measurement for microglia subclusters 2, 8 and 11. *P* values in **c** are from fits to a GLMM_AM, and *P* values in **i** are from fits to a GLMM_histopathology; the associated tests are two-sided. In **e**,**f**, horizonal dashed line indicates *P* = 0.05, and vertical dashed lines indicate log_2_ fold change = 0.4. The unadjusted *P* values and log_2_ fold change values used were generated from the gene set enrichment analysis using the two-sided Wilcoxon rank-sum test as implemented in the FindMarkers function of the Seurat package. Gene names highlighted in red text indicate that they are selected marker genes for DAMs. All error bars represent s.e.m. AS, astrocyte; FC, fold change; MG, microglia; NS, not significant. Source data

We also subclustered microglia (clusters 17 and 19 in Fig. 6a) and identified 15 microglial subclusters (Fig. 8a). LOR estimates from GLMM_AM revealed that microglia subclusters 2 and 11 were likely to contain more cells from PS19-E4-S/S mice than from PS19-E4 mice, whereas microglia subcluster 8 was likely to contain fewer cells from PS19-E4-S/S mice than from PS19-E4 mice (Fig. 8b,c and Supplementary Table 5). PS19-E4-R/S mice also had a significant decrease in microglia subcluster 8, although to a lesser extent than PS19-E4-S/S mice (Fig. 8b,c and Supplementary Table 5). Microglia subcluster 8 expressed high levels of APOE, whereas microglia subclusters 2 and 11 expressed relatively low levels of APOE (Extended Data Fig. 8e). DE gene analyses comparing each of these microglia subclusters with all other microglia subclusters identified microglia subclusters 2 and 11 as homeostatic microglia and microglia subcluster 8 as disease-associated microglia (DAMs) (Fig. 8d and Supplementary Table 5), with downregulation of homeostatic microglial genes and upregulation of DAM genes, similar to those reported previously^63^. Additionally, DAM subcluster 8 had many highly upregulated DE genes uniquely identified in the current study, including *Igf1*, *Gpnmb*, *Mamdc2*, *Kif13* and *Sash1* (Fig. 8e and Supplementary Table 5), as compared to other microglia subclusters. Strikingly, these highly upregulated DE genes in the DAM subcluster 8 were heavily downregulated in PS19-E4-S/S mice versus PS19-E4 mice (Fig. 8f and Supplementary Table 5). Using Gpnmb as a marker for DAM cluster 8, immunofluorescent staining confirmed a significant decrease in this DAM cluster in the hippocampus of PS19-E4-S/S mice versus PS19-E4 mice (Fig. 8g,h).

Furthermore, DE pathway analysis revealed enrichment of KEGG pathways related to MAPK signaling, cAMP signaling and synaptic function in microglia subclusters 2 and 11 (Extended Data Fig. 8f,h, and Supplementary Table 5), supporting their homeostatic function. Conversely, DAM subcluster 8 had enrichment in KEGG pathways related to lysosome, autophagy and various diseases (Extended Data Fig. 8g and Supplementary Table 5), supporting its disease association. We also observed enrichment of lipid and atherosclerosis pathway genes in DAM subcluster 8 (Extended Data Fig. 8g and Supplementary Table 5). Iba1/BODIPY double staining confirmed neutral lipid accumulation in microglia in PS19-E4 mice but not PS19-E3 mice (Extended Data Fig. 9c,d). Notably, the R136S mutation eliminated neutral lipid accumulation in microglia in PS19-E4-S/S mice (Extended Data Fig. 9c,d), suggesting that the R136S mutation protects against APOE4-induced dysregulation of lipid accumulation in microglia. No significant difference was observed in Lamp1^+^ lysosomal size in microglia with different APOE genotypes (Supplementary Fig. 8c,d).

The LOR estimates from GLMM_histopathology revealed that the proportion of cells in microglia subclusters 2 and 11 exhibited positive associations with hippocampal volume and negative associations with the coverage areas of p-Tau and gliosis, whereas microglia subcluster 8 had a negative association with hippocampal volume and a significant positive association with the coverage areas of p-Tau and gliosis (Fig. 8i and Supplementary Table 6). Together, these findings indicate that the APOE4-R136S mutation increases disease-protective homeostatic microglia (subclusters 2 and 11) and eliminates DAMs (subcluster 8), with E4-S/S having a greater effect than E4-R/S.

Furthermore, PCA analysis revealed that the disease-protective microglia subcluster 2 had a similar contribution to the measured pathologies as the four disease-protective neuronal clusters (1, 6, 7 and 28) (Extended Data Fig. 10). Likewise, DAM subcluster 8 had a similar contribution to the measured pathologies as the DAOs (cluster 9) (Extended Data Fig. 10). These findings again support the notion that APOE4-R136S leads to a significant increase in the disease-protective homeostatic microglia subpopulation, likely contributing to the enrichment of neuronal clusters 1, 6, 7 and 28, as well as to a significant reduction in the DAM subpopulation, likely contributing to the elimination of the DAOs.

## Discussion

The protection conferred by APOE3-R136S against EOAD caused by the PSEN1-E280A mutation is a milestone discovery^36^. It emphasizes the key roles of APOE variants in AD pathogenesis and/or protection. Our approach of studying APOE4-R136S allows us to investigate if and how this mutation may also be protective against AD pathologies promoted by APOE4 (Supplementary Fig. 9). Our data show that homozygous R136S mutation fully protects against APOE4-driven Tau pathology, neurodegeneration and neuroinflammation in a tauopathy mouse model (Supplementary Fig. 9). We also demonstrate the protection of the APOE4-R136S mutation against Tau uptake and p-Tau accumulation in an AD-relevant context using hiPSC-derived neurons (Supplementary Fig. 9).

Previous work showed that HSPGs are largely responsible for neuronal Tau uptake^46–48,57^. Notably, by substituting a positively charged arginine residue for the uncharged serine, the affinity of the receptor-binding domain of APOE-R136S for negatively charged HSPGs is greatly diminished^36,45,58^. We used isogenic hiPSC-derived neurons to study the effect of the R136S mutation on Tau uptake by human neurons. Our findings demonstrate that the R136S mutation reduces neuronal p-Tau accumulation partially by reducing APOE4-promoted Tau uptake due to the defective HSPG binding of APOE4-R136S. Thus, development of therapeutics targeting HSPG binding of APOE4 could be a viable approach to reduce Tau pathology and its resulting neurodegeneration and neuroinflammation.

The interactive effects of Tau pathology and gliosis on neurodegeneration were studied previously^64^, and APOE4 exacerbates these phenotypes in a PS19 tauopathy model^7,11,55^. We reveal an intriguing uncoupling of APOE4-driven Tau pathology and gliosis as drivers of neurodegeneration in PS19-E4-R/S mice. Notably, the heterozygous R136S mutation only partially protects against some of the detrimental effects of APOE4 (Supplementary Fig. 9). Although PS19-E4-R/S mice and E4-R/S neurons did not show protection from APOE4-induced p-Tau accumulation, the R136S mutation did enable gene dose-dependent rescue of APOE4-promoted astrocytosis, microgliosis and neurodegeneration. Heavier astrocytosis correlates with hippocampal atrophy in PS19-E4 mice, but this relationship was mostly abolished in PS19-E4-S/S mice and somewhat diminished in PS19-E4-R/S mice. Interestingly, both heavier (PS19-E4 mice) and mild (PS19-E3, PS19-E4-S/S and PS19-E4-R/S mice) microgliosis correlate with hippocampal atrophy, suggesting that microgliosis may contribute to degeneration at an earlier stage of pathogenesis. In PS19-E4-R/S mice, we saw high p-Tau burden (similar to PS19-E4 mice) yet partially rescued astrocytosis and microgliosis, suggesting that neurodegeneration-associated gliosis is not solely induced by Tau pathology. This highlights the related but distinct roles of APOE variants in different AD pathologies, which likely depend on cell type. It is, thus, plausible to speculate that the remaining astrocytosis and microgliosis in PS19-E4-R/S mice (compared to PS19-E4-S/S mice), together with the Tau pathology, likely contributes to the remaining hippocampal atrophy.

Overall, these data suggest (1) that a single copy of the R136S mutation is not sufficient to overcome the detrimental effects of APOE4 on neuronal Tau pathology, as also shown in the original case report for heterozygous APOE3-R136S carriers with PSEN1-E280A mutation^36^ and (2) that glial cells may be more sensitive to the presence of APOE4-R136S and be more protected against APOE4 effects than neurons. Thus, healthier populations of astrocytes and microglia may mitigate the strong contribution of Tau pathology to neurodegeneration in the PS19-E4-R/S mice.

To further examine the protective effects of APOE4-R136S mutation in a cell-type-specific manner, we conducted snRNA-seq analysis. There is a gene dose-dependent effect of the R136S mutation on increasing disease-protective neuronal, astrocytic and microglial subpopulations and decreasing disease-associated subpopulations of oligodendrocytes (DAOs), astrocytes (DAAs) and microglia (DAMs). The DAAs and DAMs have transcriptomic profiles similar to those reported previously in brains of patients with AD and mouse models^62,63^. These disease-associated subpopulations were enriched in APOE4-expressing tauopathy mice and correlated to the severity of Tau pathology, gliosis and neurodegeneration. Deeper analyses of DE genes revealed that these DAOs, DAAs and DAMs each have unique transcriptomic signatures that are eliminated or even reversed with the homozygous APOE4-R136S mutation. These data indicate that the R136S mutation not only modifies APOE4-driven AD pathologies in general, but it does so by differentially affecting the disease-associated transcriptomes in specific types of cells. These findings warrant future studies, and further analysis and validation of these cell-type-specific effects may provide clues to the underlying mechanisms of the AD-protective APOE-R136S mutation.

This study also has some limitations. The original case report detailed protection of the APOE3-R136S mutation against autosomal-dominant AD, particularly in the face of extremely high Aβ burden^36^. In the present study, we demonstrate the protective power of the R136S mutation against APOE4-driven effects in a tauopathy mouse model and in hiPSC-derived neurons from a patient with AD, neither of which exhibit extensive Aβ pathology. Thus, our study does not address the question as to whether the APOE-R136S mutation also protects against Aβ-mediated neuroinflammatory and neurodegenerative effects^65–69^. The field would benefit from such future studies. Furthermore, we strictly aimed to study if the R136S mutation overcomes the detrimental effects of APOE4, especially as a model for LOAD. The original patient showed AD protection with the homozygous APOE3-R136S mutation^36^. It will be important for future studies to investigate whether the R136S mutation also provides protection against AD pathologies in the context of APOE3.

Overall, this study illustrates the AD-protective effects of the R136S mutation against APOE4-promoted pathologies in a gene dose-dependent manner, both in vivo in a tauopathy mouse model and in vitro in hiPSC-derived neurons (Supplementary Fig. 9). This study also provides potential molecular and cellular mechanisms by which the APOE-R136S mutation confers protection as potential targets for therapeutic development against APOE4-related AD and tauopathies.

## Methods

### Generation of APOE4-R136S knock-in mice by CRISPR–Cas-9-mediated gene editing

All animal experiments were conducted in accordance with the guidelines and regulations of the National Institutes of Health, the Institutional Animal Care and Use Committee at the University of California, San Francisco (UCSF) and the Gladstone Institutes under protocol AN176773.

Human LoxP-floxed APOE-KI (E-KI) mice were previously generated, in which the entire mouse *Apoe* gene, except exon 1, was replaced by human *APOE3* or *APOE4* gene, respectively^52^. To generate F0 founders of E4-KI mice harboring the R136S mutation (E4-S/S-KI), a CRISPR–Cas9-based knock-in strategy was employed. The guide RNA (gRNA) and single-stranded oligodeoxynucleotide (ssODN) template were paired and injected into fertilized zygotes to introduce the point mutation of R136S into APOE4 (ref. ^70^). gRNAs were designed with multiple online tools, including CCTop^71^, CRISPOR^72^ and Integrated DNA Technologies (IDT) Custom Alt-R CRISPR–Cas9 gRNA. To increase editing efficient, gRNAs were selected based on the proximity of their cutting site to the expected mutation, high targeting efficiency and low predicted off-target effects.

To generate sgRNA, crRNA (GCTTGCGCAGGTGGGAGGCG) was combined with tracrRNA in a 1:1 ratio (IDT), heated to 95 °C for 5 min and allowed to slowly cool to room temperature. Cas9 protein (IDT) was added to form the Cas9 ribonucleoprotein (Cas9 RNP) complex. The ssODN donor repair template was synthesized by IDT (G*G*AGGACGTGCGCGGCCGCCTGGTGCAGTACCGCGGCGAGGTGCAGGCCATGCTCGGCCAGAGCACCGAGGAGCTGCGGGTGAGCTTAGCCTCCCACCTGCGCAAGCTGCGTAAGCGGCTCCTCCGCGATGCCGATGACCTGCAGAAGCGCCTGGCAGTGTACCAGGCCGGGGCCCGC*G*A) and added later to prepare for injection. The ssODN was designed to convert APOE4 to APOE4-R136S with a silent mutation at the protospacer adjacent motif (PAM) site to prevent repeated Cas9 editing. Two phosphorothioate modifications were required to protect the ends.

To prepare fertilized zygotes, super-ovulated female C57BL/6 mice (4 weeks old) were mated to E4-KI males. Fertilized zygotes (1-cell stage) were collected from oviducts and injected with Cas9 protein (20 ng µl^−1^), crRNA and tracrRNA (15 ng µl^−1^) and ssODN template (10 ng µl^−1^) into pronucleus of the fertilized zygotes. The injected zygotes were implanted into oviducts of pseudopregnant CD1 female mice. Positive F0 founders were identified by gDNA screening. First, for F0 pups, 3–5-mm tail tips were cut and digested by proteinase K for gDNA extraction at the age of 4 weeks. A 530-base pair (bp) genomic region of the *APOE* exon 4 covering the targeted mutations, including 112R and 136R/S, was amplified by polymerase chain reaction (PCR) using primers (FW 5′-*TAAGCTT*GGCACGGCTGTCCAAGGA-3′ and REV 5′-GCTGCATGTCTTCCACCAG-3′) with high-fidelity KOD Plus DNA polymerase (F0934K, TOYOBO). This 530-bp PCR product was used as the template of real-time PCR screening with primers designed to include the expected mutation at the 3′ end: FW 5′-GGAGCTGCGGGTGAGCTTA-3′ and REV 5′-CTGGTACACTGCCAGGCG-3′. With these mutation-specific primers, potential positive F0 candidates showed a much smaller ΔCt (<2) when compared to the Ct value of internal control primers. Lastly, the 530-bp PCR product was used to verify the existence of the expected mutation by Sanger DNA sequencing. The *APOE* gene sequence in F1 breeders was verified to be unaltered except for the expected R136S mutation. CRISPOR was used to predict all potential off-target sites in the genome^72^, and those sites located in exon regions on chromosome 7 were verified to be intact by Sanger sequencing.

### Generation of tauopathy mice expressing E4, E3 or E4-S/S

The E3, E4 and E4-S/S mice were cross-bred with PS19 mice expressing human P301S 1N4R Tau driven by the PrP promoter (B6;C3-Tg(Prnp-MAPT*P301S)PS19Vle/J, from The Jackson Laboratory) to generate PS19-E4, PS19-E3, PS19-E4-S/S and PS19-E4-R/S mice. All mice were on a pure C57BL/6 genetic background and were housed in a pathogen-free barrier facility on a 12-h light cycle at 19–23 °C and 30–70% humidity. WT mice on a C57BL/6 background (000664, The Jackson Laboratory) were bred separately from the PS19-E mice and used for control studies. Animals were identified by ear punch under brief isoflurane anesthesia and genotyped by PCR of a tail clipping as described above. All animals otherwise received no procedures. For all studies, both male and female mice were used.

### Collection of mouse tissue

Ten-month-old or 6-month-old mice were deeply anesthetized with intraperitoneal injections of avertin (Henry Schein) and transcardially perfused for 1 min with 0.9% saline. Right hemi-brains were drop-fixed in 4% paraformaldehyde for 48 h (15710, Electron Microscopy Sciences), rinsed in PBS (Corning) for 24 h and cryoprotected in 30% sucrose (Sigma-Aldrich) for 48 h at 4 °C. The fixed right hemi-brains were sliced into 30-µm coronal sections spanning the hippocampus on a freeze sliding microtome (Leica) and stored in cryoprotectant (30% ethylene glycol, 30% glycerol, 40% 1× PBS) at −20 °C. Left hemi-brains were snap frozen on dry ice and stored at −80 °C for further analyses.

### hiPSC culture

The hiPSC protocol was approved by the Committee on Human Research at UCSF. The E4/4 hiPSC line was generated as described^73,74^ from skin fibroblasts of a subject with an APOE4/4 (E4) genotype. The isogenic E3/3 (E3) hiPSC line was generated from this parental E4/4 hiPSC line as previously described^6^. The EKO hiPSC line was generated as previously described^6^ from a subject with APOE deficiency. hiPSCs were maintained in mTeSR medium (85850, STEMCELL Technologies) on six-well plates pre-coated with hESQ, LDEV-free Matrigel (354277, Corning). The medium was changed daily, and cells were routinely passaged 1:10–1:15 using Accutase (NC9464543, Thermo Fisher Scientific) for dissociation. Rho kinase (ROCK) inhibitor (1254, Tocris) was added to medium at 10 µM on the day of passaging.

### Generating and characterizing isogenic E4-S/S and E4-R/S hiPSC lines by CRISPR–Cas-9-mediated gene editing

To generate isogenic E4-S/S and E4-R/S hiPSC lines, CRISPR–Cas9 editing strategy was used^75^. sgRNAs were designed using CRISPOR to optimize for distance of cut site to mutation, high targeting efficiency and minimal predicted off-target sites^70,76^. The top sgRNA was selected after testing cutting efficiency in pooled, nucleofected cells using the Synthego ICE analysis tool (Synthego Performance Analysis, 2019). To further increase on-target efficiency and minimize off-target editing, S.p. HiFi Cas9 Nuclease was used^77^.

Once the top sgRNA was selected, the parental E4 hiPSCs were dissociated in Accutase, and 8 × 10^5^ hiPSCs were pelleted before resuspension in nucleofection solution (Lonza) per the Amaxa Human Stem Cell Nucleofector Kit 1 (VPH-5012). For Cas9 RNP preparation, 300 pmol of sgRNA (CTTACGCAGCTTGCGCAGGT) (Synthego) targeting exon 4 of the *APOE* gene around Arg136 of the APOE4-coding allele and 40 pmol of S.p. HiFi Cas9 Nuclease V3 (IDT) were mixed for 10 min at room temperature. Then, 4 µl of Cas9-RNP and 1 µl of ssODN donor repair template synthesized by IDT (GGCCTGGTACACTGCCAGGCGCTTCTGCAGGTCATCGGCATCGCGGAGGAGCCGCTTACGCAGCTTGCGCAGGTGTGAGGCGAGGCTCACCCGCAGCTCCTCGGTGCTCTGGCCGAGCATGGCCTGCACCTCGCCGCGGTACTGCACCAG) were added to 100 µl of cells in nucleofection solution. The ssODN was designed to convert APOE4 to APOE4-R136S with a silent mutation at the PAM site to prevent repeated Cas9 cutting in edited cells. The mixture was transferred to a cuvette and nucleofected with Nucleofector II (Lonza) at Nucleofector setting A-030. Next, 500 μl of pre-warmed (37 °C) mTeSR medium with 10 µM ROCK inhibitor was immediately added into the nucleocuvette to recover nucleofected cells. The cell mixture was then pipetted into a single well of an hESQ-Matrigel pre-coated six-well plate with mTeSR and 10 µM ROCK inhibitor to recover. After 3–5 d, cells were dissociated in Accutase to a single-cell solution and plated to obtain individual colonies using both limiting dilution and colony picking methods in mTeSR and CloneR (05888, STEMCELL Technologies) for viability. For limiting dilution, 0.5 cells per well were seeded onto hESQ-Matrigel pre-coated 48-well plates. For colony picking, 1 × 10^3^ cells were sparsely plated into an hESQ-Matrigel pre-coated 10-cm dish. After 3–5 d, surviving colonies from both methods were picked, transferred to hESQ-Matrigel pre-coated 48-well plates and expanded to isolate gDNA for screening. A genomic fragment spanning the sgRNA target sites on exon 4 of the *APOE* gene was amplified using primers (FW 5′-TAAGCTTGGCACGGCTGTCCAAGGA-3′ and REV 5′-GCTGCATGTCTTCCACCAG-3′) with Qiagen Taq DNA Polymerase using a Quick Start Protocol for GC-rich region (201443, Qiagen). The amplified genomic DNA fragment was Sanger sequenced. The top predicted off-target cut sites in coding regions by CHOPCHOP^78^ and COSMID^79^ were verified to be intact by Sanger sequencing. Each unique hiPSC line was checked for normal karyotype and validated to maintain pluripotency (see ‘Immunocytochemistry and imaging’ subsection for details). In total, three hiPSC lines homozygous for R136S mutation and two hiPSC lines heterozygous for R136S mutation were generated and characterized for use in the current study.

### Neuronal differentiation of hiPSCs

hiPSCs were differentiated into neurons as previously described^6,56^, with slight modifications to increase yield. In brief, hiPSCs were dissociated with Accutase for 5–8 min before being quenched with warm (37 °C) N2B27 medium made of 1:1 DMEM/F12 (11330032, Thermo Fisher Scientific) and Neurobasal Media (21103049, Thermo Fisher Scientific), 1% N2 Supplement (21103049, Thermo Fisher Scientific), 1% B27 (17504044, Thermo Fisher Scientific), 1% MEM non-essential amino acids (11140050, Thermo Fisher Scientific), 1% GlutaMAX (35050061, Thermo Fisher Scientific) and 0.5% penicillin–streptomycin (15140122, Thermo Fisher Scientific). Dissociated hiPSCs were pelleted by centrifugation, resuspended in embryoid body media (10 µM SB431542 (1614, Tocris) and 0.25 µM LDN (04-0074, Stemgent) in N2B27) with 10 µM ROCK inhibitor (1254, Tocris) and grown in suspension in a T-75 flask (12-565-349, Thermo Fisher Scientific). Flasks were shaken manually once per hour for the first 3 h of incubation. On day 2, embryoid bodies had formed, and fresh embryoid body medium was replaced (embryoid bodies pelleted, old medium aspirated, cells resuspended in fresh medium) to remove ROCK inhibitor. Embryoid body medium was replaced similarly on days 4 and 6. On day 8, spheres were plated as neural progenitors onto a 10-cm dish pre-coated with growth factor reduced (GFR) Matrigel (CB-40230A, Thermo Fisher Scientific). Neural progenitors were allowed to form neuronal rosettes and sustained in N2B27 media alone for days 8–15. Half of the media was replaced every 48–72 h depending on confluency and media consumption. Neural rosettes were lifted on day 16 using STEMdiff Neural Rosette Selection Reagent (05832, STEMCELL Technologies) as directed by the manufacturer and plated into three wells of a six-well plate pre-coated with GFR Matrigel in N2B27 with 100 ng ml^−1^ FGFb (100-18B, PeproTech) and 100 ng ml^−1^ EGF (AF-100-15, PeproTech). This N2B27 medium with FGFb and EGF was replaced daily. On day 20, neural progenitors were dissociated with Accutase, quenched with N2B27 and resuspended in STEMdiff Neural Progenitor Medium (05833, STEMCELL Technologies) at 1.2 × 10^6^ cells per 2 ml for one well of a six-well plate, pre-coated with GFR Matrigel. Neural progenitor cells were fed with fresh Neural Progenitor Medium daily. On day 28, cells were plated as neurons. Neural progenitor cells were dissociated with Accutase; N2B27 media were added to bring volume of cell suspension to 40 ml; and cells were filtered through a 40-µm cell strainer (08-771-1, Thermo Fisher Scientific) to ensure single-cell suspension. Cells were collected by centrifugation, resuspended in complete neuronal medium (10 ng ml^−1^ BDNF (450-02, PeproTech) and 10 ng ml^−1^ GDNF (450-10, PeproTech) in N2B27) with 10 nM DAPT (2634, Tocris), counted and plated at a concentration of 2 × 10^5^ cells per well onto 12-mm coated glass coverslips (354087, Corning) in a 24-well plate. For cells used for immunohistochemical staining, coverslips coated with poly-l-lysine (P4707, Sigma-Aldrich) and mouse laminin (23017015, Gibco) were put into each well before cell plating. Seventy-five percent of culture medium was replaced on maturing neurons every 3–4 d. DAPT was removed after the first week. Most experiments were performed on neuronal cultures that had been differentiated 3–4 weeks. Tau uptake assay experiments were performed on neurons aged 5–6 weeks.

### Immunohistochemistry

Several sections from each mouse (30 µm thick, 300 µm apart) were transferred to a 24-well plate and washed 3 × 5 min with PBS-T (PBS + 0.1% Tween 20) to remove cryoprotectant. For diaminobenzidine (DAB) staining, citrate buffer (0.1 M sodium citrate, 0.1 M citric acid in PBS, microwaved until boiling) was applied to brain sections for 5 min for antigen retrieval. Sections were washed 2 × 5 min in PBS-T, incubated in endogenous peroxidase block (0.3% H_2_O_2_ (Sigma-Aldrich) and 10% methanol (Thermo Fisher Scientific) in PBS) for 15 min, washed once in PBS-T for 5 min and blocked for non-specific binding in a solution of 10% normal donkey serum (017000121, Jackson ImmunoResearch) and 1% non-fat dry milk in PBS-T for 1 h at room temperature. After blocking, sections were washed 2 × 5 min in PBS-T before undergoing an additional avidin/biotin block (SP-2001, Vector Labs) as directed for 15 min and then washed 2 × 5 min in PBS-T. Sections were incubated in M.O.M. blocking buffer (one drop M.O.M. IgG per 4 ml of PBS-T) (MKB-2213-1, Vector Labs) for 1 h at room temperature, washed 2 × 5 min in PBS-T and incubated in primary antibody (AT8 (MN1020, Thermo Fisher Scientific, 1:100)) overnight at 4 °C in a solution of 5% normal donkey serum and PBS-T. The next day, sections were washed 3 × 5 min with PBS-T and incubated in biotinylated secondary antibody (715-065-150, Jackson ImmunoResearch, 1:200) for 1 h at room temperature. Sections were then washed 2 × 5 min in PBS-T before incubation in avidin–biotin complex (PK-6100, Vector Labs) as directed. Sections were washed 2 × 5 min in PBS-T and once in Tris buffer (pH 8.0) for 5 min. Sections were incubated in DAB mixture (SK-4100, Vector Labs) per the manufacturer’s instructions for exactly 1.5 min before immediate solution removal and washing with Milli-Q followed by 2 × 5-min PBS-T washes. Sections were mounted and dried overnight at room temperature. The next day, sections were submerged 2 ×5 min in xylene (Thermo Fisher Scientific) and coverslipped with DPX mounting medium (06522, Sigma-Aldrich). Slides were imaged with an Aperio VERSA slide scanning microscope (Leica) at ×10 magnification.

For immunofluorescent staining, antigen retrieval was accomplished with Tris buffer, pH 8.0 (46-031-CM, Corning; microwaved until boiling) applied to brain sections for 5 min. Next, sections were washed 2 × 5 min in PBS-T and blocked for non-specific binding in a solution of 5% normal donkey serum (017000121, Jackson ImmunoResearch) and 0.2% Triton-X (Millipore Sigma) in PBS for 1 h at room temperature. After blocking, sections were washed 2 × 5 min in PBS-T before incubating in M.O.M. blocking buffer (one drop M.O.M. IgG per 4 ml of PBS-T) (MKB-2213-1, Vector Labs) for 1 h at room temperature, washed 2 × 5 min in PBS-T and stained overnight at 4 °C in a solution of 5% normal donkey serum and PBS-T with the following primary antibodies: APOE (13366, STEMCELL Technologies, 1:500); CD68 (MCA1957, Bio-Rad, 1:200); GFAP (MAB3402, Sigma-Aldrich, 1:800; Z0334, Agilent, 1:500; 13-0300, Thermo Fisher Scientific, 1:800); Iba1 (019-19741, Wako, 1:200; ab5076, Abcam, 1:100); Gpmnb (56898S, Cell Signaling Technology, 1:600); Id3 (PA5-100268, Thermo Fisher Scientific, 1:200); Kirrel3 (PA5-63287, Thermo Fisher Scientific, 1:500); LAMP1 (14-1071-82, Thermo Fisher Scientific, 1:500); Nkain2 (ABGEAP16664C, VWR, 1:500); Olig2 (AF2418, R&D Systems, 1:800); and S100β (ab52642, Abcam, 1:200). The next day, sections were washed 3 × 5 min with PBS-T and incubated in relevant fluorescently labeled secondary antibodies—Alexa Fluor (Jackson ImmunoResearch, 1:1,000) in PBS-T and DAPI (Thermo Fisher Scientific, 1:20,000)—for 1 h at room temperature. Sections were washed 2 × 5 min in PBS-T and mounted onto microscopy slides, coverslipped with ProLong Gold mounting medium (P36930, Vector Labs) and sealed with clear nail polish. Slides were imaged with an Aperio VERSA slide scanning microscope (Leica) at ×10 magnification or a FV3000 confocal laser scanning microscope (Olympus) at ×20, ×40 or ×60.

For immunofluorescent studies co-stained with BODIPY, a similar protocol was followed as described above with the following modifications: no antigen retrieval step, and all washes were performed with PBS (but antibody incubations still contained PBS-T). Tissue was incubated with fluorescently labeled secondary antibodies along with 1 mg ml^−1^ BODIPY (D3922, Thermo Fisher Scientific, 1:1,000) for 45 min. Slides were images with confocal (Olympus) microscope at ×20 with ×3 digital zoom. Analysis of neutral lipid puncta number was performed as described^80^. In brief, GFAP^+^ or Iba1^+^ cells from the DG area that co-localized with BODIPY^+^ neutral lipids were manually counted from maximum projection of *z*-stack across the whole section. The counts were normalized to the corresponding GFAP^+^ or Iba1^+^ cells to get the fraction of GFAP^+^BODIPY^+^/GFAP^+^ cells or Iba^+^BODIPY^+^/Iba1^+^ cells. Analysts were blinded to sample.

For all analyses measuring immunostaining as percent coverage area of a region of interest or quantifying cell number per area (mm^2^), two sections per mouse were stained, imaged and quantified, and the measurements were averaged. To quantify, the hippocampal regions were manually drawn in Fiji (ImageJ)^81^ and set to a standard threshold value predetermined for each stain. For analyses measuring cell number per area, cell counts were then normalized to area of manually drawn hippocampus. Analyses were conducted via automated ImageJ macros to the extent possible to remove human bias. Analysts drawing regions of interest and setting standard threshold values were blinded to sample also, to exclude the possibility of bias. Categorization of the p-Tau staining type was performed manually, and analysts were blinded to sample.

To quantify the number of AT8^+^ soma within the CA1 subregion of the hippocampus as visualized with DAB, a mask of the CA1 cell layer was first manually drawn and area (mm^2^) measured for each image in Fiji (ImageJ). The AT8-DAB image and corresponding mask were then further analyzed with CellProfiler version 4.2.1 (ref. ^82^). Segmentation analysis of cell soma positive for AT8 was also performed using first ColorToGray and ImageMath to invert the image, followed by image processing modules Smooth and EnhanceOrSuppressFeatures to better discern AT8 positivity in cell soma. IdentifyPrimaryObjects was then used to count AT8^+^ cell soma. These objects were then counted only if they fell within the CA1 masked region. The number of AT8^+^ cell somas were then normalized to the area in mm^2^. During both the manual drawing of the hippocampal subregion of the CA1 cell layer in Fiji (ImageJ) and the AT8^+^ cell soma quantification in CellProfiler, analysts were blinded to sample identity.

Analyses for disease-associated gene immunostaining validation were performed using CellProfiler version 4.2.1 (ref. ^82^). Images were collected on an FV3000 confocal laser scanning microscope (Olympus); each channel was separated into slice files using ImageJ script; and segmentation analysis of glial cell type was performed using IdentifyPrimaryObjects on GFAP, Iba1 or Olig2 stained slice. For immunostaining Id3-GFAP, Kirrel3-Olig2 and LAMP1-GFAP-Iba1, cells were segmented from slices that underwent maximum projection of *z*-stack across the whole section in Fiji (ImageJ) before import into CellProfiler. Segmentation analysis of cells positive for Nkain2, Id3, Gpnmb or Kirrel3 was also performed using IdentifyPrimaryObjects. Image processing modules ReduceNoise and EnhanceOrSuppressFeatures were used when needed. To count glial cells positive for protein of interest, objects identified by glial stains and the objects of the corresponding disease-associated protein were then counted using RelateObjects. This was an automated process applied to all images of a given stain. The number of cells positive for both glial marker and corresponding protein of interest were then normalized to the area in mm^2^. For Gpnmb/Iba1 immunostaining study, NeuN was stained and masked out of analysis to account for any Gpnmb that was expressed within the neuronal layer of the DG.

Analyses for LAMP1^+^ glial cell stains were also performed using CellProfiler. Images collected on the Olympus microscope were processed as describe above. Measure of area of each LAMP1^+^ object also positive for GFAP or Iba1 was also calculated to obtain size in pixels.

### Western blot analysis

Left hemi-brains of mice previously snap frozen on dry ice and stored at −80 °C were then thawed on ice for homogenization. Hippocampal tissue was weighed and homogenized using a Polytron immersion disperser homogenizer (Kinematica) in high-detergent buffer at 10 µl mg^−1^ tissue: 50 mM Tris, 150 mM NaCl, 2% NP-40 (Sigma-Aldrich), 1% sodium deoxycholate and 4% sodium dodecyl sulfate and supplemented with complete protease inhibitor cocktail pellet (Roche, 11836145001), phosphatase inhibitor cocktail 2 (P5726, Sigma-Aldrich), phosphatase inhibitory cocktail 3 (P0044, Sigma-Aldrich) and Benzonase Nuclease (E1014, Sigma-Aldrich). Samples were then centrifuged at maximum speed for 5 min. The supernatant was collected and stored at −80 °C before analysis by western blot.

Cultured hiPSC-derived neurons were washed with PBS and harvested for western blot analysis as previously described^6^. In brief, neurons were collected in the presence of a high-detergent buffer as described above and supplemented with complete protease inhibitor cocktail pellet, phosphatase inhibitor cocktail 2, phosphatase inhibitory cocktail 3 and Benzonase Nuclease.

For analysis by western blot, samples were separated by SDS–PAGE on 12% NuPAGE Bis-Tris polyacrylamide gels (Thermo Fisher Scientific) using MOPS buffer and transferred to nitrocellulose membranes at 18 V for 60 min (Trans-Blot Turbo Transfer System, Bio-Rad). The membranes were then blocked in Intercept Blocking Buffer (PBS) (927-70001, LI-COR) for 1 h at room temperature and probed with primary antibodies overnight at 4 °C to the following proteins: APOE (178479, Sigma-Aldrich, 1:5,000), p-Tau (AT8 (MN1020, Thermo Fisher Scientific, 1:2,000)) or p-Tau (PHF1 (gift from Peter Davies, 1:2,000)). The secondary antibodies were IgG labeled with IRDye 800 or IRDye 680 (LI-COR), including donkey anti-rabbit 680 (926-68073, 1:20,000); donkey anti-mouse 800 (926-32212, 1:20,000); and donkey anti-goat 800 (926-32214, 1:20,000). The blotted membranes were scanned with an Odyssey CLx Imaging System (LI-COR). Signals were measured as fluorescence intensity of bands with Image Studio Lite 5.2.5 (LI-COR), which preserves raw data values independent of adjustments in image brightness display. Samples were quantified as ratio of target protein to neuronal protein control: APOE:TUJ1, AT8:TUJ1 and PHF1:TUJ1. In some cases, blots were stripped (928-40028, LI-COR) and re-probed for antibody of interest and TUJ1 as control.

### Immunocytochemistry and imaging

Cells were plated on 12-mm coated glass coverslips in a 24-well plate. Coverslips were coated with hESQ-Matrigel (354277, Corning) for hiPSC culture staining or poly-l-lysine and mouse laminin as described for neuron culture staining before plating. Cells were fixed in 4% paraformaldehyde for 15 min, washed 3 × 5 min with 1× DPBS (14080055, Gibco) and permeabilized with 0.5% Triton-X for 5 min, followed by 1-h blocking at room temperature with 10% normal donkey serum (017000121, Jackson ImmunoResearch) and 0.5% Triton-X in PBS. After blocking, cells were stained with primary antibodies overnight at 4 °C to the following proteins: APOE (13366, STEMCELL Technologies, 1:800); GFAP (MAB3402, Sigma-Aldrich, 1:5,000); GABA (A2052, Sigma-Aldrich, 1:1,000); human nuclei (ab84680, Abcam, 1:200), MAP2 (AB5622, EMD Millipore, 1:1,000; PA1-10005, Thermo Fisher Scientific, 1:5,000), Olig2 (AF2418, R&D Systems, 1:1,000); NANOG (09-0020, Stemgent, 1:100), OCT3/4 (sc-5279, SCBT, 1:100), p-Tau (PHF1 (gift from Peter Davies, 1:300)), SOX2 (sc-365823, SCBT, 1:200), total Tau (ab62639, Abcam, 1:800) and TRA-1-60 (MAB4360, EMD Millipore, 1:100). The secondary antibodies were IgG conjugated to Alexa Fluor 488 or Alexa Fluor 594 (against rabbit, mouse or goat IgG (cat. nos. A-21206, A-21207, A-21202, A-21203, A-11058 and A-11055, from Invitrogen, 1:1,500)) and Alexa Fluor 647 against rabbit (ab150075, Abcam 1:1,500). Coverslips were mounted to microscope slides with VECTASHIELD Prolong Gold with DAPI (H-1200-10, Vector Labs). Images were taken with an FV3000 confocal laser scanning microscope (Olympus) at ×20 or ×60. Image analysis to quantify positivity in hiPSC-derived neuron stains was performed using custom macros written in the open-source Fiji (ImageJ) software. For the channels of PHF1 and MAP2 positivity, a standard threshold value was chosen and automatically applied to each channel of each image, and total area of immunoreactivity was measured. Quantified values reflect area of PHF1:MAP2 ratio. To obtain the PHF1^+^ puncta area, the PHF1 channel was additionally processed using Analyze Particles to count PHF1^+^ regions of a predetermined size range. This was then normalized to MAP2^+^ area. To count the number of GABA^+^ inhibitory neurons in the culture, standard thresholds were chosen and automatically applied to each GABA, DAPI and MAP2 channels. To count total neurons, DAPI^+^MAP2^+^ cells were counted using Analyze Particles. To count GABA^+^ neurons, GABA^+^DAPI^+^MAP2^+^ neurons were counted using Analyze Particles.

### Labeling Tau

Recombinant 2N4R tau was purchased in lyophilized form (AS-55556, AnaSpec). The protein was dissolved into 0.1 M sodium bicarbonate, pH 8.3, and labeled with Alexa Fluor 488 5-SDP Ester (A30052, Invitrogen), mixing for 1 h at room temperature following the manufacturer’s protocol. After labeling, the proteins were subjected to Zeba Dye and Biotin Removal Spin Columns (A44298, Thermo Fisher Scientific) to remove unreacted dye. Degree of labeling (~3.5 mole dye per mole of protein) was determined by obtaining protein concentration by absorbance at 280 nm and at 494 nm (maximum absorption wavelength for dye) via NanoDrop per Molecular Probes/Invitrogen instructions. Labeled Tau protein was aliquoted and stored at −20 °C until use.

### Tau uptake assay

Neuronal medium was fully removed; wells were washed with PBS; and fresh complete neuronal medium containing 25 nM Tau-488 was added. For heparin competition studies, 100 µg ml^−1^ heparin (H3149, Sigma-Aldrich) was also added into the medium. Neurons were treated for 1 h at 37 °C. Then, neurons were washed two times with PBS to wash out unbound Tau-488. Accutase was used to dissociate and lift neurons into single cells, and the reaction was quenched with N2B27 medium. Neurons were pelleted by centrifugation, washed with PBS, counted and resuspended in FACS buffer (1% heat-inactivated FBS, DNAse I (04536282001, Roche, 1:1,000)) to 1 × 10^6^ cells per milliliter and filtered through 35-µm mesh, and DAPI was used to exclude dead cells from analysis. Cells were analyzed using BD LSRFortessa X-20 (BD Biosciences). Each experiment was conducted four times and normalized to E4 neurons without heparin treatment to combine datasets from multiple experiments. Roughly 1 × 10^5^ to 5 × 10^5^ events were counted in each experiment. The gating strategy is described in Extended Data Fig. 4. Control samples of neurons untreated with Tau-488 were included in each experiment to accurately draw Tau-488^−^ gates in the live cell population (Extended Data Fig. 4) and automatically propagated to all samples in each experiment. Identical experiments to control for Tau-488 binding versus uptake were conducted at 4 °C (Extended Data Fig. 4). To control for other potential cellular processes contributing to 488 positivity or autofluorescence in response to recombinant Tau, E4 cells were treated with 25 nM unlabeled recombinant Tau for 1 h at 37 °C as described before analysis by flow cytometry (Extended Data Fig. 4). Flow cytometry data were analyzed using FlowJo version 10.8.0 (BD Biosciences). Analysis was performed on a population of roughly 5,000 live cells to normalize cell number for comparison of the distribution of subpopulations (Tau-488^−^ and Tau-488^+^) across samples.

### Long-term treatment of hiPSC-derived neurons with heparin

On day 1, neuronal medium was removed from wells and replaced with fresh complete neuronal medium supplemented with 100 µg ml^−1^ heparin (H3149, Sigma-Aldrich). On days 2 and 3, heparin was added to wells without replacing medium to reach 100 µg ml^−1^. On day 4, neuronal lysate was collected for western blot analysis. Control wells underwent equivalent media changes and handling.

### E4-conditioned medium study

E4-conditioned medium for neuronal culture treatment consisted of equal parts fresh complete neuronal medium as described above, and neuronal medium was collected and filtered (0.2 µm) from 3–4-week-old E4 neurons. Media from 3-week-old E4, E3, E4-S/S and E4-R/S neurons were fully removed, and conditioned medium was applied to cultures. Neurons were fed every 2–3 d with E4-conditioned medium for 2 weeks before lysis and analysis by western blot.

### Volumetric analysis

Every tenth coronal brain section spanning the hippocampus was mounted on a microscope slide for each mouse (seven sections per mouse, 30 µm thick, 300 µm apart) and dried for 1 h at room temperature. All mounted sections were stained with Sudan black as previously reported^7^. Briefly, mounted sections were stained with 1% Sudan black in 70% ethanol at room temperature for 10 min, washed three times in 70% ethanol for 5 min, washed three times with Milli-Q for 5 min and coverslipped with ProLong Gold mounting medium. The stained slices were imaged with an Aperio VERSA slide scanning microscope (Leica) at ×10 magnification. For hippocampal and lateral ventricle volumetric analyses, regions of interest were drawn Fiji (ImageJ), area was measured and volume was calculated using the formula: volume = (sum of area of all seven sections) × 0.3 mm. Quantification encompassed sections roughly between the coordinates AP = −1.2 mm and AP = −3.4 mm. Analysts drawing regions of interest were blinded to sample to exclude the possibility of bias.

### DG GC layer thickness measurement

Two brain sections per mouse (30 µm thick, 300 µm apart) were stained with NeuN (ABN90, Sigma-Aldrich, 1:500) and imaged with an Aperio VERSA slide scanning microscope (Leica) at ×10 magnification as described above. The DG GC layer thickness was measured manually^7,22^. A line was drawn perpendicular to the NeuN^+^ cell layer at six locations in two slices in Fiji (ImageJ), and the average was taken for each mouse. Analysts measuring cell layer thickness were blinded to sample to exclude the possibility of bias.

### Single-nuclei preparation for 10x loading

The mouse hippocampus was dissected on ice and placed into a pre-chilled 2-ml Dounce with 1 ml of cold 1× Homogenization Buffer (1× HB) (250 mM sucrose, 25 mM KCL, 5 mM MgCl_2_, 20 mM Tricine-KOH, pH 7.8, 1 mM DTT, 0.5 mM sermidine, 0.15 mM sermine, 0.3% NP40, 0.2 U µl^−1^ RNase inhibitor, ~0.07 tabs per milliliter of cOmplete protease inhibitor). Dounce with ‘A’ loose pestle (~10 strokes) and then with ‘B’ tight pestle (~10 strokes). The homogenate was filtered using a 70 µM Flowmi strainer (Bel-Art) and transferred to a pre-chilled 2-ml LoBind tube (Thermo Fisher Scientific). Nuclei were pelleted by spinning for 5 min at 4 °C and 350 relative centrifugal force (RCF). The supernatant was removed, and the nuclei were resuspended in 400 µl of 1× HB. Next, 400 µl of 50% iodixanol solution was added to the nuclei and then slowly layered with 600 µl of 30% iodixanol solution under the 25% mixture and then layered with 600 µl of 40% iodixanol solution under the 30% mixture. The nuclei were then spun for 20 min at 4 °C and 3,000 RCF in a pre-chilled swinging bucket centrifuge. Then, 200 µl of the nuclei band at the 30–40% interface was collected and transferred to a fresh tube. Next, 800 µl of 2.5% BSA in PBS plus 0.2 U µl^−1^ RNase inhibitor was added to the nuclei and then spun for 10 min at 500 RCF at 4 °C. The nuclei were resuspended with 2% BSA in PBS plus 0.2 U µl^−1^ RNase inhibitor to reach at least 500 nuclei per microliter. The nuclei were then filtered with a 40 µM Flowmi cell stainer. The nuclei were counted, and then approximately 13,000 nuclei per sample were loaded onto a 10x Genomics Next GEM Chip G. The snRNA-seq libraries were prepared using the Chromium Next GEM Single Cell 3′ Kit version 3.1 (10x Genomics) according to the manufacturer’s instructions. Libraries were sequenced on an Illumina NovaSeq 6000 sequencer at the UCSF Center for Advanced Technology (CAT) Core.

### Custom reference genome

The PS19 Tau-P301S transgenic mice carrying knocked-in human APOE isoforms/variant were used for snRNA-seq. The *Homo sapiens* microtubule-associated protein tau (MAPT) (National Center for Biotechnology Information (NCBI) reference sequence: NM_001123066.4)^83^ and the *Homo sapiens* APOE are genes of interest for this study. These genes are not expected to be a part of the mouse reference genome, so, to quantify the reads aligning to these genes of interest, a custom mouse reference genome was made using the reference mouse genome sequence (GRCm38) from Ensembl (release 98)^84^ and the mouse gene annotation file from GENCODE (release M23)^85^, similar to those used in the 10x Genomics Cell Ranger mouse reference package mm10 2020-A. The headers of the Ensembl reference mouse genome sequence FASTA file with the chromosome names were modified to match the chromosome names in a FASTA file from GENCODE. The annotation GTF file contains entries from non-poly(A) transcripts that overlap with the protein-coding genes. These reads are flagged as multi-mapped and are not counted by the 10x Genomics Cell Ranger version 7.0.0 count pipeline^86^. To avoid this, the GTF file was modified to (1) remove version suffixes from transcript, gene and exon IDs to match the Cell Ranger reference packages and (2) remove non-poly(A) transcripts. The *Homo sapiens* MAPT sequence and *Homo sapiens* APOE sequence were appended as separate chromosomes to the end of the mouse reference genome sequence, and the corresponding gene annotations were appended to the filtered mouse reference gene annotation GTF file. The 10x Genomics Cell Ranger version 7.0.0 mkref pipeline was used to build the custom reference genome using the modified FASTA and GTF file.

### Pre-processing and clustering of mouse snRNA-seq samples

The snRNA-seq samples included a total of 16 samples with four mice from each of the four genotype groups (PS19-E3, PS19-E4, PS19-E4-S/S and PS19-E4-R/S). Each group of four mice had two male and two female mice. The demultiplexed FASTQ files for these samples were aligned to the custom mouse reference genome (see custom reference genome methods for additional descriptions) using the 10x Genomics Cell Ranger version 7.0.0 count pipeline^86^, as described in the Cell Ranger documentation. The include-introns flag for the count pipeline was set to true to count the reads mapping to intronic regions. One PS19-E3 sample had a low total number of cells, and these cells had a relatively large percent of mitochondrial reads (0.1% versus 0.04% for the other samples); thus, they did not pass the quality control assessment. This sample was, therefore, excluded from all further analyses.

The filtered count matrices generated by the Cell Ranger count pipeline for 15 samples were processed using the R package for single-nucleus analysis Seurat version 4.1.1 (ref. ^87^). Each sample was pre-processed as a Seurat object, and the top 1% of cells per sample with a high number of unique genes, cells with ≤200 unique genes and cells ≥0.25% mitochondrial genes were filtered out for each sample. The 15 samples were merged into a single Seurat object, and normalization and variance stabilization was performed using sctransform^88^ with the ‘glmGamPoi’ (Bioconductor package version 1.6.0) method^89^ for initial parameter estimation.

Graph-based clustering was performed using the Seurat version 4.1.1 functions FindNeighbors and FindClusters. First, the cells were embedded in a *k*-nearest neighbor (KNN) graph (with *k* = 20) based on the Euclidean distance in the PCA space. The edge weights between two cells were further modified using Jaccard similarity. Next, clustering was performed using the Louvain algorithm implementation in the FindClusters Seurat function. Clustering with 15 principal components (PCs) and 0.7 resolution resulted in 38 distinct biologically relevant clusters, which were used for further analyses. The Seurat version 4.1.1 function RidgePlot was used to create a ridge plot showing the normalized expression of human APOE across all 38 clusters grouped by the clusters and APOE genotypes within each cluster.

### Cell type assignment

Data visualization using Seurat version 4.1.1 in the UMAP space for the 15 samples revealed no batch effects by age, sex, genotype, date of birth or nuclear isolation date. The marker genes for each cluster were identified using the FindAllMarkers Seurat function on the SCT assay data. This algorithm uses the Wilcoxon rank-sum test to iteratively identify DE genes in a cluster against all the other clusters. Marker genes were filtered to keep only positively expressed genes, detected in at least 25% of the cells in either population and with at least 0.5 log_2_ fold change. We assigned identities to cell clusters by matching the cell clusters to known cell types with the expression of canonical cell-type-specific genes, the expression of genes identified in publicly available mouse hippocampal single-cell RNA-seq datasets and the expression of each cluster’s marker genes in a publicly available resource of brain-wide in situ hybridization images, as we reported previously^22^.

### Subclustering of astrocytic and microglial snRNA-seq data

The hippocampal cell clusters 13 and 36 were annotated as the astrocyte cells, and hippocampal cell clusters 17 and 19 were annotated as the microglial cells. Both of these cell types were further subclustered. Normalization and variance stabilization was performed using sctransform^88^ with the ‘glmGamPoi’ (Bioconductor package version 1.6.0) method^89^ for initial parameter estimation. Graph-based clustering was performed using the Seurat version 4.1.1 functions FindNeighbors and FindClusters. First, the cells were embedded in a KNN graph (with *k* = 20) based on the Euclidean distance in the PCA space. The edge weights between two cells were further modified using Jaccard similarity. Next, clustering was performed using the Louvain algorithm implementation in the FindClusters Seurat function. Subclustering with 15 PCs and 0.9 resolution resulted in 12 distinct biologically relevant subclusters for astrocytes. Subclustering with 15 PCs and 0.9 resolution resulted in 15 distinct biologically relevant microglia subclusters.

### Gene set enrichment analysis

DE genes between clusters of interest were identified using the FindMarkers Seurat function on the SCT assay data. This algorithm uses the Wilcoxon rank-sum test to identify DE genes between two populations. DE genes were limited to genes detected in at least 10% of the cells in either population and with at least 0.1 log_2_ fold change. Volcano plots with log_2_ fold change and *P* value from the DE gene lists were generated using the EnhancedVolcano R package version 1.14.0. Overrepresentation (or enrichment) analysis was performed using clusterProfiler version 4.2.2 (ref. ^90^) to find gene sets of size at least 10 genes in the KEGG database^91^ for mouse associated with the DE genes. The *P* values are based on a hypergeometric test and are adjusted for multiple testing using the Benjamini–Hochberg method^92^. The same method was used for gene set enrichment analysis of astrocyte subclusters and microglia subclusters.

### Association between clusters and genotype

A GLMM_AM was implemented in the lme4 (version 1.1-30) R package^93^ and used to estimate the associations between cluster membership and the mouse model. These models were run separately for each cluster of cells. The GLM model was performed with the family argument set to the binomial probability distribution and with the ‘nAGQ’ parameter set to 10 corresponding to the number of points per axis for evaluating the adaptive Gauss–Hermite approximation for the log-likelihood estimation. Cluster membership of cells by sample was modeled as a response variable by a two-dimensional vector representing the number of cells from the given sample belonging to and not belonging to the cluster under consideration. The corresponding mouse ID from which the cell was derived was the random effect variable, and the animal model for this mouse ID was included as the fixed variable. The reference animal model was set to PS19-E4. The resulting *P* values for the estimated LOR across the four animal models (with respect to the PS19-E4) and clusters were adjusted for multiple testing using the Benjamini–Hochberg method^92^. The same method was used for estimating the between-cluster association with genotype for astrocyte subclusters and microglia subclusters.

### Association between proportion of cell types and histopathological parameters

A GLMM_histopathology was implemented in the lme4 (version 1.1-27.1) R package^93^ and used to identify cell types whose proportions are significantly associated with changes in histopathology across the samples. These models were performed separately for each combination of the cluster of cells and the six histological parameters: the hippocampal volume (mm^3^), the percent of AT8 coverage area, the percent of Iba1 coverage area, the percent of CD68 coverage area, the percent of GFAP coverage area and the percent of S100β coverage area. The GLM model was performed with the family argument set to the binomial probability distribution family and with the ‘nAGQ’ parameter set to 1 corresponding to a Laplace approximation for the log-likelihood estimation. Cluster membership of cells by sample was modeled as a response variable by a two-dimensional vector representing the number of cells from the given sample belonging to and not belonging to the cluster under consideration. The corresponding mouse model from which the cell was derived was included as a random effect, and, further, the mouse ID within the given mouse model was modeled as a random effect as well. Note, this represents the hierarchical nature of these data for the GLMM, and the mouse models are first assumed to be sampled from a ‘universe’ of mouse models; this is then followed by sampling mice within each mouse model. The modeling choice of including the mouse model as a random effect as opposed to a fixed effect is meant to increase the degrees of freedom (or maximize the statistical power) to detect the association of interest, particularly in light of the relatively small number of replicates (3–4) per animal model. The histological parameter under consideration was modeled as a fixed effect in this model.

We selected a subset of cell types of interest and visualized the LOR estimates (derived from the GLMM fits) in a heat map using pheatmap package 1.0.12 after adjusting the *P* values distribution across histopathological parameters across cell types with Benjamini–Hochberg multiple testing correction^92^. We applied the pipeline to the astrocyte and microglia subtypes and visualized the associations between astrocyte and microglia subtypes of interest and the histopathological parameters. We estimated the first five PC coordinates using the six LORs for unit change of the histopathological parameters for each of the cell types and astrocyte and microglia sub-cell types of interest. This was implemented using prcomp(scale = T, center = T) in the ‘stats’ R package. We visualized the first two PCs using fviz_pca_ind() implemented in the ‘factoextra’ 1.0.7 R package.

### General statistics and reproducibility

Sample sizes for PS19-E mouse and hiPSC immunohistochemical, flow cytometry and biochemical studies were chosen on the basis of estimates to provide statistical power of ≥80% and alpha of 0.05 based on preliminary data. Unless explicitly stated, all mouse and cell culture data are shown as mean ± s.e.m. Data distribution was assumed to be normal, but this was not formally tested. When the variances between groups in a particular measurement were unequal as shown by Bartlett’s test, Welch’s one-way ANOVA with Dunnett’s T3 test was used for multiple comparisons^94–96^. Otherwise, ordinary one-way ANOVA with Tukey’s test was used for multiple comparisons. For group-wise statistics on hiPSC-derived neurons measuring the effects of both genotype and heparin treatment, two-way ANOVA with Tukey’s post hoc test was used for multiple comparisons. Differences between only two groups were determined with the unpaired two-sided *t*-test with Welch’s correction. *P* < 0.05 was considered to be significant, and all significant *P* values were included in figures or noted in figure legends. Statistical significance was calculated with GraphPad Prism 9 for Mac (GraphPad Software). No randomization method was used for the assignment of mice or cells to study groups, and no animals or data points were excluded from these studies.

Sample sizes for mouse snRNA-seq studies were determined by a power analysis based on effect sizes from our previous studies^22,55^ and literature. Each mouse used in the snRNA-seq study underwent extensive pathological characterization. We selected two male and two female mice for each genotype group that represented the quantified average for pathological parameters. As a result of the variability in pathology in PS19-E4 and PS19-E4-R/S groups, we specifically chose mice whose quantified pathological parameters spanned the range of pathological measurements. This allowed us to correlate pathologies with snRNA-seq data. Nuclei were isolated from four mice per genotype to ensure *n* ≥ 3 mice per group. One PS19-E3 sample failed our pre-processing quality control assessment as described above and was, therefore, excluded. Investigators were not blinded during snRNA-seq analysis as sample metadata were needed to conduct comparisons.

Studies on hiPSC-derived neurons were performed with biological replicates over two or three independent rounds of differentiations. Studies using mouse tissue were performed using one cohort of mice.

### Reporting summary

Further information on research design is available in the Nature Portfolio Reporting Summary linked to this article.

## Online content

Any methods, additional references, Nature Portfolio reporting summaries, source data, extended data, supplementary information, acknowledgements, peer review information; details of author contributions and competing interests; and statements of data and code availability are available at 10.1038/s41593-023-01480-8.

### Supplementary information

Supplementary Information Supplementary Figs. 1–9 and the titles and statistical information of Supplementary Tables 1–6

Reporting Summary

Supplementary Table 1 snRNA-seq data related to all cell clusters, including marker genes, LORs of cell clusters across genotypes and treatments and cell counts per sample per cluster. The marker genes were calculated using the two-sided Wilcoxon rank-sum test as implemented in the FindAllMarkers function of Seurat. A GLMM was used to assess association with animal models and report the log odds values and unadjusted (two-sided) *P* values.

Supplementary Table 2 LOR estimates of associations with histopathology for all cell clusters

Supplementary Table 3 snRNA-seq data related to astrocyte subclusters, including LORs of subclusters across genotypes, cell counts per sample per cluster and DE genes. A GLMM was used to assess association with animal models and report the log odds values and unadjusted (two-sided) *P* values. The DE genes were calculated using the two-sided Wilcoxon rank-sum test as implemented in the FindAllMarkers function of Seurat.

Supplementary Table 4 LOR estimates of associations with histopathology for all astrocyte subclusters

Supplementary Table 5 snRNA-seq data related to microglia subclusters, including LORs of subclusters across genotypes, cell counts per sample per cluster and DE genes and pathways. A GLMM was used to assess association with animal models and report the log odds values and unadjusted (two-sided) *P* values. The DE genes were calculated using the two-sided Wilcoxon rank-sum test as implemented in the FindAllMarkers function of Seurat. KEGG pathway enrichment analysis was performed, and *P* values were based on a two-sided hypergeometric test and were adjusted for multiple testing using the Benjamini–Hochberg method.

Supplementary Table 6 LOR estimates of associations with histopathology for all microglia subclusters

Supplementary Data Supplementary Data 1. Statistical source data for Supplementary Figs. 1–6 and 8

### Source data

Source Data Fig. 1 Statistical source data for Fig. 1c,e,f,h

Source Data Fig. 1 Uncropped scans of WB gel image source data for Fig. 1d

Source Data Fig. 2 Statistical source data for Fig. 2b,c,d,f,g

Source Data Fig. 2 Uncropped scans of WB gel image source data for Fig. 2a

Source Data Fig. 3 Statistical source data for Fig. 3b,c,f,i

Source Data Fig. 3 Uncropped scans of WB gel image source data for Fig. 3e,h

Source Data Fig. 4 Statistical source data for Fig. 4,b–i,k

Source Data Fig. 5 Statistical source data for Fig. 5b,d–l,n,p–x

Source Data Fig. 7 Statistical source data for Fig. 7j,l

Source Data Fig. 8 Statistical source data for Fig. 8h

Source Data Extended Data Fig. 2 Statistical source data for Extended Data Fig. 2e,f,g,h,I,j.

Source Data Extended Data Fig. 4 Statistical source data for Extended Data Fig. 4a

Source Data Extended Data Fig. 5 Statistical source data for Extended Data Fig. 5a–t

Source Data Extended Data Fig. 7 Statistical source data for Extended Data Fig. 7k

Source Data Extended Data Fig. 9 Statistical source data for Extended Data Fig. 9b,d

## Data Availability

The mouse snRNA-seq datasets generated during the study are available in the Gene Expression Omnibus under accession number GSE217854. Data associated with Figs. 6, 7 and 8, Extended Data Figs. 6, 7 and 8 and Supplementary Fig. 7 are also available in the Supplementary Information. The mouse and hiPSC lines generated in this study are accessible upon reasonable request to the corresponding author. Source data are provided with this paper.

## Code availability

All codes generated with custom R and shell scripting during this study are accessible on GitHub at https://github.com/gladstone-institutes/YH_MN01_NatureNeuroscience_paper and on Zenodo at 10.5281/zenodo.8368715.
