# Supplementary material for: The APOE-R136S mutation protects against APOE4-driven Tau pathology, neurodegeneration and neuroinflammation
Source: Nat Neurosci. 2023 Nov 13;26(12):2104–21. doi: 10.1038/s41593-023-01480-8 (PMC10689245; doi:10.1038/s41593-023-01480-8)

nature neuroscience

Article

https://doi.org/10.1038/s41593-023-01480-8

The APOE-R136S mutation protects against APOE4-driven Tau pathology, neurodegeneration and neuroinflammation

In the format provided by the authors and unedited

Supplementary Information (Nelson M. et al.)

Table of Contents:

Supplementary Figure 1.	Number of AT8+ soma in CA1 subregion of the hippocampus and their correlations with hippocampal volume in 10-month-old mice.
Supplementary Figure 2.	Characterization of hiPSC-derived neuronal cultures with different APOE genotypes.
Supplementary Figure 3.	Levels of APOE and p-Tau in neuronal cultures derived from individual hiPSC lines with different APOE genotypes.
Supplementary Figure 4.	Western blot analysis of APOE protein levels in hippocampal lysates from 10-month-old PS19 mice with different APOE genotypes.
Supplementary Figure 5.	APOE4-R136S gene dose negatively correlates with glial cell numbers in the hippocampus.
Supplementary Figure 6.	Correlations between AT8 coverage area and glia number in tauopathy mice with different APOE genotypes.
Supplementary Figure 7.	Ridge plots of APOE expression in each hippocampal cell cluster across APOE genotypes.
Supplementary Figure 8.	Analysis of lysosomal size in astrocytes and microglia in PS19 mice with different APOE genotypes.
Supplementary Figure 9.	Diagram summarizing the dose-dependent protective effects of the R136S mutation on APOE4-driven AD pathologies.
Supplementary Table 1.	snRNA-seq data related to all cell clusters, including marker genes, log odds ratios of cell clusters across genotypes and treatments, and cell counts per sample per cluster, as an .xlsx file. The marker genes were calculated using the two-sided Wilcoxon rank sum test as implemented in the FindAllMarkers function of Seurat. A Generalized Linear Mixed-Effects Model was used to assess association with Animal Models and report the log odds values and unadjusted (two-sided) p-values.
Supplementary Table 2.	Log odd ratio estimates of associations with histopathology for all cell clusters, as an .xlsx file.
Supplementary Table 3.	snRNA-seq data related to astrocyte subclusters, including log odds ratios of subclusters across genotypes, cell counts per sample per cluster, and differentially expressed genes, as an .xlsx file. A Generalized Linear Mixed-Effects Model was used to assess association with Animal Models and report the log odds values and unadjusted (two-sided) p-values. The differentially expressed genes were calculated using the two-sided Wilcoxon rank sum test as implemented in the FindAllMarkers function of Seurat.
Supplementary Table 4.	Log odd ratio estimates of associations with histopathology for all astrocyte subclusters, as an .xlsx file.
Supplementary Table 5.	snRNA-seq data related to microglia subclusters, including log odds ratios of subclusters across genotypes, cell counts per sample per cluster, and differentially expressed genes and pathways, as an .xlsx file. A Generalized Linear Mixed-Effects Model was used to assess association with Animal Models and report the log odds values and unadjusted (two-sided) p-values. The differentially expressed genes were calculated using the two-sided Wilcoxon rank sum test as implemented in the FindAllMarkers function of Seurat. KEGG pathway enrichment analysis was performed and p-values were based on a two-sided hypergeometric test and are adjusted for multiple testing using the Benjamini-Hochberg method.
Supplementary Table 6.	Log odd ratio estimates of associations with histopathology for all microglia subclusters, as an .xlsx file.

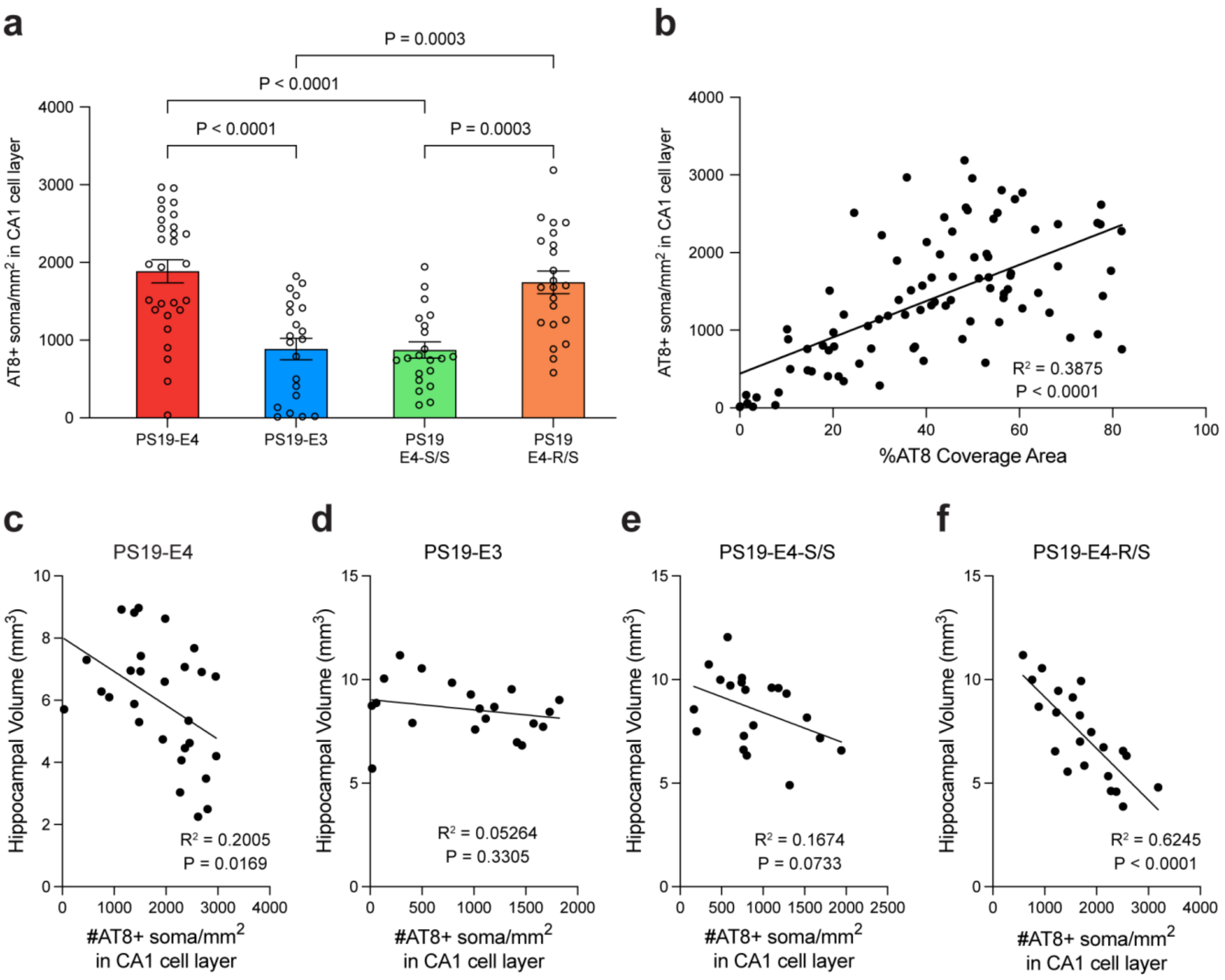

Supplementary Figure 1. Number of AT8+ soma in CA1 subregion of the hippocampus and their correlations with hippocampal volume in 10-month-old mice. a, Quantification of the AT8+ cell soma in the hippocampal subregion of the CA1 cell layer in 10-month-old PS19-E4, PS19-E3, PS19-E4-S/S, and PS19-E4-R/S mice (PS19-E4, n=28; PS19-E3, n=21; PS19-E4-S/S, n=21; PS19-E4-R/S, n=22). **b**, Correlation between AT8+ soma numbers in CA1 and % AT8 coverage area (n=90, all APOE genotype groups combined). **c**–**f**, Correlations between AT8+ soma numbers in CA1 and hippocampal volume in PS19-E4 (n=28) (c), PS19-E3 (n=21) (d), PS19-E4-S/S (n=20) (e), and PS19-E4-R/S (n=22) (f) mice. Throughout, data are expressed as mean ± s.e.m. except for correlation plots. Differences between groups were determined by ordinary one-way ANOVA followed with Tukey's multiple comparison test in a; comparisons of $p \leq 0.05$ were labeled on graph. Pearson's correlation analysis (two-sided).

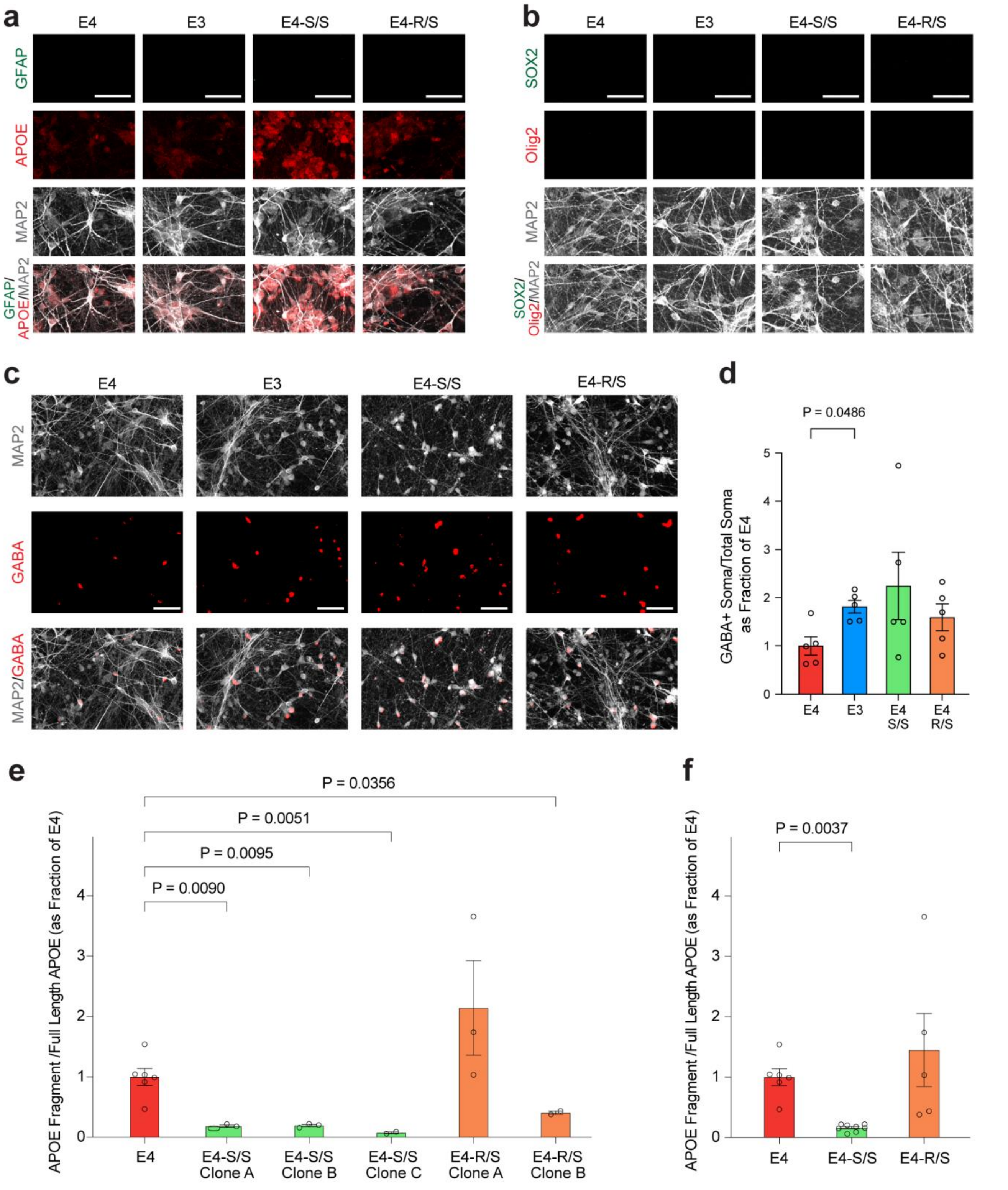

Supplementary Figure 2. Characterization of hiPSC-derived neuronal cultures with different APOE genotypes. **a**, Representative images showing APOE+ MAP2+ neurons and the absence of astrocytes as measured by GFAP in hiPSC-derived E4, E3, E4-S/S, E4-R/S neuronal cultures. **b**, Representative images showing the absence of neural progenitors as measured by SOX2 and oligodendrocytes as measured by Olig2 in hiPSC-derived E4, E3, E4-S/S, E4-R/S neuronal cultures. **c**, Representative images showing inhibitory neurons as stained by GABA and MAP2 in hiPSC-derived E4, E3, E4-S/S, or

E4-R/S neuronal cultures. **d**, Quantification of GABA+ MAP2+ soma per total MAP2+ soma. Data were normalized to E4 neurons (E4, n=5; E3, n=5; E4-S/S, n=5; E4-R/S-B, n=5; n=fields of view). **e**, Quantification of fraction of APOE fragment levels per full-length APOE in neuronal lysates derived from individual hiPSC lines with different APOE genotypes. Values were normalized to E4. (E4, n=4; E4-S/S-A, n=3; E4-S/S-B, n=3; E4-S/S-C, n=2; E4-R/S-A, n=3; E4-R/S-B, n=2). **f**, Quantification of fraction of APOE fragment levels per full-length APOE in neuronal lysates derived from each cell line grouped by genotype. Values were normalized to E4. (E4, n=4; E4-S/S, n=8; E4-R/S, n=5). Scale bars are 50μm in a, b, and c. In e,f, n=biological replicates. Representative images were selected from n=3 fields of view for each cell line. In d–f, data are expressed as mean ± s.e.m. and differences between groups were determined by Welch's ANOVA followed with Dunnett T3 multiple comparison test; comparisons of $p \leq 0.05$ were labeled on graph. FL, full-length.

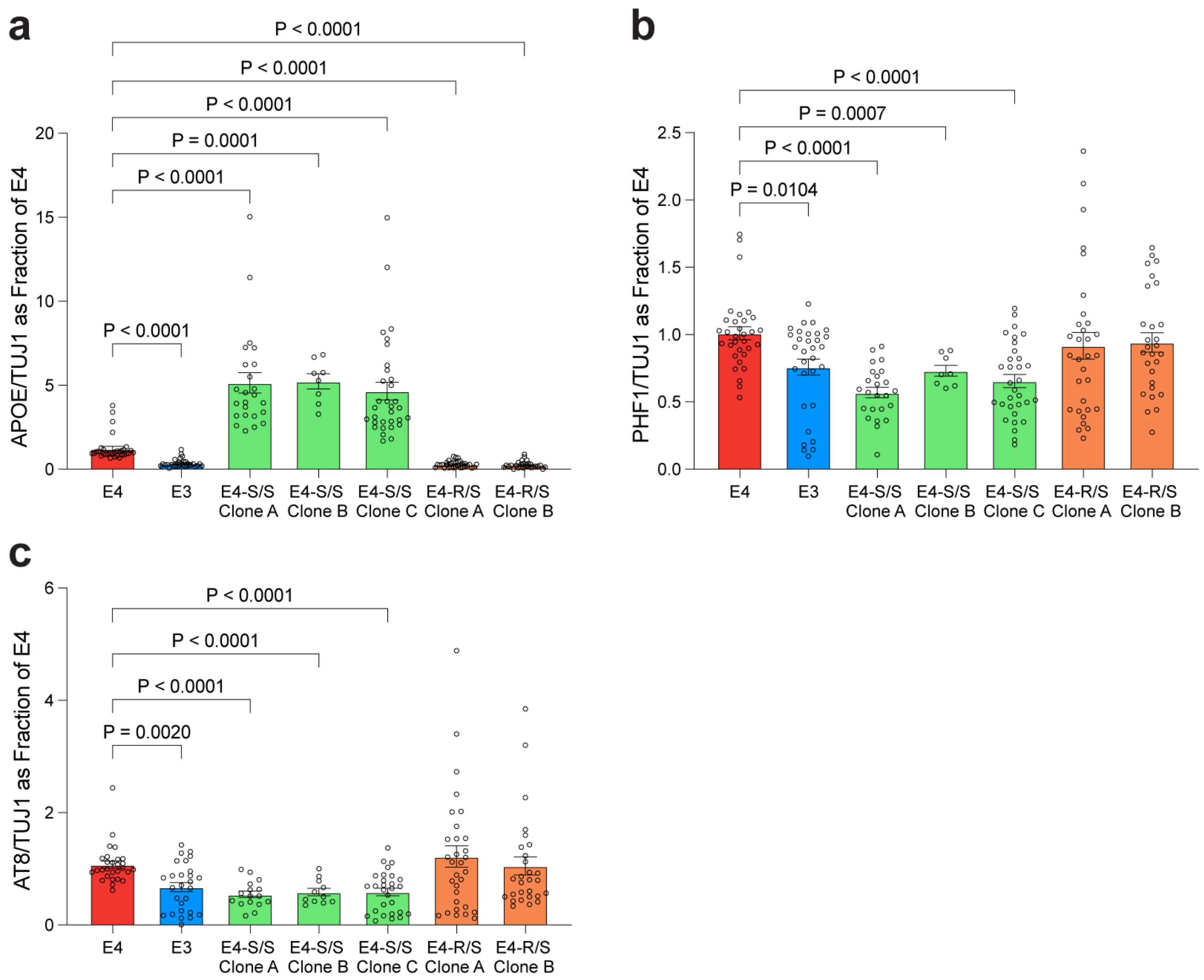

Supplementary Figure 3. Levels of APOE and p-Tau in neuronal cultures derived from individual hiPSC lines with different APOE genotypes. a, Quantification of APOE levels in neuronal lysates derived from individual hiPSC lines with different APOE genotypes. Values were normalized to E4. TUJ1 was used as loading control (E4, n=32; E3, n=31; E4-S/S-A, n=24; E4-S/S-B, n=8; E4-S/S-C, n=32; E4-R/S-A, n=31; E4-R/S-B, n=28). **b**, Quantification of PHF1-positive p-Tau levels in neuronal lysates derived from individual hiPSC lines with different APOE genotypes. Values were normalized to E4. TUJ1 was used as loading control (E4, n=32; E3, n=31; E4-S/S-A, n=24; E4-S/S-B, n=8; E4-S/S-C, n=32; E4-R/S-A, n=31; E4-R/S-B, n=28). **c**, Quantification of AT8-positive p-Tau levels in neuronal lysates derived from individual hiPSC lines with different APOE genotypes. Values were normalized to E4. TUJ1 was used as loading control (E4, n=28; E3, n=27; E4-S/S-A, n=16; E4-S/S-B, n=11; E4-S/S-C, n=28; E4-R/S-A, n=31; E4-R/S-B, n=28). Western blot data were made up of at least 3 independent rounds of differentiation, and all data were combined. In a–c, n=biological replicates. data are expressed as mean ± s.e.m. and differences between groups were determined by Welch's ANOVA followed with Dunnett T3 multiple comparison test; comparisons of p≤0.05 were labeled on graph.

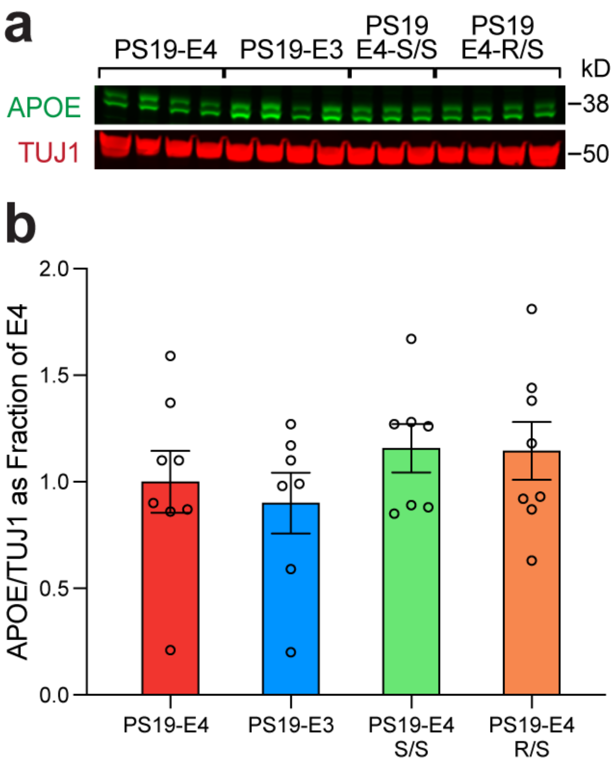

Supplementary Figure 4. Western blot analysis of APOE protein levels in hippocampal lysates from 10-month-old PS19 mice with different APOE genotypes. a, Representative western blot images of APOE. TUJ1 was used as a loading control. **b**, Quantification of APOE protein levels in hippocampal lysates of PS19-E4 (n=8), PS19-E3 (n=7), PS19-E4-S/S (n=7), and PS19-E4-R/S (n=8) mice. APOE protein levels were normalized to those of PS19-E4 mice. Throughout, data are expressed as mean ± s.e.m. Differences between groups were determined by ordinary one-way ANOVA followed with Dunnett's multiple comparison test; comparisons of $p \leq 0.05$ were labeled on graph.

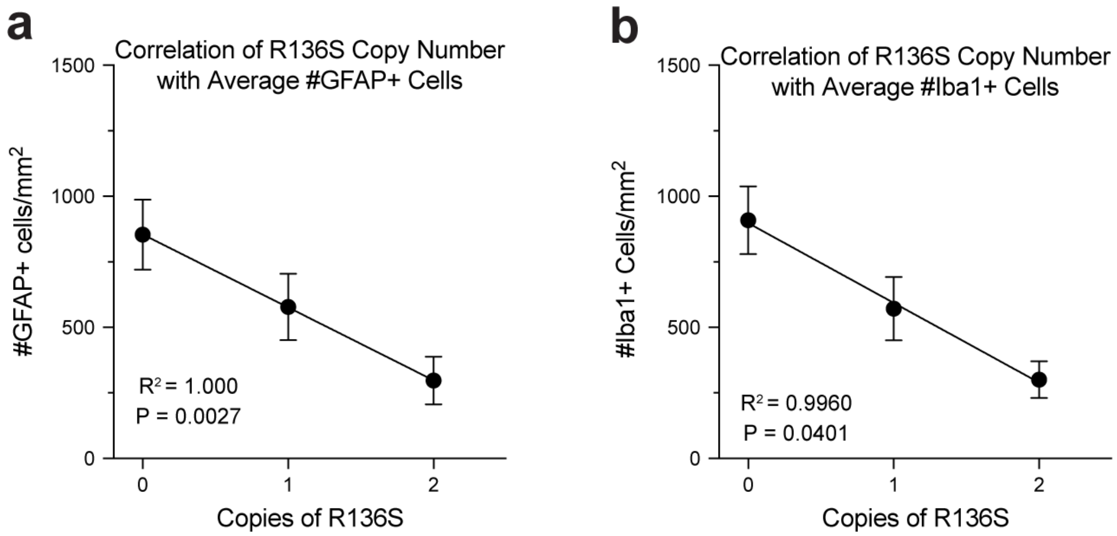

Supplementary Figure 5. APOE4-R136S gene dose negatively correlates with glial cell numbers in the hippocampus. a, Correlation of the APOE4-R136S copy number with the average of GFAP cells per mm^2 in 10-month-old PS19-E4 mice with 0, 1, 2 copies of the R136S mutation (PS19-E4, n=30; PS19-E4-R/S, n=23; PS19-E4-S/S, n=31). **b**, Correlation of the APOE4-R136S copy number with the average of Iba1 cells per mm^2 in PS19-E4 mice with 0, 1, 2 copies of the R136S mutation (PS19-E4, n=31; PS19-E4-R/S, n=23; PS19-E4-S/S, n=31). Data are expressed with 95% CI. Pearson's correlation analysis (two-sided).

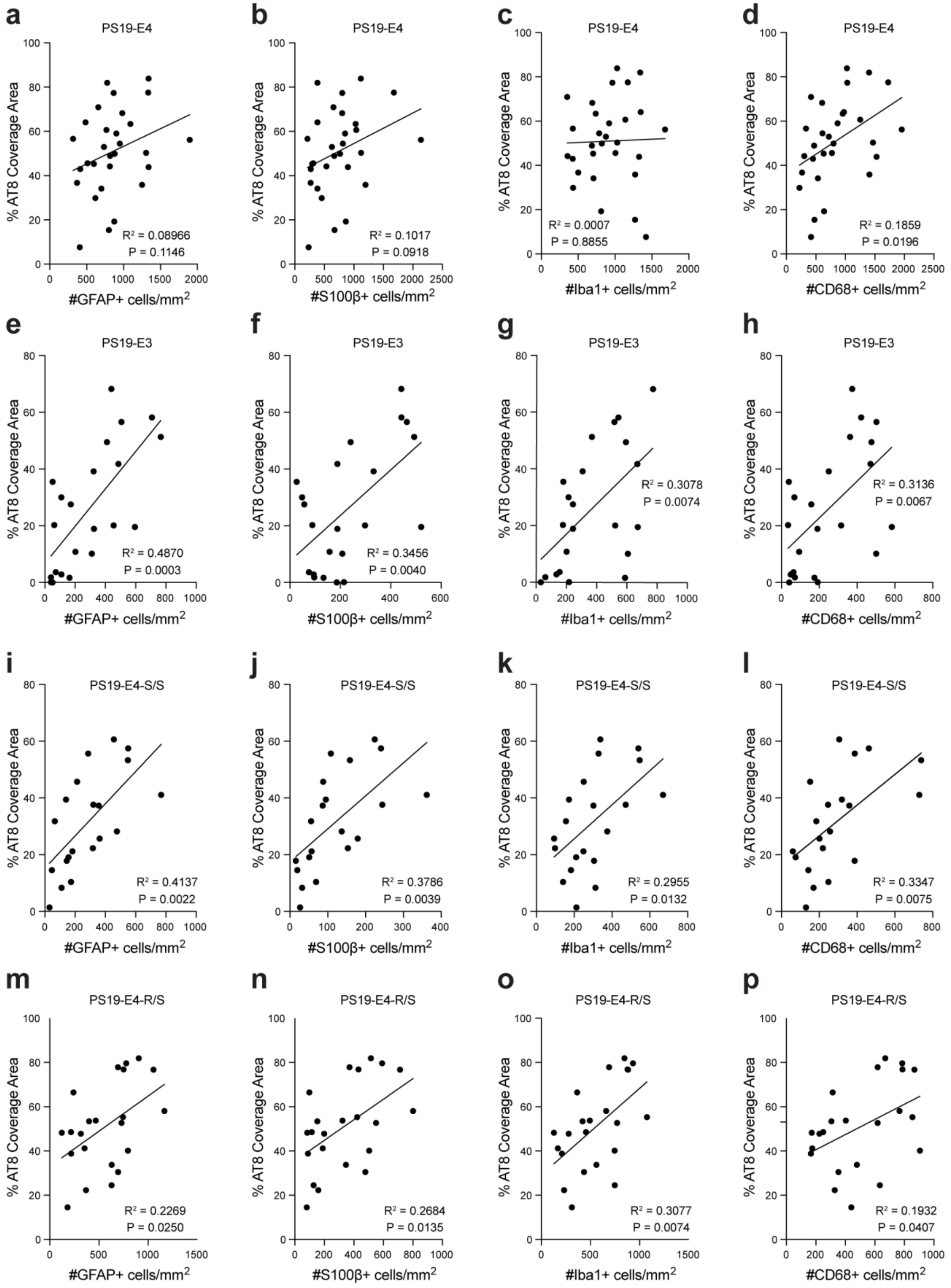

Supplementary Figure 6. Correlations between AT8 coverage area and glia number in tauopathy mice with different APOE genotypes. a–d, Correlation between % AT8 coverage area and GFAP+ cell numbers per mm^2 (a), S100β+ cell numbers per mm^2 (b), Iba1+ cell numbers per mm^2 (c), or CD68+ cell

numbers per mm^2 (d) in 10-month-old PS19-E4 mice (n=29). **e–h**, Correlation between % AT8 coverage area and GFAP+ cell numbers per mm^2 (e), S100β+ cell numbers per mm^2 (f), Iba1+ cell numbers per mm^2 (g), or CD68+ cell numbers per mm^2 (h) in PS19-E3 mice (n=22). **i–l**, Correlation between % AT8 coverage area and GFAP+ cell numbers per mm^2 (i), S100β+ cell numbers per mm^2 (j), Iba1+ cell numbers per mm^2 (k), or CD68+ cell numbers per mm^2 (l) in PS19-E4-S/S mice (n=20). **m–p**, Correlation between % AT8 coverage area and GFAP+ cell numbers per mm^2 (m), S100β+ cell numbers per mm^2 (n), Iba1+ cell numbers per mm^2 (o), or CD68+ cell numbers per mm^2 (p) in PS19-E4-R/S mice (n=22). Pearson's correlation analysis (two-sided).

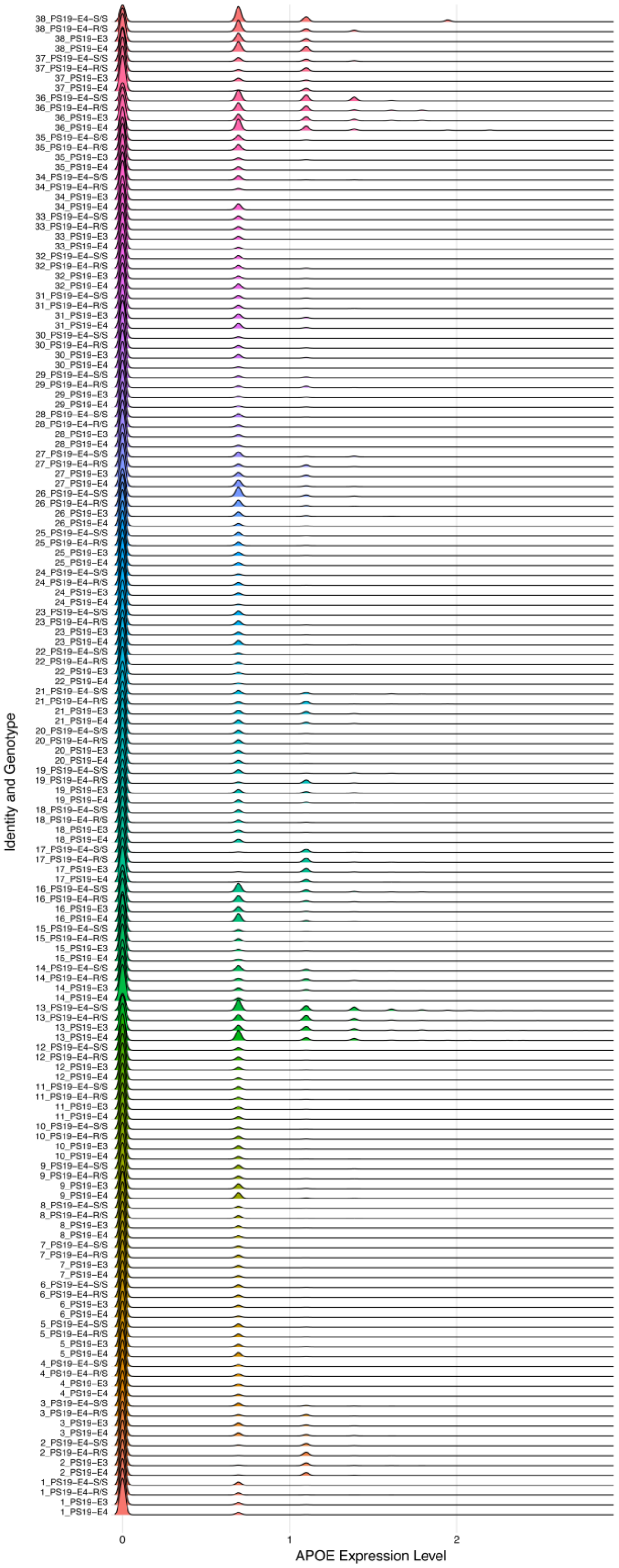

Supplementary Figure 7. Ridge plots of APOE expression in each hippocampal cell cluster across APOE genotypes. Ridge plot showing levels of normalized human APOE gene expression across all APOE genotypes within each of the 38 clusters from mice at 10 months of age. The colors represent the different clusters.

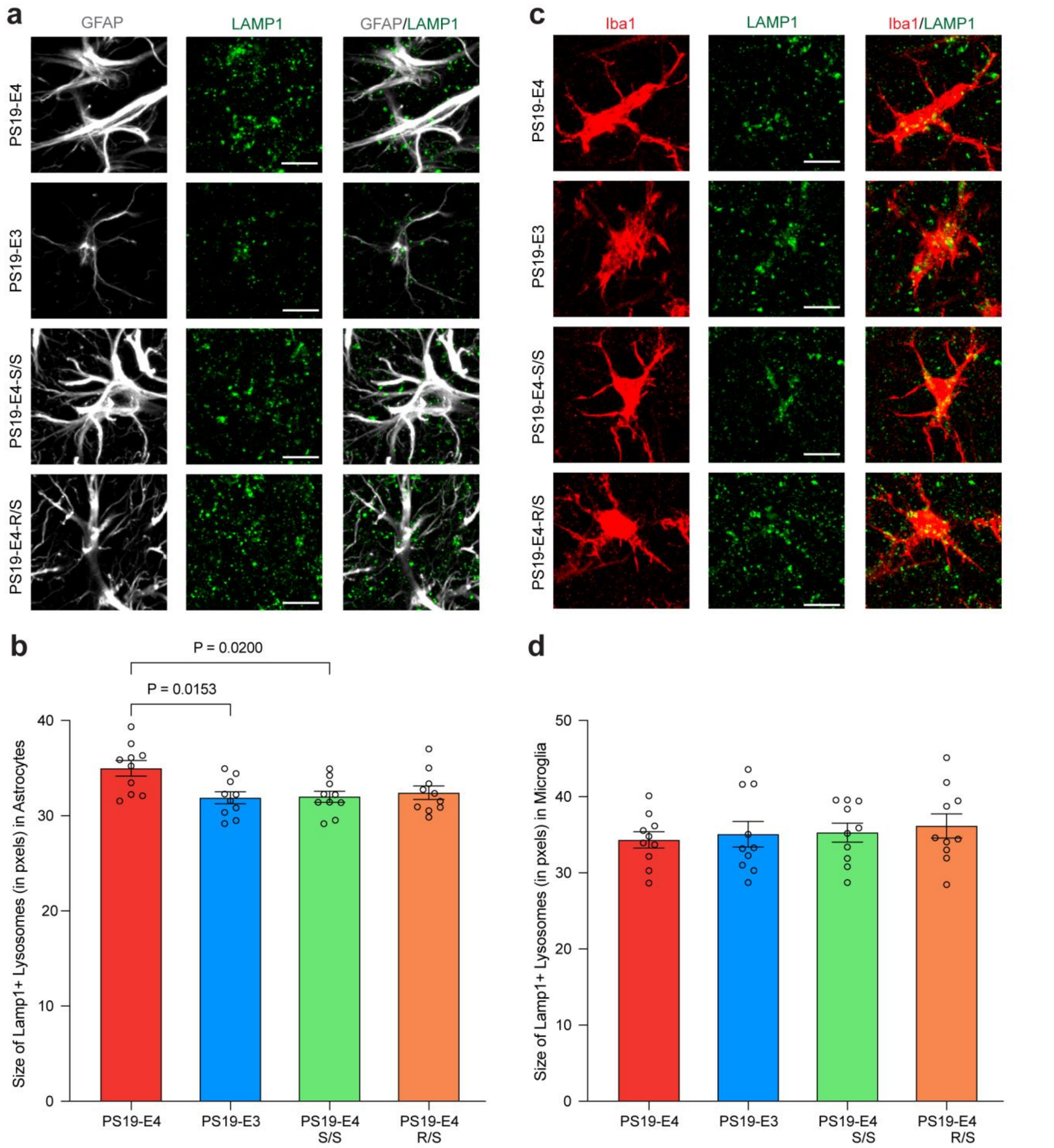

Supplementary Figure 8. Analysis of lysosomal size in astrocytes and microglia in PS19 mice with different APOE genotypes. a, Representative images of Lamp1+ immunostaining in GFAP+ astrocytes in the molecular layer of hippocampus of 10-month-old PS19-E4, PS19-E3, PS19-E4-S/S, PS19-E4-R/S mice at 10 months of age **b**, Quantification of the size (in pixels) of Lamp1+ lysosomes in GFAP+ astrocytes. **c**, Representative images of Lamp1+ immunostaining in Iba1+ microglia in the molecular layer of hippocampus of PS19-E4, PS19-E3, PS19-E4-S/S, PS19-E4-R/S mice at 10 months of age. **d,** Quantification of the size (in pixels) of Lamp1+ lysosomes in Iba1+ microglia. In b and d, n=10 per genotype. Scale bars are 10μm. Data are expressed as mean ± s.e.m. Differences between groups were determined by ordinary one-way ANOVA followed with Tukey's multiple comparison test; comparisons of $p \leq 0.05$ were labeled on graph.

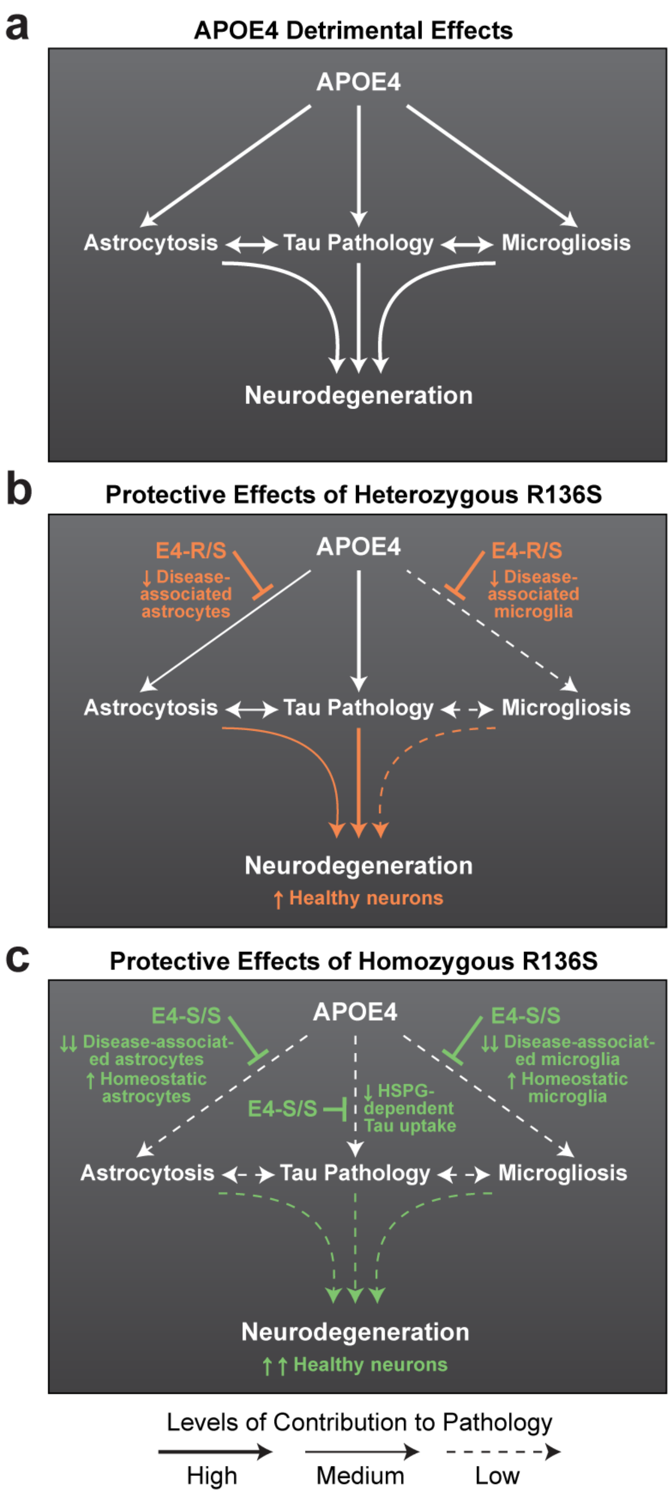

Supplementary Figure 9. Diagram summarizing the dose-dependent protective effects of the R136S mutation on APOE4-driven AD pathologies. a, APOE4 exacerbates AD pathologies, including Tau pathology, astrocytosis, microgliosis, and neurodegeneration. **b**, The heterozygous R136S mutation protects against APOE4-promoted gliosis by reducing disease-associated astrocytes and microglia, ultimately reducing neurodegeneration and increasing the population of healthy neurons. **c**, The homozygous R136S mutation protects against APOE4-promoted Tau pathology, in part due to reduced HSPG-mediated Tau uptake, and gliosis with further reduction of disease-associated astrocytes and microglia and increase in homeostatic astrocytes and microglia. This ultimately protects against neurodegeneration and leads to higher population of healthy neurons.

Source Data of Supplementary Figure 4a

a

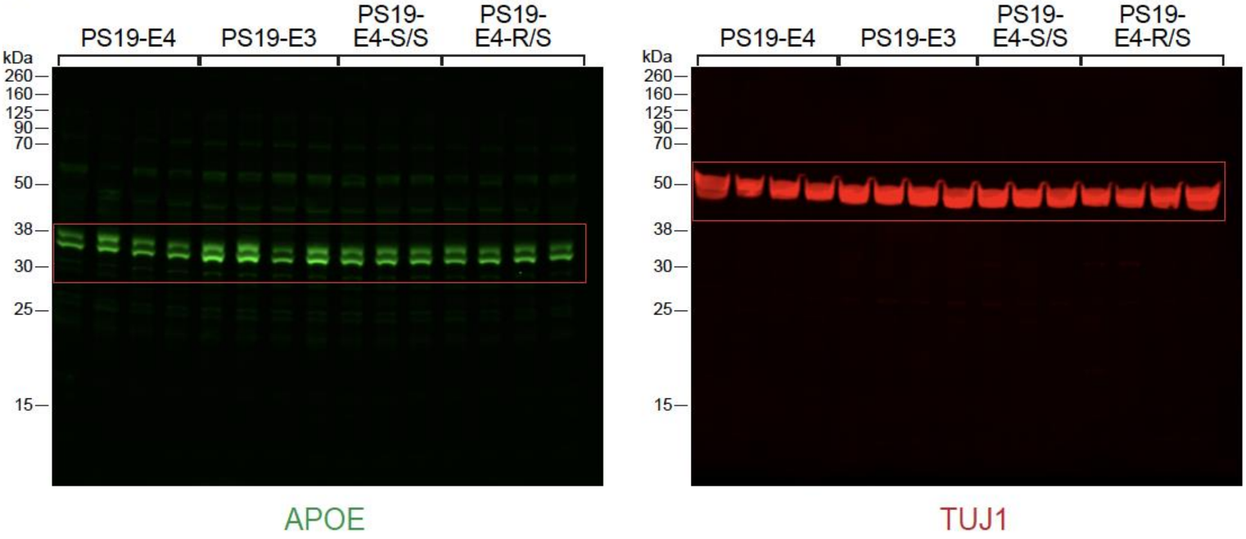

Supplement: Supplementary file 1 — Supplementary Figs. 1–9 and the titles and statistical information of Supplementary Tables 1–6 [file 41593_2023_1480_MOESM1_ESM.pdf]
