# Supplementary material for: The APOE-R136S mutation protects against APOE4-driven Tau pathology, neurodegeneration and neuroinflammation
Source: Nat Neurosci. 2023 Nov 13;26(12):2104–21. doi: 10.1038/s41593-023-01480-8 (PMC10689245; doi:10.1038/s41593-023-01480-8)
Supplement: Supplementary file 2 — Reporting Summary [file 41593_2023_1480_MOESM2_ESM.pdf]

nature research

Corresponding author(s): Yadong Huang

Last updated by author(s): 09/25/2023

Reporting Summary

Nature Research wishes to improve the reproducibility of the work that we publish. This form provides structure for consistency and transparency in reporting. For further information on Nature Research policies, see our Editorial Policies and the Editorial Policy Checklist.

Statistics

For all statistical analyses, confirm that the following items are present in the figure legend, table legend, main text, or Methods section.

n/a	Confirmed	
☐	☒	The exact sample size (*n*) for each experimental group/condition, given as a discrete number and unit of measurement
☐	☒	A statement on whether measurements were taken from distinct samples or whether the same sample was measured repeatedly
☐	☒	The statistical test(s) used AND whether they are one- or two-sided<br>*Only common tests should be described solely by name; describe more complex techniques in the Methods section.*
☐	☒	A description of all covariates tested
☐	☒	A description of any assumptions or corrections, such as tests of normality and adjustment for multiple comparisons
☐	☒	A full description of the statistical parameters including central tendency (e.g. means) or other basic estimates (e.g. regression coefficient) AND variation (e.g. standard deviation) or associated estimates of uncertainty (e.g. confidence intervals)
☐	☒	For null hypothesis testing, the test statistic (e.g. *F*, *t*, *r*) with confidence intervals, effect sizes, degrees of freedom and *P* value noted<br>*Give P values as exact values whenever suitable.*
☒	☐	For Bayesian analysis, information on the choice of priors and Markov chain Monte Carlo settings
☒	☐	For hierarchical and complex designs, identification of the appropriate level for tests and full reporting of outcomes
☐	☒	Estimates of effect sizes (e.g. Cohen's *d*, Pearson's *r*), indicating how they were calculated

Our web collection on statistics for biologists contains articles on many of the points above.

Software and code

Policy information about availability of computer code

Data collection	No software was used in the collection of data for this study, aside from the programs for microscopes, flow cytometers (BD FACSDiva Software v9.2), Western blot image capture, and sequencing machines. The specifications for those hardware are described in the Methods section.
Data analysis	Analyses were conducted using the following publicly-available software packages: Seurat v4.1.1, Cell Ranger v6.1.1, Cellranger v7.0.0, glmGamPoi v1.6.0, clusterProfiler v4.2.2, lme4 v.1.1-27.1, lme4 v.1.1-30, factoextra v1.0.7, EnhancedVolcano R package version 1.14.0, pheatmap v1.0.12, Bioconductor package version 1.6.0, Fiji v2.3 (ImageJ), and CellProfiler v.4.2.1. Analysis of gene editing data via Sanger sequencing performed using Geneious Prime v2021.1.1. Western blot images were analyzed using Image Studio Lite v.5.2.5 (LI-COR). Flow cytometry data were analyzed using FlowJo v.10.8.0 (BD Biosciences). GraphPad Prism 9 for Mac was used for graphing and statistical analyses.<br><br>All data analysis packages are included in the Code Availability section in the Methods. Packages that were dependencies or used to make figures are also listed in the Code Availability section in the Methods.<br><br>All other data analyses were done with custom R and shell scripting that are accessible via GitHub at https://github.com/gladstone-institutes/YH_MN01_NatureNeuroscience_paper and on Zenodo at https://doi.org/10.5281/zenodo.8368715.

For manuscripts utilizing custom algorithms or software that are central to the research but not yet described in published literature, software must be made available to editors and reviewers. We strongly encourage code deposition in a community repository (e.g. GitHub). See the Nature Research guidelines for submitting code & software for further information.

Data

Policy information about availability of data

All manuscripts must include a data availability statement. This statement should provide the following information, where applicable:

- Accession codes, unique identifiers, or web links for publicly available datasets
- A list of figures that have associated raw data
- A description of any restrictions on data availability

Mouse single-nucleus RNA sequencing datasets generated in association with this study are available in the Gene Expression Omnibus (GEO) under the accession number GSE217854. Source Data associated with Figures 6, 7, 8, Extended Data Figures 6–9, and Supplementary Figure 7 are available in the Supplementary Information.

The Homo sapiens microtubule associated protein tau (MAPT) NCBI Reference Sequence: NM_001123066.4 is available at: https://www.ncbi.nlm.nih.gov/nuccore/NM_001123066

The Kyoto Encyclopedia of Genes and Genomes (KEGG) Pathways database is available at: https://www.genome.jp/kegg/pathway.html

The reference mouse genome sequence (GRCm38) from Ensembl (release 98) is available at: http://ftp.ensembl.org/pub/release-98/fasta/mus_musculus/dna/Mus_musculus.GRCm38.dna.primary_assembly.fa.gz

The reference mouse gene annotation file from GENCODE (release M23) is available at: http://ftp.ebi.ac.uk/pub/databases/gencode/Gencode_mouse/release_M23/gencode.vM23.primary_assembly.annotation.gtf.gz

Field-specific reporting

Please select the one below that is the best fit for your research. If you are not sure, read the appropriate sections before making your selection.

☒ Life sciences ☐ Behavioural & social sciences ☐ Ecological, evolutionary & environmental sciences

For a reference copy of the document with all sections, see nature.com/documents/nr-reporting-summary-flat.pdf

Life sciences study design

All studies must disclose on these points even when the disclosure is negative.

Sample size

For immunohistochemical analyses, sample sizes were determined using effect sizes estimated from pilot cohorts and previous studies. We utilized n>=20 mice per genotype group, to show genotypic effects of >20% between groups or n>=9 mice per genotype to show genotypic effects of >=50% between groups, and allow for a statistical power of >80% and two-sided alpha of 0.05.

Based on preliminary hiPSC-derived neuron data, a sample size of 8 would be sufficient to show genotypic effects of >20% between groups for immunocytochemical and biochemical analyses and allow for a statistical power of >80% and two-sided alpha of 0.05; all groups had n >= 8/cell line. Figures 2 and 3 show data combined by genotype and Extended Data Figure 3 shows data by each individual hiPSC line-derived neurons in culture. For the low conditioned media study and the Tau uptake assay using flow cytometry, preliminary studies showed drastic differences between genotype groups, so we utilized n >= 4 for each genotype and treatment condition as this was sufficient for a power of >=80%.

For single-nucleus RNA-sequencing experiments, sample sizes were determined by a power analysis using effect sizes estimated from our previous studies (Koutsodendris, N. et al. Neuronal APOE4 removal protects against tau-mediated gliosis, neurodegeneration and myelin deficits. Nat Aging 3, 275–296 (2023)) and a literature search. Nuclei were isolated from 4 mice per mouse genotype to ensure an n of >= 3 mice per genotype, resulting in a total of 16 samples. Sample preparation was successful for 15 out of 16 samples. One sample had low quality and quantity of cDNA recovery and was excluded from downstream analyses with Seurat. All other 15 samples had high quality and quantity of cDNA recovery. See methods section for more details.

Data exclusions

No data were excluded.

Replication

Western blotting, flow cytometry, and immunocytochemical experiments in hiPSC-derived neurons were replicated successfully at least twice. All Western blotting, immunohistochemical, an snRNAseq experiments were performed on one cohort of mice.

Randomization

Mice and hiPSC-derived neuronal culture wells were randomly allocated to groups for all immunostaining and biochemical studies.

For single-nucleus RNA-sequencing studies, the mice had undergone rigorous pathological characterization and we selected mice in each genotype group that represented near the quantified average for all pathological parameters for that genotype group. Due to the variability of pathology in certain genotype groups, specifically selecting mice that are good representatives of each genotype group for sequencing analysis allowed us to make more accurate correlations between pathologies and sequencing data.

Blinding

Investigators were not blinded to mouse genotype groups during data collection for immunohistochemical or Western blotting studies, but researchers were blinded during all data calculation and analyses. For hiPSC-derived neuron immunostaining and biochemical studies, investigators were not blinded during data collection, but the researchers were blinded during data calculation and analyses and/or analysis was automated to reduce bias. For hiPSC-derived neuron treatment assays, investigators were not blinded during application of identical treatments, but researchers were blinded during analysis of Western blotting data or performed automated analyses across all samples for

flow cytometry analysis.

Investigators were not blinded during analysis of the single-nucleus RNA-sequencing datasets, as sample metadata was needed to conduct any comparisons.

Reporting for specific materials, systems and methods

We require information from authors about some types of materials, experimental systems and methods used in many studies. Here, indicate whether each material, system or method listed is relevant to your study. If you are not sure if a list item applies to your research, read the appropriate section before selecting a response.

Materials & experimental systems

n/a	Involved in the study
☐	☒ Antibodies
☐	☒ Eukaryotic cell lines
☒	☐ Palaeontology and archaeology
☐	☒ Animals and other organisms
☒	☐ Human research participants
☒	☐ Clinical data
☒	☐ Dual use research of concern

Methods

n/a	Involved in the study
☒	☐ ChIP-seq
☐	☒ Flow cytometry
☒	☐ MRI-based neuroimaging

Antibodies

Antibodies used

Mouse anti-AT8, Invitrogen, #MN1020
Rabbit anti-ApoE, Cell Signaling, #13366
Goat anti-ApoE, Sigma-Aldrich, #178479
BODIPY, Thermo Fisher, #D3922
Rat anti-CD68, Bio-Rad, #MCA1957
Rabbit anti-GABA, Sigma, #A2052
Rabbit anti-GFAP, Agilent, #Z0334
Mouse anti-GFAP, MilliporeSigma, #MAB3402
Rat anti-GFAP, Thermo Fisher, #13-0300
Rabbit anti-Gpnmb, Cell Signaling, #56898S
Goat anti-Iba1, Abcam, #ab5076
Rabbit anti-Iba1, Wako, #019-19741
Mouse anti-Id3, Thermo Fisher, #PA5-100268
Rabbit anti-Kirrel3, Thermo Fisher, #PA5-63287
Rat anti-LAMP1, Thermo Fisher, #14-1071-82
Chicken anti-MAP2, Thermo Fisher, #PA1-10005
Rabbit anti-MAP2, EMD Millipore, #AB5622-I
Rabbit anti-NANOG, Stemgent, #09-0020
Guinea Pig anti-NeuN, MilliporeSigma, #ABN90
Rabbit anti-Nkain2, VWR, #ABGEAP16664C
Rabbit anti-NuMa, Abcam, #ab84680
Mouse anti-OCT3/4, SCBT, #sc-5279
Goat anti-Olig2, R&D Systems, #AF2418
Mouse anti-PHF1, gift from Peter Davies
Mouse anti-SOX2, SCBT, #sc-365823
Rabbit anti-S100β, Abcam, #ab52642
Sheep anti-Tau, Abcam, #ab62639
Mouse anti-TRA-1-60, EMD Millipore, #MAB4360
Rabbit anti-TUJ1, Biolegend, #802001
Donkey anti-mouse Biotin-SP, Jackson Immuno, #715-065-150
DAPI, Thermo Fisher, #62248
Donkey anti-mouse Alexa Fluor 488, Invitrogen, #A-21202
Donkey anti-goat Alexa Fluor 488, Invitrogen, #A-11055
Donkey anti-rabbit Alexa Fluor 488, Invitrogen, #A-21206
Donkey anti-rat Alexa Fluor 488, Invitrogen, #A-21208
Donkey anti-mouse Alexa Fluor 594, Invitrogen, #A-21203
Donkey anti-goat Alexa Fluor 594 Invitrogen, #11058
Donkey anti-rabbit Alexa Fluor 594, Invitrogen, #A-21207
Donkey anti-guinea pig 594, Jackson Immuno, #706-585-148
Donkey anti-mouse 647, Abcam, #ab150107
Donkey anti-rabbit 647, Abcam, #ab150075
Donkey anti-guinea pig 647, Jackson Immuno, #706-605-148
Donkey anti-mouse IRDye 800CW, LI-COR, #926-32212
Donkey anti-rabbit IRDye 680RD, LI-COR, #926-68073
Donkey anti-goat IRDye 800CW, LI-COR, #926-32214

Validation

Validated for WB in mouse tissue and cell culture lysate:

Validation	Mouse anti-AT8, Invitrogen, #MN1020; Goat anti-ApoE, Sigma-Aldrich, #178479; Rabbit anti-TUJ1, Biolegend, #802001; Mouse anti-PHF1, gift from Peter Davies; Donkey anti-mouse IRDye 800CW, LI-COR, #926-32212; Donkey anti-rabbit IRDye 680RD, LI-COR, #926-68073; Donkey anti-goat IRDye 800CW, LI-COR, #926-32214<br><br>Validated for IHC in mouse tissue:<br>Mouse anti-AT8, Invitrogen, #MN1020; BODIPY, Thermo Fisher, #D3922; Rat anti-CD68, Bio-Rad, #MCA1957; Mouse anti-GFAP, MilliporeSigma, #MAB3402; Rat anti-GFAP, Thermo Fisher, #13-0300; Rabbit anti-Gpnmb, Cell Signaling, #56898S; Rabbit anti-Iba1, Wako, #019-19741; Goat anti-Iba1, Abcam, #ab5076; Mouse anti-Id3, Thermo Fisher, #PA5-100268; Rabbit anti-Kirrel3, Thermo Fisher, #PA5-63287; Rat anti-LAMP1, Thermo Fisher, #14-1071-82; Chicken anti-MAP2, Thermo Fisher, #PA1-10005; Guinea Pig anti-NeuN, MilliporeSigma, #ABN90; Rabbit anti-Nkain2, VWR, #ABGEAP16664C; Rabbit anti-S100β, Abcam, #ab52642; Donkey anti-mouse Biotin-SP, Jackson Immuno, #715-065-150<br><br>Validated for IHC in mouse tissue and ICC for cell culture:<br>Rabbit anti-ApoE, Cell Signaling, #13366; Rabbit anti-GFAP, Agilent, #Z0334; Chicken anti-MAP2, Thermo Fisher, #PA1-10005; Goat anti-Olig2, R&D Systems, #AF2418; Donkey anti-mouse Alexa Fluor 488, Invitrogen, #A-21202; Donkey anti-goat Alexa Fluor 488, Invitrogen, #A-11055; Donkey anti-rabbit Alexa Fluor 488, Invitrogen, #A-21206; Donkey anti-rat Alexa Fluor 488, Invitrogen, #A-21208; Donkey anti-mouse Alexa Fluor 594, Invitrogen, #A-21203; Donkey anti-goat Alexa Fluor 488, Invitrogen, #11058; Donkey anti-rabbit Alexa Fluor 594, Invitrogen, #A-21207; Donkey anti-guinea pig 594, Jackson Immuno, #706-585-148; Donkey anti-mouse 647, Abcam, #ab150107; Donkey anti-rabbit 647, Abcam, #ab150075; Donkey anti-guinea pig 647, Jackson Immuno, #706-605-148<br><br>Validated for ICC in cell culture:<br>Rabbit anti-GABA, Sigma, #A2052; Rabbit anti-MAP2, EMD Millipore, #AB5622-I; Rabbit anti-NANOG, Stemgent, #09-0020; Rabbit anti-NuMa, Abcam, #ab84680; Mouse anti-OCT3/4, SCBT, #sc-5279; Mouse anti-SOX2, SCBT, #sc-365823; Sheep anti-Tau, Abcam, #ab62639; Mouse anti-TRA-1-60, EMD Millipore, #MAB4360; Mouse anti-PHF1, gift from Peter Davies<br><br>Validated for IHC in mouse tissue and cell culture for flow cytometry:<br>DAPI, Thermo Fisher, #62248

Eukaryotic cell lines

Policy information about cell lines

Cell line source(s)	The APOE4 and APOE3 hiPSC lines were generated and fully characterized previously in our lab, as reported in Wang, C. et al. Gain of toxic apolipoprotein E4 effects in human iPSC-derived neurons is ameliorated by a small-molecule structure corrector. Nature Medicine 24, 647–657 (2018). The APOE4-S/S and APOE4-R/S hiPSC lines were generated and fully characterized in our lab, as described in Methods.<br><br>The APOE-KO line used in this study was generated and characterized previously in our lab, as reported in Wang, C. et al. Gain of toxic apolipoprotein E4 effects in human iPSC-derived neurons is ameliorated by a small-molecule structure corrector. Nature Medicine 24, 647–657 (2018).
Authentication	The karyotype and pluripotency of the APOE4 and APOE3 hiPSC lines were confirmed previously (Wang, C. et al. Gain of toxic apolipoprotein E4 effects in human iPSC-derived neurons is ameliorated by a small-molecule structure corrector. Nature Medicine 24, 647–657 (2018)). The karyotype and pluripotency of the APOE4-S/S and APOE4-R/S hiPSC lines were confirmed in the current study, as shown in Extended Data Fig. 2. The APOE genotype for all hiPSC lines was verified via Sanger DNA sequencing. The capability of all hiPSC lines to differentiate into neurons in culture was validated and confirmed in the current study.
Mycoplasma contamination	All hiPSC lines used in this study were tested negative for mycoplasma contamination.
Commonly misidentified lines<br>(See ICLAC register)	No commonly misidentified cell lines were used.

Animals and other organisms

Policy information about studies involving animals; ARRIVE guidelines recommended for reporting animal research

Laboratory animals	APOE3-KI mice: Apoetm2(APOE_i3)Yhg, available from Yadong Huang (Bien-Ly N, Gillespie AK, Walker D, Yoon SY, Huang Y. Reducing human apolipoprotein E levels attenuates age-dependent Aβ accumulation in mutant human amyloid precursor protein transgenic mice. J. Neurosci. 2012 Apr. 4; 32(14)4802-11).<br><br>APOE4-KI mice: Apoetm3(APOE_i4)Yhg, available from Yadong Huang (Bien-Ly N, Gillespie AK, Walker D, Yoon SY, Huang Y. Reducing human apolipoprotein E levels attenuates age-dependent Aβ accumulation in mutant human amyloid precursor protein transgenic mice. J. Neurosci. 2012 Apr. 4; 32(14)4802-11).<br><br>APOE4-S/S-KI and APOE4-R/S-KI mice: To generate APOE4-KI mice harboring the R136S mutation (E4-S/S or E4-R/S), CRISPR-Cas9-based knock-in strategy was used, as described in Methods.<br><br>APOE4-KI, APOE3-KI, APOE4-S/S-KI, and APOE4-R/S-KI mice were then cross-bred with Tau-P301S (PS19 line) (Jax #008169) mice to generate PS19-E4, PS19-E3, PS19-E4-S/S, and PS19-E4-R/S mice. Both male and female mice at 10 months old were used for pathological and transcriptomic analyses. Both female and male mice at 6 months old were analyzed for pathological analyses.<br><br>WT mice on a C57BL/6 background (Jax #000664) were bred separately from the PS19-E mice and used for control studies. Both male and female mice at 10 months old were used for pathological analyses.

Wild animals	No wild animals were used in this study.
Field-collected samples	No field-collected samples were used in this study.
Ethics oversight	All protocols and procedures followed the guidelines of the Laboratory Animal Resource Center at the University of California, San Francisco (UCSF) and the ethical approval of the UCSF IACUC.

Note that full information on the approval of the study protocol must also be provided in the manuscript.

Flow Cytometry

Plots

Confirm that:

☒ The axis labels state the marker and fluorochrome used (e.g. CD4-FITC).

☒ The axis scales are clearly visible. Include numbers along axes only for bottom left plot of group (a 'group' is an analysis of identical markers).

☒ All plots are contour plots with outliers or pseudocolor plots.

☒ A numerical value for number of cells or percentage (with statistics) is provided.

Methodology

Sample preparation	hiPSC-derived neurons with different APOE genotypes were incubated with the recombinant Tau-488 in the absence or presence of heparin. The cells were then dissociated in Accutase and stained with DAPI before analysis via flow cytometry. See Methods for more details.
Instrument	BD LSRFortessa X-20 of BD Biosciences
Software	BD FACSDiva Software for data collection, FlowJo v.10.8.0 (BD Biosciences) for data analysis, and GraphPad Prism 9 for Mac for graphing and statistical analyses.
Cell population abundance	Cells ranged from 0–80% positive for Tau-488, depending on APOE genotype and presence or absence of heparin treatment.
Gating strategy	Cells were gated on forward scatter/side scatter (FSC/SSC). Cells were then gated on forward scatter height (FSC-H) versus area (FSC-A) to discriminate doublets. Dead cells were removed from the analysis using nuclear stain with DAPI, and positive cells were determined by gating on a control (without Tau-488) population. Roughly 100,000 to 500,000 events were counted in each experiment. The live cell population was down-sampled for some samples so that each samples was roughly 5000 cells for sample for accurate comparisons.

☒ Tick this box to confirm that a figure exemplifying the gating strategy is provided in the Supplementary Information.
