# Supplementary figures and images for: The APOE-R136S mutation protects against APOE4-driven Tau pathology, neurodegeneration and neuroinflammation
Source: Nat Neurosci. 2023 Nov 13;26(12):2104–21. doi: 10.1038/s41593-023-01480-8 (PMC10689245; doi:10.1038/s41593-023-01480-8)

Figure 1

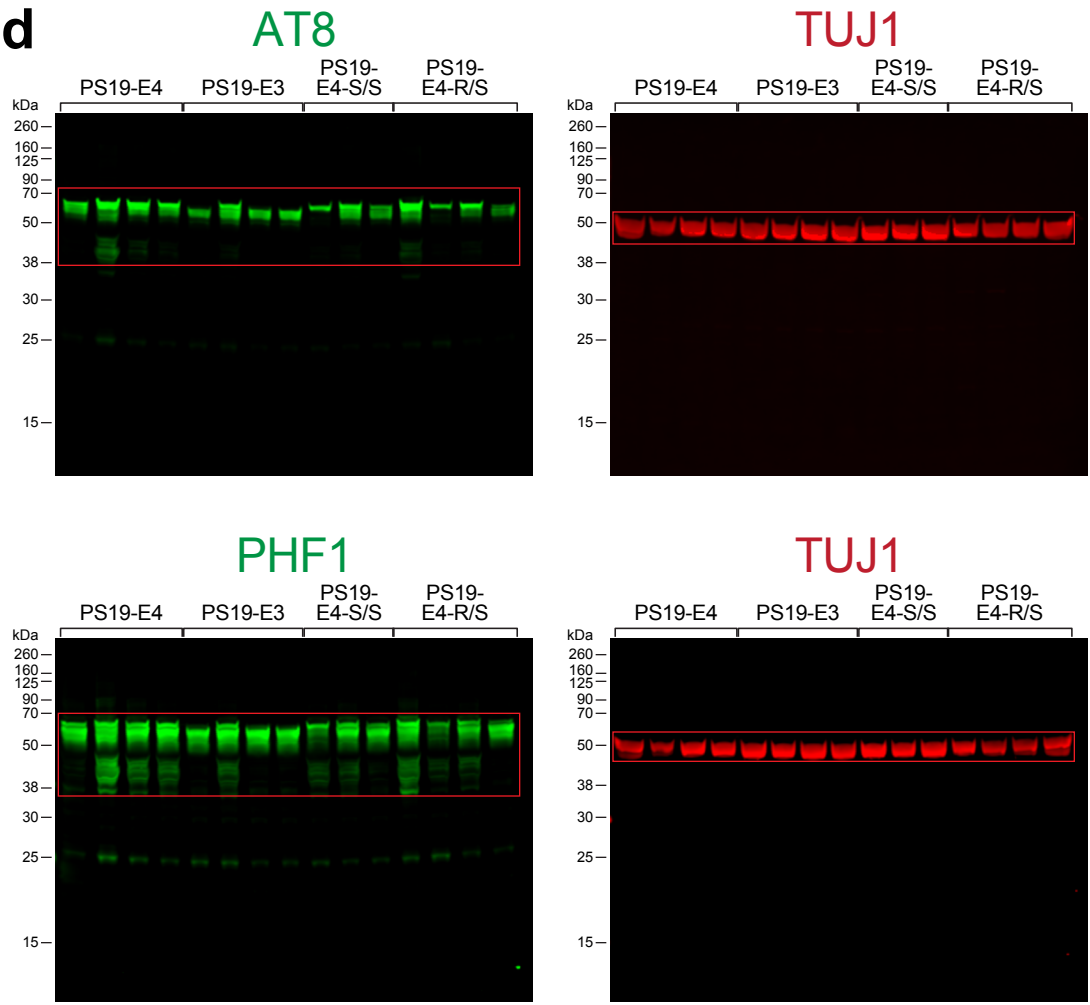

Supplement: Supplementary file 11 — Uncropped scans of WB gel image source data for Fig. 1d [file 41593_2023_1480_MOESM11_ESM.pdf]

Figure 2

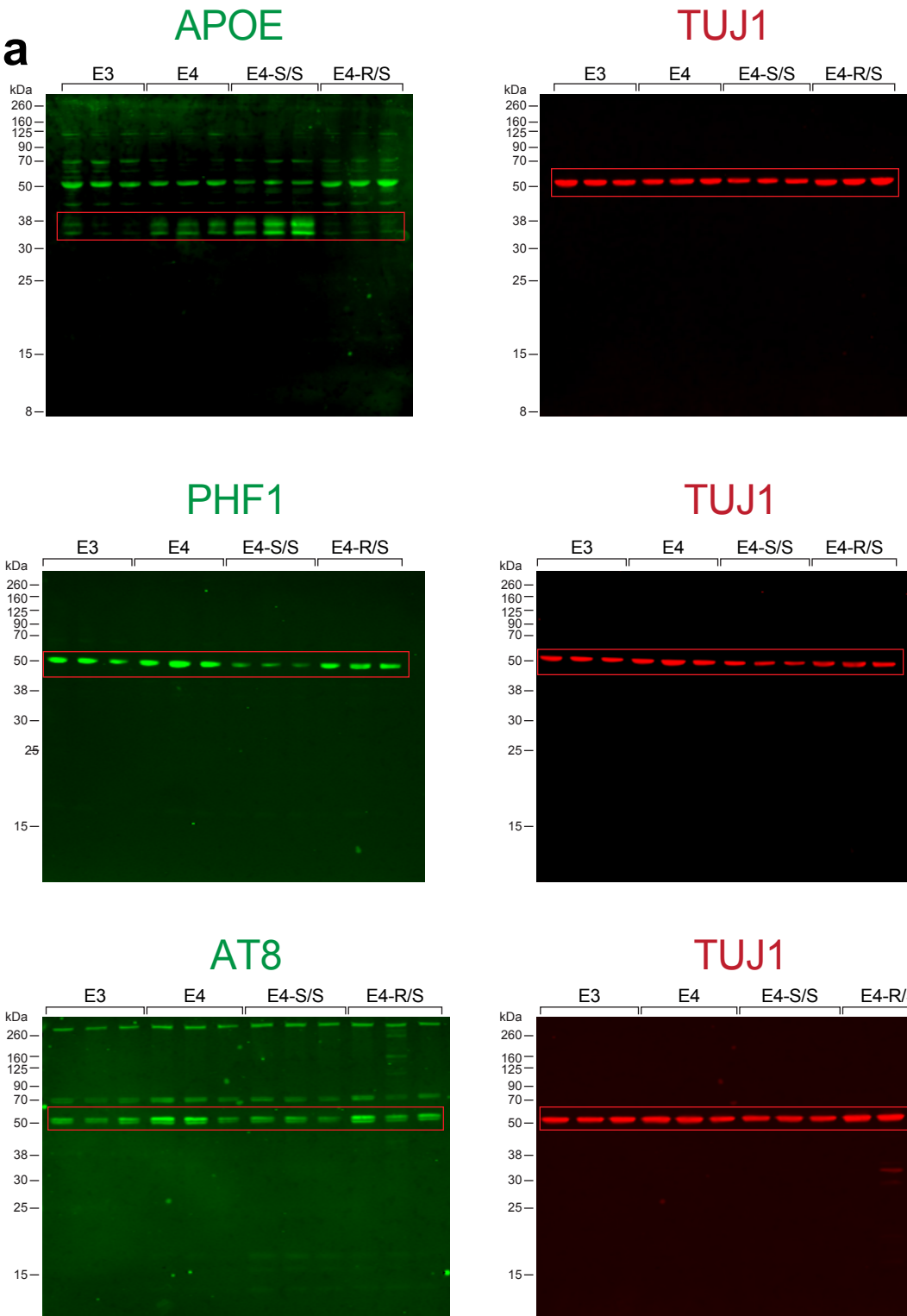

Supplement: Supplementary file 13 — Uncropped scans of WB gel image source data for Fig. 2a [file 41593_2023_1480_MOESM13_ESM.pdf]

Figure 3

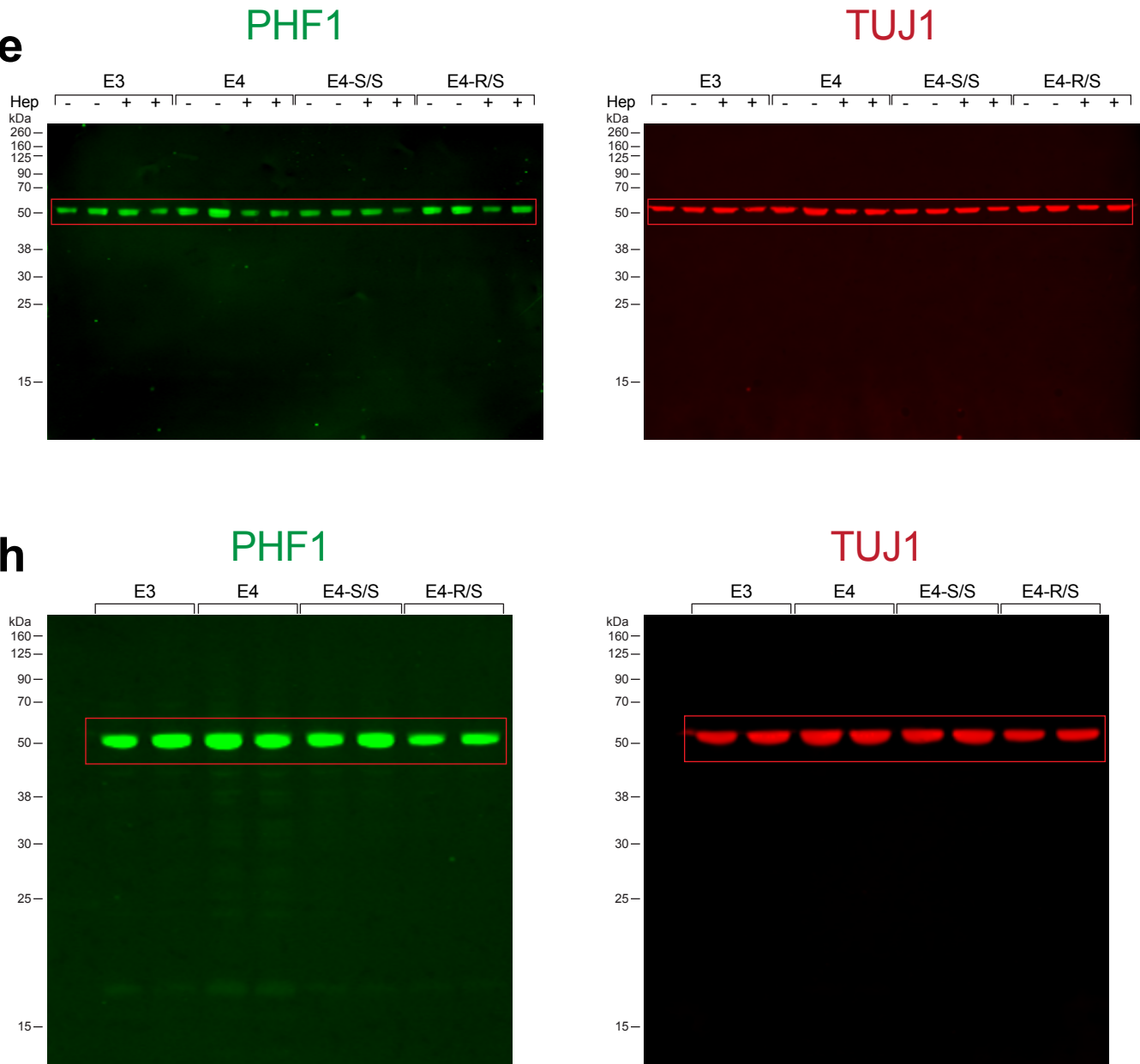

Supplement: Supplementary file 15 — Uncropped scans of WB gel image source data for Fig. 3e,h [file 41593_2023_1480_MOESM15_ESM.pdf]
